# Differentially expressed discriminative genes and significant meta-hub genes based key genes identification for hepatocellular carcinoma using statistical machine learning

**DOI:** 10.1038/s41598-023-30851-1

**Published:** 2023-03-07

**Authors:** Md. Al Mehedi Hasan, Md. Maniruzzaman, Jungpil Shin

**Affiliations:** 1 School of Computer Science and Engineering, The University of Aizu, Aizuwakamatsu, Fukushima 965-8580 Japan; 2 Department of Computer Science and Engineering, Rajshahi University of Engineering & Technology, Rajshahi, 6204 Bangladesh; 3 Statistics Discipline, Khulna University, Khulna, 9208 Bangladesh

**Keywords:** Cancer, Computational biology and bioinformatics, Biomarkers

## Abstract

Hepatocellular carcinoma (HCC) is the most common lethal malignancy of the liver worldwide. Thus, it is important to dig the key genes for uncovering the molecular mechanisms and to improve diagnostic and therapeutic options for HCC. This study aimed to encompass a set of statistical and machine learning computational approaches for identifying the key candidate genes for HCC. Three microarray datasets were used in this work, which were downloaded from the Gene Expression Omnibus Database. At first, normalization and differentially expressed genes (DEGs) identification were performed using limma for each dataset. Then, support vector machine (SVM) was implemented to determine the differentially expressed discriminative genes (DEDGs) from DEGs of each dataset and select overlapping DEDGs genes among identified three sets of DEDGs. Enrichment analysis was performed on common DEDGs using DAVID. A protein-protein interaction (PPI) network was constructed using STRING and the central hub genes were identified depending on the degree, maximum neighborhood component (MNC), maximal clique centrality (MCC), centralities of closeness, and betweenness criteria using CytoHubba. Simultaneously, significant modules were selected using MCODE scores and identified their associated genes from the PPI networks. Moreover, metadata were created by listing all hub genes from previous studies and identified significant meta-hub genes whose occurrence frequency was greater than 3 among previous studies. Finally, six key candidate genes (TOP2A, CDC20, ASPM, PRC1, NUSAP1, and UBE2C) were determined by intersecting shared genes among central hub genes, hub module genes, and significant meta-hub genes. Two independent test datasets (GSE76427 and TCGA-LIHC) were utilized to validate these key candidate genes using the area under the curve. Moreover, the prognostic potential of these six key candidate genes was also evaluated on the TCGA-LIHC cohort using survival analysis.

## Introduction

Hepatocellular carcinoma (HCC) is the 3rd leading cause of cancer deaths globally^1^. Globally, more than of 80% liver cancers are responsible for HCC^2^ and its prevalence is high in males compared to females^3^. It usually occurs in people aged 30–50 years^3^. Different factors such as hepatitis B or hepatitis C^4,5^, alcohol abuse, smoking, obesity, and type 2 diabetes (T2D) were significantly associated with HCC^6^. Among them, Hepatitis B is one of the prominent risk factors for the development of HCC, responsible for 50% of cases^7^. Despite various treatment approaches, namely radiotherapy, chemotherapy, and target therapy have been commonly used to improve the prognosis and recurrence of HCC. Nevertheless, the survival rate of HCC patients is still low^8^. As a result, the risks of cancer death are still increased due to the lack of early detection and diagnosis of genes and limited treatment facilities. Therefore, it is essential to develop a system for identifying the key or core genes for early detection and better prognosis of HCC.

Recently, bioinformatics analysis has been widely utilized to determine the key prognostic genes or biomarkers as well as their associated molecular pathways for multiple cancers, including HCC^8–58^. Zhou et al.^35^ identified 15 prognostic biomarkers as well as their associated gene ontology (GO) enrichment and Kyoto Encyclopedia of Genes and Genomes (KEGG) pathway using bioinformatics analysis. Chen et al.^39,59,60^ also identified 11 potential biomarkers that can play crucial roles in the development and progression of HCC patients. Qiang et al.^40^ proposed five core genes which were significantly associated with early diagnosis and poor prognosis of HBV-HCC. Wang et al.^41^ identified 36 hub DEGs and illustrated that 10 candidate genes out of the 36 have significant effect on the tumorigenesis and progression of HCC. Among them, eight candidate genes were inversely related to the survival rate of HCC patients. Dai et al.^61^ proposed a prognostic model for predicting the prognosis of HCC patients. They identified 17 genes that were potentially associated with the prognosis of HCC patients. These 17 genes were used to make a prognostic model using the Cox hazard regression model and validated its performance using the TCGA and GSE14520 datasets. They showed that six genes were involved in the prognosis of HCC patients. Most researchers simply used hub genes derived from the PPI network to identify the key or core genes. One of the major challenges in studying genetic data was the identification of relevant biomarkers or genes. Recently, machine learning (ML)-based techniques have gained more attraction to address this problem^59,60,62–66^. Despite the fact that several studies have been carried out for the identification and development of potential candidate genes for HCC^8–58,67^, it remains a challenging issue and still has some scope for more research for the identification of potential genes as well as understanding molecular mechanisms for the development, pathogenies, and progression of HCC.

**Figure 1:**
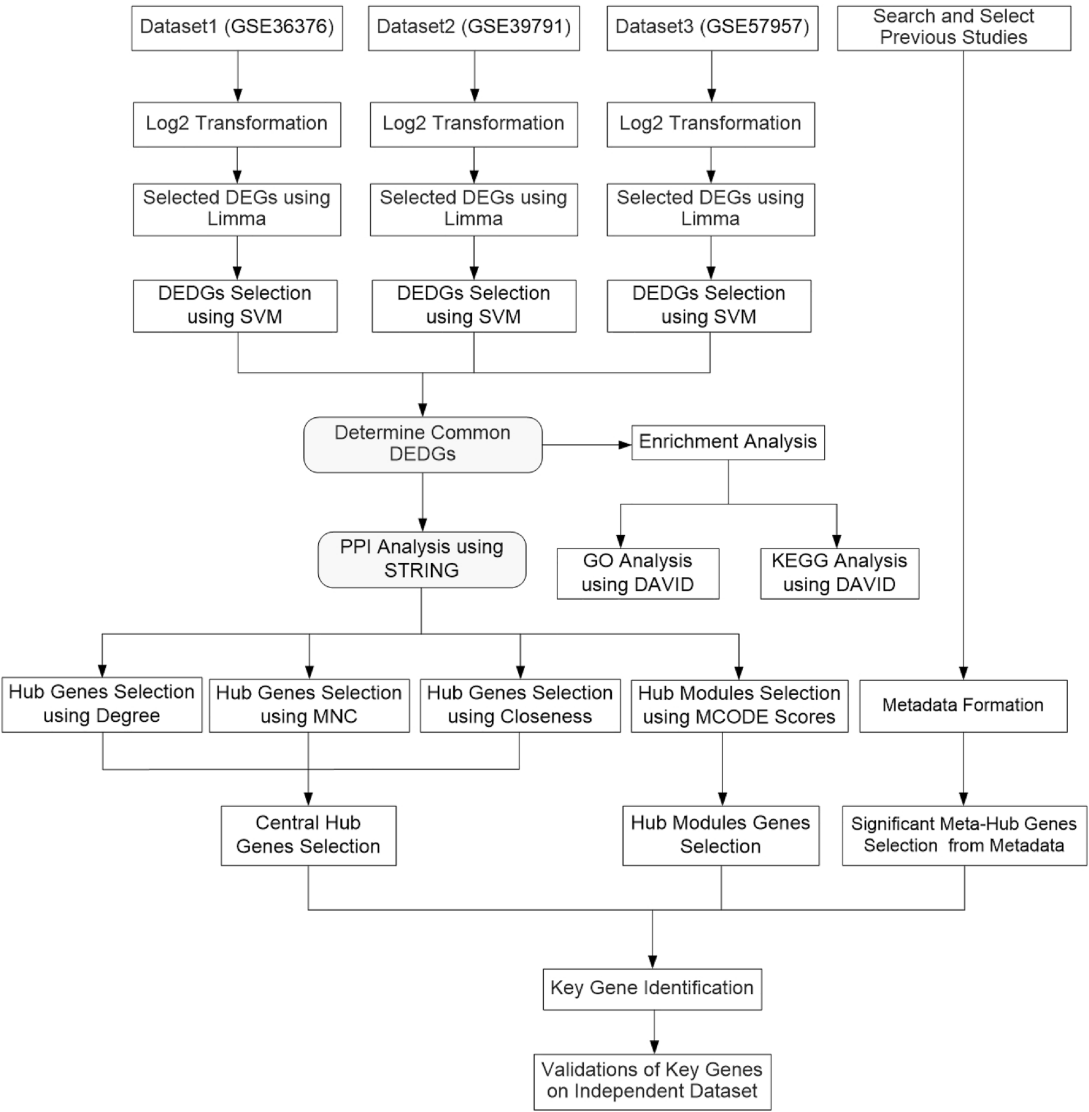
Flowchart of proposed system for the identification of key candidate genes for HCC.

In this work, we used three microarray gene expression (MGE) datasets as training sets to determine the key or core candidate genes for HCC. First, we selected individual DEGs for three datasets. Secondly, support vector machine (SVM) with radial basis function (RBF) was implemented on the identified DEGs from each of the three datasets and calculated the classification accuracy of each DEG. We selected the DEGs from each of the three datasets that provided a classification accuracy of more than 80.0%. At the same time, the overlapping or shared DEGs were identified from three datasets. These overlapping or shared DEGs were called differentially expressed discriminative genes (DEDGs). Thirdly, DAVID was used to perform enrichment analysis on common DEDGs. Fourthly, PPI networks were constructed using STRING and visualized using Cytoscape. Then the hub genes were identified using degree, maximum neighborhood component (MNC), maximal clique centrality (MCC), closeness, and betweenness on the basis of cytoHubba. After that, the central hub genes were determined by overlapping or shared hub genes from the degree, MNC, MCC, centralities of closeness, and betweenness. Molecular Complex Detection (MCODE) was performed for cluster or module analysis and determined the important or significant modules as well as their associated genes. Moreover, the significant meta-hub genes were determined from meta-hub genes, which were extracted from existing studies. The key or core candidate genes were determined among the central hub genes, potential module hub genes, and significant meta-hub genes, which can be easily discriminated against in HCC patients compared to healthy controls. Furthermore, we used another two independent test datasets for the validation as well as to show the discriminative power of the key candidate genes. We also performed a survival analysis of the identified key candidate genes for HCC patients. Therefore, the overall flowchart of our proposed system to determine key candidate genes for HCC is presented in Fig. 1.

## Results

### Identification of DEGs from each dataset

**Figure 2:**
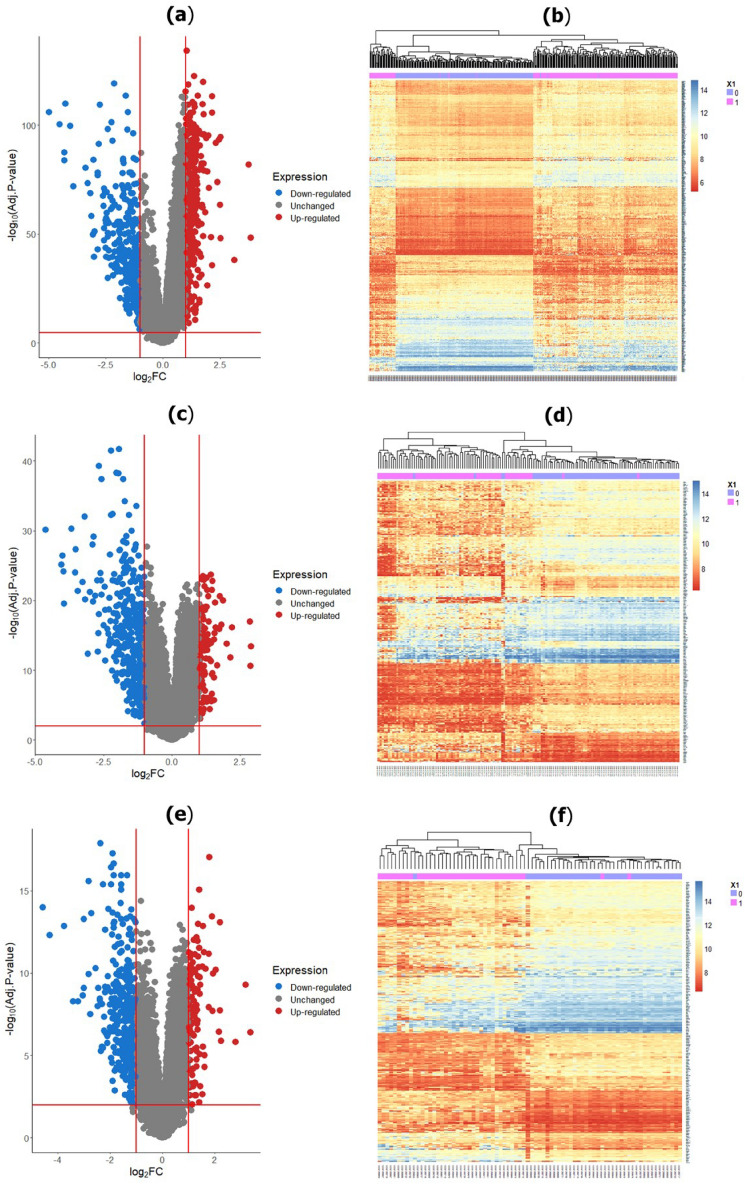
Volcano plot and heatmap of DEGs for each GEO dataset were generated using “ggplot2” version 3.3.6 package^110^ ( https://cran.r-project.org/package=ggplot2) and “NMF” version 0.24.0 package^111^ (https://cran.r-project.org/package=NMF) in R . (**a**) Volcano plot and (**b**) heatmap of GSE36376 dataset; (**c**) Volcano plot and (**d**) heatmap of GSE39791 dataset; (**c**) Volcano plot and (**d**) heatmap of GSE57957. Dodger blue represents down-regulated, gray represents no significant genes, and fire brick represents up-regulated DEGs.

We implemented limma for identifying DEGs from each of the three GEO datasets (GSE36376, GSE39791, and GSE57957). Using the threshold of $|log_2 FC|{>1}$, and adj.p-value < 0.01, we identified 699 (up-regulated: 431 vs. down-regulated: 268), 428 (up-regulated: 88 vs. down-regulated: 340 DEGs), and 413 DEGs (up-regulated: 107; down-regulated: 306) DEGs between HCC and healthy controls from GSE36376, GSE39791, and GSE57957 datasets and their volcano plots and heatmap were presented in Fig. 2.

### Identification of common DEDGs using SVM

**Figure 3:**
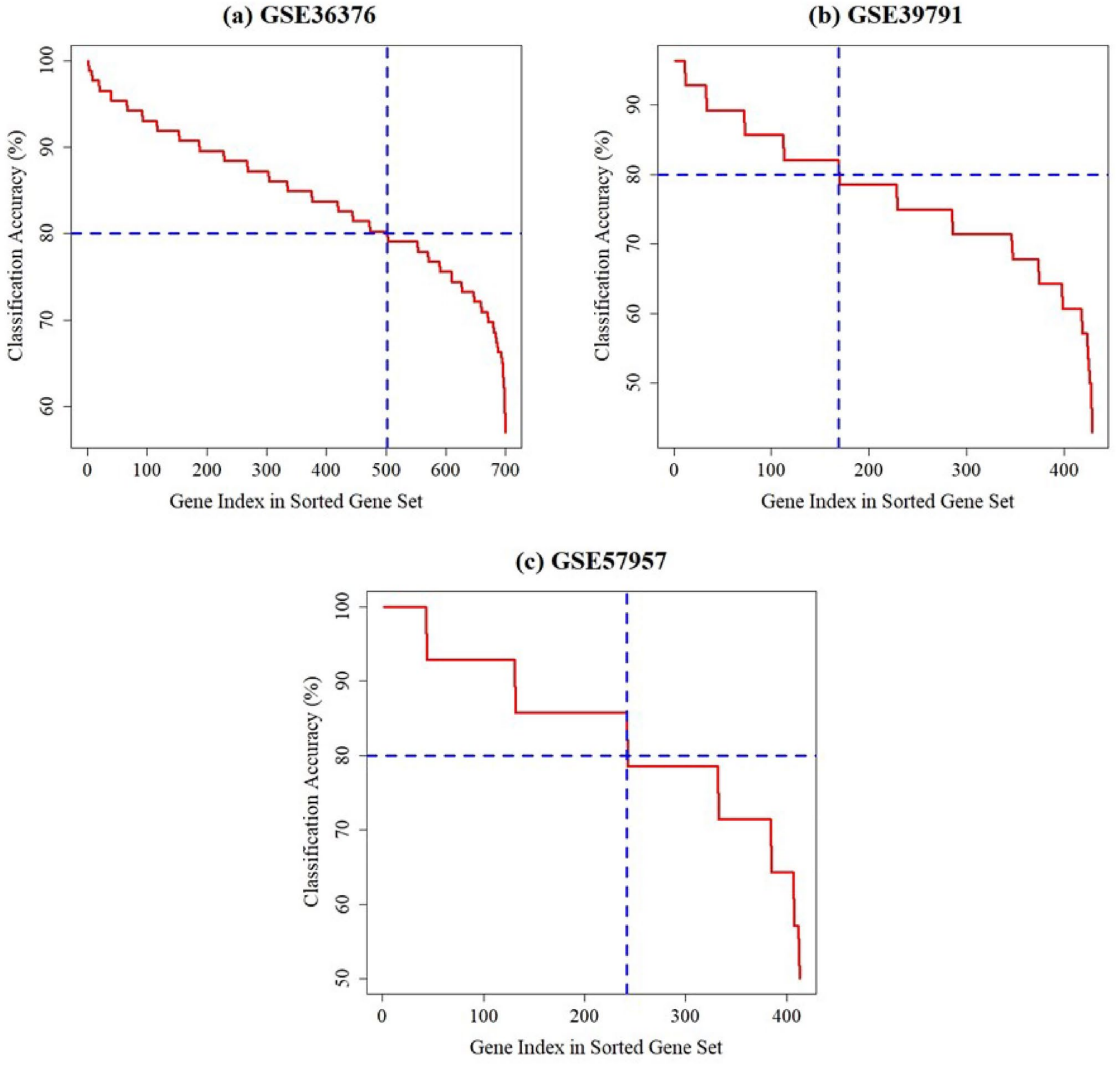
Classification accuracy of individual genes using SVM for three GEO datasets: (**a**) GSE36376; (**b**) GSE39791, and (**c**) GSE57957.

**Figure 4:**
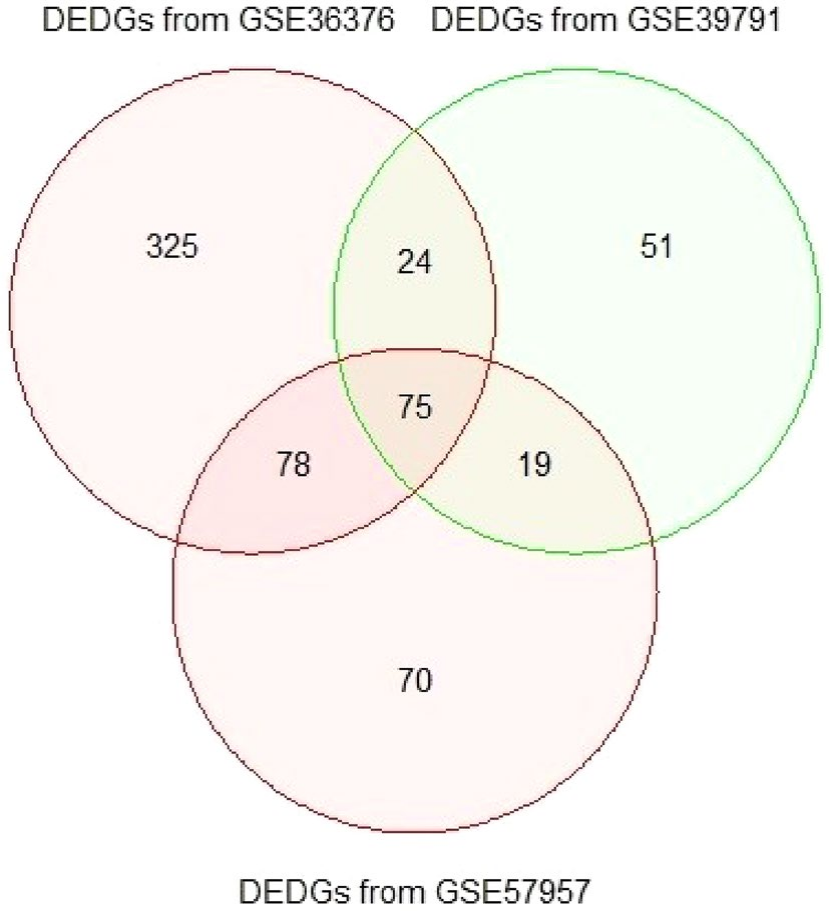
Identification of common or overlapping DEDGs among DEDGs from GSE36376, GSE39791, and GSE57957 datasets.

SVM with RBF kernel was applied on the identified DEGs (699 DEGs for GSE36376; 428 DEGs for GSE39791; and 413 DEGs for GSE57957) of each dataset in order to identify the DEDGs of HCC patients. Then, the classification accuracy was computed per gene for DEGs from each dataset. The calculation procedure is clearly discussed in the methodology section. The classification accuracies of all DEGs for individual datasets were ordered in descending order of magnitude, which is presented in Fig. 3. As shown in Fig. 3, we observed that a total of 502 from GSE36376, 169 from GSE39791, and 242 from GSE57957 DEGs were selected as DEDGs because their classification accuracy was more than or equal to 80.0%. Furthermore, 75 common DEDGs were determined among the identified DEDGS from GSE36376, GSE39791, and GSE57957 datasets, which is shown in Fig. 4.

### Enrichment analysis of common DEDGS

Enrichment analysis was conducted on 75 shared or overlapping DEDGs clearly grasp the mechanism and development of HCC. The functional characteristics of DEDGs were explored using GO and KEGG pathway analysis. The GO analysis was partitioned into three groups: biological process (BP), cellular component (CC), and morphological component. Using p-values $(< 0.05)$, we identified the significant GO and KEGG pathways, and chose the top five prominent GO terms and KEGG pathway. The top five GO terms, including BP, CC, and MF, are presented in Table 1.

**Table 1:** GO analysis of common DEDGs in terms of BP, CC, and MF. Top 5 items were selected.

Category	GO ID	Descriptions	Count	p-value
BP	GO:0042572	Retinol metabolic process	7	$3.41 \times 10^{-8}$
	GO:0071276	Cellular response to cadmium ion	5	$1.38 \times 10^{-5}$
	GO:0001523	Retinoid metabolic process	4	$1.35 \times 10^{-4}$
	GO:0071280	Cellular response to copper ion	4	$1.51 \times 10^{-4}$
	GO:0006706	Steroid catabolic process	3	$1.88 \times 10^{-4}$
CC	GO:0005576	Extracellular region	19	$3.79 \times 10^{-4}$
	GO:0070062	Extracellular exosome	18	0.00173
	GO:0005615	Extracellular space	16	0.0031
	GO:0034364	High-density lipoprotein particle	3	0.004
	GO:0016324	Apical plasma membrane	6	0.011
MF	GO:0004745	Retinol dehydrogenase activity	5	$5.81 \times 10^{-7}$
	GO:0016491	Oxidoreductase activity	9	$2.46 \times 10^{-6}$
	GO:0047023	Androsterone dehydrogenase activity	3	$3.56 \times 10^{-4}$
	GO:0047044	Androstan-3-alpha,17-beta-diol dehydrogenase activity	3	$4.56 \times 10^{-4}$
	GO:0016229	Steroid dehydrogenase activity	3	0.001

For BP-based GO terms, the common DEDGs were strongly enriched with retinol metabolic process, cellular response to cadmium ion, retinoid metabolic process cellular response to copper ion, and steroid catabolic process. Moreover, the extracellular region, extracellular exosome, extracellular space, high-density lipoprotein particle, and apical plasma membrane were found to be top CC, which were significantly enriched with common DEDGs. As shown in Table 1, MF group GO terms, including retinol dehydrogenase activity; oxidoreductase activity; androsterone dehydrogenase activity; androstan-3-alpha,17-beta-diol dehydrogenase activity; and steroid dehydrogenase activity, were mainly enriched with common DEDGs.

The study of the KEGG pathway for common DEDGs is displayed in Table 2. As shown in Table 2, the common DEDGs were significantly associated with multiple pathways such as retinol metabolism, metabolic pathways, tryptophan metabolism, steroid hormone biosynthesis, and drug metabolism-cytochrome P450.

**Table 2:** KEGG pathway analysis of common DEDGs. Top five items were selected.

Pathway ID	Descriptions	Count	p-value
hsa00830	Retinol metabolism	6	$3.28 \times 10^{-5}$
hsa01100	Metabolic pathways	21	$7.24 \times 10^{-5}$
hsa00380	Tryptophan metabolism	4	0.001
hsa00140	Steroid hormone biosynthesis	4	0.004
hsa00982	Drug metabolism-cytochrome P450	4	0.007

### PPI network construction and central hub genes identification

**Figure 5:**
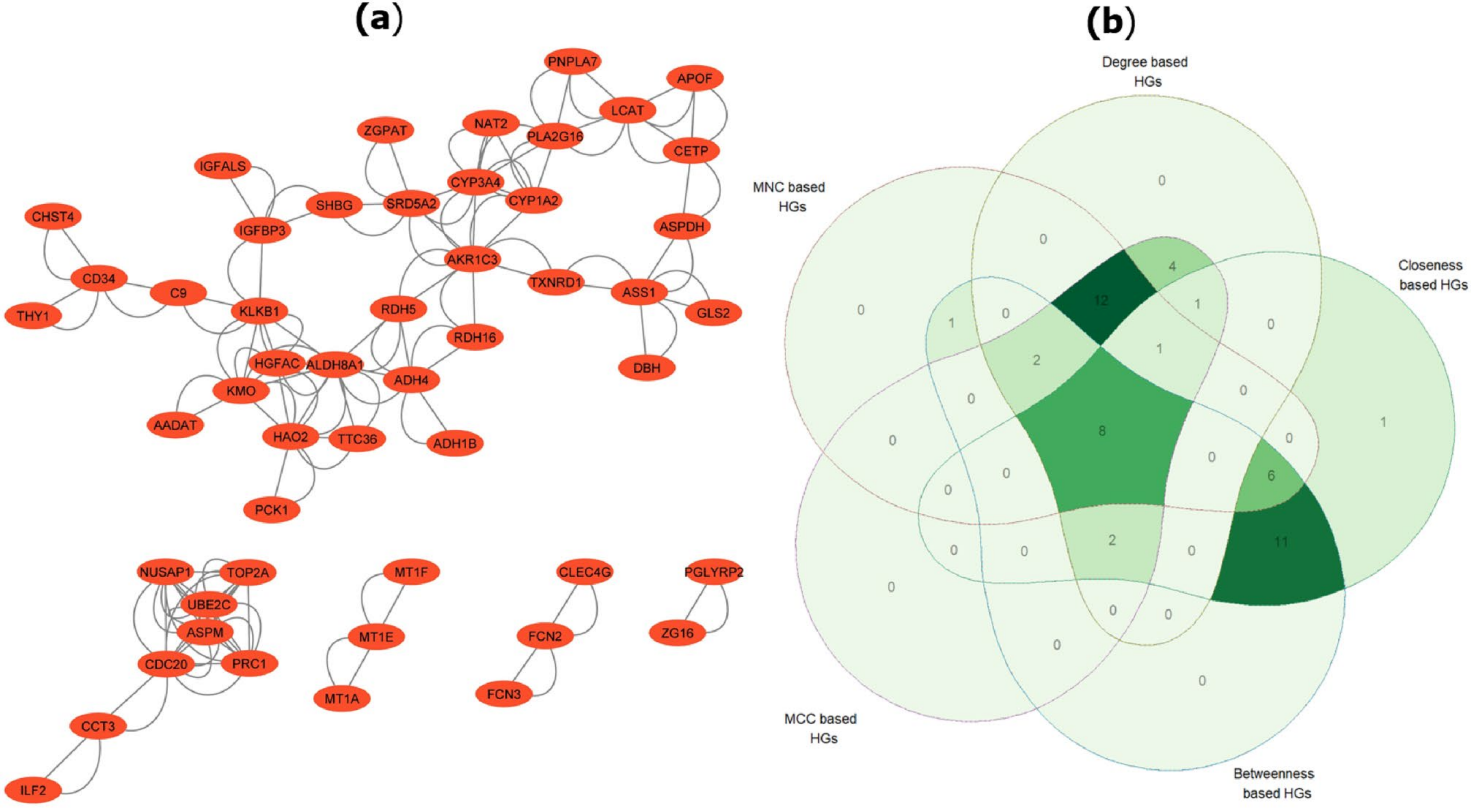
PPI network and Venn diagram for common DEDGs and central hub genes. (**a**) PPI network of common DEDGs with 51 nodes and 144 edges which was generated by Cytoscape 3.9.1^118^ (www.cytoscape.org); (**b**) identification of central hub genes among five methods (Degree, MNC, MCC, Closeness, and Betweenness based HGs). Here, HGs represent the hub genes.

STRING was utilized to build a PPI network to show the significant connections between proteins encoded by common DEDGs. Cytoscape was used to show the PPI network, which had 51 nodes and 144 edges (see Fig. 5a). Five hub gene-based identification algorithms, including the degree of connectivity, MNC, MCC, closeness, and betweenness in the Cytoscape plug-in cytoHubba, were implemented to determine the hub genes from PPI networks. Then we chose the top 30 hub genes from each algorithm. We made a Venn diagram among the five algorithms, which is shown in Fig. 5b. As shown in Fig. 5b, eight overlapping central hub genes were identified among these algorithms. These eight central hub genes were NUSAP1, TOP2A, CDC20, PRC1, UBE2C, ASPM, PNPLA7, and MT1E, which were utilized to determine the key or core genes for HCC.

### Hub modules and its associated genes identification

**Figure 6:**
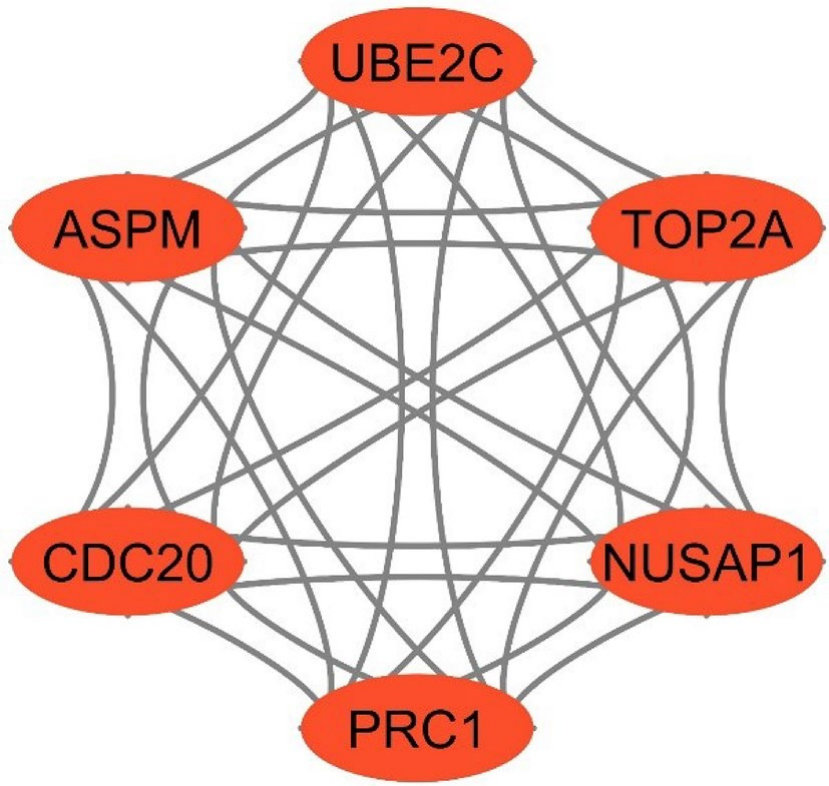
PPI network of module 1 with 6 nodes and 30 edges which was generated by Cytoscape 3.9.1^118^ (www.cytoscape.org).

Module or cluster analysis was performed using MCODE to determine the prominent modules. Three clusters or modules were generated using MCODE and provided 3–6 MCODE scores. We chose the prominent modules that provided the MCODE scores of $\ge 5$ and the number of nodes $\ge 5$. Finally, we chose module 1 as a prominent hub module that contained 6 nodes and 30 edges with the highest MCODE scores of 6 and their PPI networks were displayed in Fig. 6. The correspondence six genes were treated as hub module genes.

### Identification of significant meta-hub genes from metadata

We reviewed 52 existing studies related to gene identification of HCC patients^8–58^. We listed their hub genes in order to make metadata which were presented in Table 3. To make metadata, we extracted 10 hub genes from Maddah et al.^9^, 5 hub genes from Yan et al.^10^, 20 from Zhao et al.^11^, 7 from Zhao et al.^12^, 10 from Liu et al.^13^, 11 from Meng et al.^14^, 42 from Rosli et al.^15^, 5 from Zhang et al.^8^, 5 from Li et al.^16^, 8 from Li et al.^17^, 5 from Tian et al.^18^, 12 from Wan et al.^19^, 10 from Zhu et al.^20^, 10 from Wang et al.^21^, 9 from Zhou et al.^22^, 10 from Zhang et al.^23^, 18 from Mou et al.^24^, 8 from Wu et al.^25^, 9 from Gui et al.^26^, 10 from Wang et al.^27^, 28 from Lu and Zhu^28^, 6 from Bhatt et al.^29^, 10 from Zhang et al.^30^, 13 from Jiang et al.^31^, 20 from Zhang et al.^32^, 12 from Wu et al.^33^, 5 from Nguyen et al.^34^, 15 from Zhou et al.^35^, 6 from Yu et al.^36^, 10 from Kakar et al.^37^, 10 from Ji et al.^38^, 11 from Chen et al.^39^, 10 from Qiang et al.^40^, 10 from Wang et al.^41^, 10 from Zhang et al.^42^, 14 from Kim et al.^43^, 10 from Zhang et al.^44^, 14 from Sha et al.^45^, 10 from Chen et al.^46^, 4 from He et al.^47^, 10 from Zhang et al.^48^, 4 from Hu et al.^49^, 9 from Zhang et al.^50^, 15 from Li et al.^51^, 5 from Cao et al.^52^, 7 from Yang et al.^53^, 5 from Wang et al.^54^, 9 from Jiang et al.^55^, 16 from Li et al.^56^, 15 from Xing et al.^57^, 10 from Zhu W et al.^58^, and 20 from Dai et al.^61^. Now, we took the union of extracted hub genes and got 214 hub genes as meta-hub genes. At the same time, we also computed the frequency of each meta-hub gene depending on how many studies got that gene as hub gene and selected 52 significant meta-hub genes because their frequency was more than 3. These selected 52 significant meta-hub genes were utilized for the determination of key genes.

**Table 3:** Formation of metadata by listing hub genes from existing studies.

SN	Authors	NHG	Associated hub genes	SN	Authors	NHG	Associated hub genes
1	Maddah et al.^9^	10	BUB1, CDCA8, DLGAP5, ASPM, POLQ,CENPE, WDHD1, HELLS, TRIP13, DEPDC1	27	Nguyen et al.^34^	5	TOP2A, RRM2, NEK2, CDK1, CCNB1
2	Yan et al.^10^	5	CCNA2, PLK1, CDC20, UBE2C, AURKA	28	Zhou et al.^35^	15	DTL, CDK1, CCNB1, RACGAP1, ECT2, NEK2, BUB1B, PBK, TOP2A, ASPM, HMMR, RRM2, CDKN3, PRC1, ANLN
3	Qian et al.^11^	16	ADNP, CASP2, CBX1, CPSF6, DHX9, HCFC1, ILF3, RCC2, KANSL1, NAA40, NCOA6, RALGAPB, SENP1, SMARCD1, YEATS2	29	Yu et al.^36^	6	TOP2A, MAD2L1, CDC6, CHEK1, UBE2C, CCNB1
4	Zhao et al.^12^	7	CCNA2, CCNB1, CDK1, MAD2L1, TOP2A, RRM2, NDC80	30	Kakar et al.^37^	10	CDK1,CCNA2, CCNB1, CCNB2, BUB1, NDC80, BUB1B, NCAPG, MAD2L1, CDC20
5	Liu et al.^13^	10	CYP3A4, UGT1A6, AOX1, UGT1A4, UGT2B15, CDK1, CCNB1, MAD2L1, CCNB2, CDC20	31	Ji et al.^38^	10	CDK1, CCNB1, CCNB2, PBK, ASPM,NDC80, AURKA, TPX2, KIF2C, CENPF
6	Meng et al.^14^	11	CDK1, CCNB2, CDC20, CCNB1, TOP2A, CCNA2,PBK, MELK, TPX2, KIF20A, AURKA	32	Chen et al.^39^	11	RRM2, NDC80, ECT2, CCNB1, ASPM, CDK1,PRC1, KIF20A, DTL, TOP2A, PBK
7	Rosli et al.^15^	42	CDK1, PPAP2B, CCNA2, SQLE, CCNB1,SULTIA3, NUSAP1, MAD2L1, LCAT, TOP2A, CETP, CCNB2, CFP, KIF11,FOS, NCAPG, CDK1, CDC20, TOP2A, TTK, C7, AURKA, C6, RRM2, NDC80, ACLY, MSH2, ESR1, CENPA, NDC80, MELK, CXCL12, PBK, DTL, NR1I2, IGF1, BUB1B, HBA1, PRC1, SPTBN2, KIF2C, CYP1A2	33	Qiang et al.^40^	10	CDK1, CCNB2, CDC20, BUB1, BUB1B, CCNB1, NDC80, CENPF, MAD2L1, NUF2
8	Zhang et al.^8^	10	GMPS, ACACA, ALB, TGFB1, KRAS, ERBB2, BCL2, EGFR, STAT3, CD8A	34	Wang et al.^41^	10	CDKN3, TOP2A, UBE2C, CDC20, PBK, ASPM, KIF20A, NCAPG, CCNB2, CYP3A4
9	Li et al.^16^	5	SPP1, COL1A2, IGF1, LGALS3, LPA	35	Zhang et al.^42^	10	CCNB1, AURKA, TOP2A, NEK2, CENPF, ASPM, KIF20A, NCAPG, CCNB2, CYP3A4
10	Li et al.^17^	8	BUB1, BUB1B, CCNA2, CCNB1, CDC20, CDK1, MAD2L1, CCNB2	36	Kim et al.^43^	14	ANLN, ASPM, BUB1B, CCNB1, CDK1, CDKN3, ECT2,HMMR, NEK2, PBK, PRC1, RACGAP1, RRM2, TOP2A
11	Tian et al.^18^	5	CDC20, TOP2A, RRM2, UBE2C, AOX1	37	Zhang et al.^44^	10	CCNB1, CDC20, CCNB2, CDK1, SPC24, CENPW, ZWINT, PTTG1, AURKA, UBE2C
12	Wan et al.^19^	12	GF1, IGF2, NDC80, CDK1, CENPF, CDCA8, CCNB1, BIRC5, NCAPG, SPC25, CDCA5, CENPU	38	Sha et al.^45^	14	TOP2A, HMMR, DTL, CCNB1, NEK2, PBK, RACGAP1, PRC1, CDK1, RRM2, ECT2, BUB1B, ANLN, ASPM
13	Zhu et al.^20^	10	CDK1, TOP2A, CCNB1, CDC20, PLK1, BIRC5, CCNB2, FOS, AURKA, AURKB	39	Chen et al.^46^	10	TOP2A, CCNB2, PRC1, RACGAP1, AURKA, CDKN3, NUSAP1, ASPM, CDCA5, NCAPG
14	WANG et al.^21^	10	TOP2A, CDK1, ITGA2, PLK1, ESR1, CCNB2, AURKA, BUB1, CCNA2, BUB1B	40	He et al.^47^	4	CDK1, PBK, RRM2, and ASPM
15	Zhou et al.^22^	9	ASPM, AURKA, CCNB2, CDKN3, MELK, NCAPG, NUSAP1, PRC1, TOP2A	41	Zhang et al.^48^	10	NEK2, ANLN, TOP2A, CENPF, ASPM, CDC20, CDK1, CCNB1, ECT2, CCNB2
16	Zhang et al.^23^	10	CDK1, CCNB1, AURKA, CCNA2, KIF11, BUB1B, TOP2A, TPX2, HMMR, CDC45	42	Hu et al.^49^	4	JUN, EGR1, MYC, CDKN1A
17	Mou et al.^24^	18	TOP2A, FOS, TK1, CDC20, ESR1, CCNB2, CXCL12, FOXO1, HMMR, VWF, ACSM3, COL4A1, ZIC2, RFC4, TXNRD1, GNAO1, CYP3A4, RAP2A	43	ZHANG et al.^50^	9	ALDH2, PPTG1, CYP2C8, ADH4, ADH1B, CYP2C8, CDC20, TOP2A, CCNB2
18	Wu et al.^25^	8	CDKN3, CDK1, CCNB1, TOP2A, CCNA2, CENPE, KCCNB2, PRC1, RRM2	44	Li et al.^51^	15	TOP2A, CDK1, CCNB1, BUB1, CENPF, CCNB2, TTK, KIF2C, HMMR, MELK, CENPE, KIF20A, KIF4A, PBK, DLGAP5
19	Gui et al.^26^	4	MT1X, BMI1, CAP2, TACSTD2	45	Cao et al.^52^	5	MCM3, CHEK1, KIF11, PBK, S100A9
20	Wang et al.^27^	10	TOP2A, CDK1, NDC80, CCNB1, HMMR, CENPF, AURKA, CDKN3, FOXM1, PTTG1	46	Yang et al.^53^	7	PITX2, PNCK, GLIS1, SCNN1G, MMP1, ZNF488, SHISA9
21	Lu^28^	28	NDUFC2, NDUFS7, NDUFB1, NDUFB9, NDUFA2, NDUFB7, NDUFA11, NDUFAF6, NDUFS6, NDUFB8, MRPS28, MRPS18A, MRPL14, MRPL12, MRPL54, MRPL55, MRPL52, MRPL13, MRPL27, MRPL24, NUF2, DSN1, GADD45GIP1, CHCHD1, STAG2, PPP1CC, CKAP5, ZWINT	47	WANG et al.^54^	5	CDK1, CCNB1, CCNB2, MAD2L1, TOP2A
22	Bhatt et al.^29^	6	MSH3, DMC1, ALPP, IL10, ZNF223, HSD17B7	48	Jiang et al.^55^	9	ANLN, BIRC5, BUB1B, CDC20, CDCA5, CDK1, NCAPG, NEK2, TOP2A
23	Zhang et al.^30^	10	CDC20, CCNB1, EIF4A3, H2AFX, NOP56, RFC4,NOP58, AURKA, PCNA, FEN1	49	Li et al.^56^	16	BIRC5, BUB1, CCNB2, CDC20, CDC25C, CDK1, CEP55, CXCL12, FOS, PRC1, KIF20A, NUSAP1, KIF2C, RACGAP, SPC24, TOP2A
24	Jiang et al.^31^	13	TLR1, TLR4, TLR7, TLR8, RIPK2, YWHAZ, FOS, FOSL2, HIF1A, FASLG, CCL4, CDK1A, DDIT3	50	Xing et al.^57^	15	TOP2A, PCNA, CCNB2, AURKA, CDKN3, BUB1, RFC4, CEP55, DLGAP5, MCM2, PRC1, RACGAP1, TPX2, CDC20, MCM4
25	Zhang et al.^32^	20	CDK1, CCNB1, CCNB2, CDC20, CCNA2, AURKA, MAD2L1, TOP2A, BUB1B, BUB1, ESR1, IGF1, FTCD, CYP3A4, SPP2, C8A, CYP2E1, TAT, F9, CYP2C9	51	Zhu et al.^58^	10	UBE2C, CDK1, TK, NCAPG, TOP2A, AURKA, MAD2L1, TOP2A, BUB1B, BUB1, RAD51AP1, ASPM, PBK, DLGAP5, NUSAP1
26	Wu et al.^33^	12	TTK, NCAPG, TOP2A, CCNB1, CDK1, PRC1, RRM2, UBE2C, ZWINT, CDKN3, AURKA, RACGAP1	52	Dai et al.^61^	20	ANLN, DLGAP5, NDC80, NUSAP1, RACGAP1, PBK, ZWINT, BUB1B, TOP2A, NUF2, CCNB1, RRM2, DTL, KIF20A, CDKN3, HMMR, PRC1, CCL20, NPY1R, CXL12

### Key candidate genes identification

**Figure 7:**
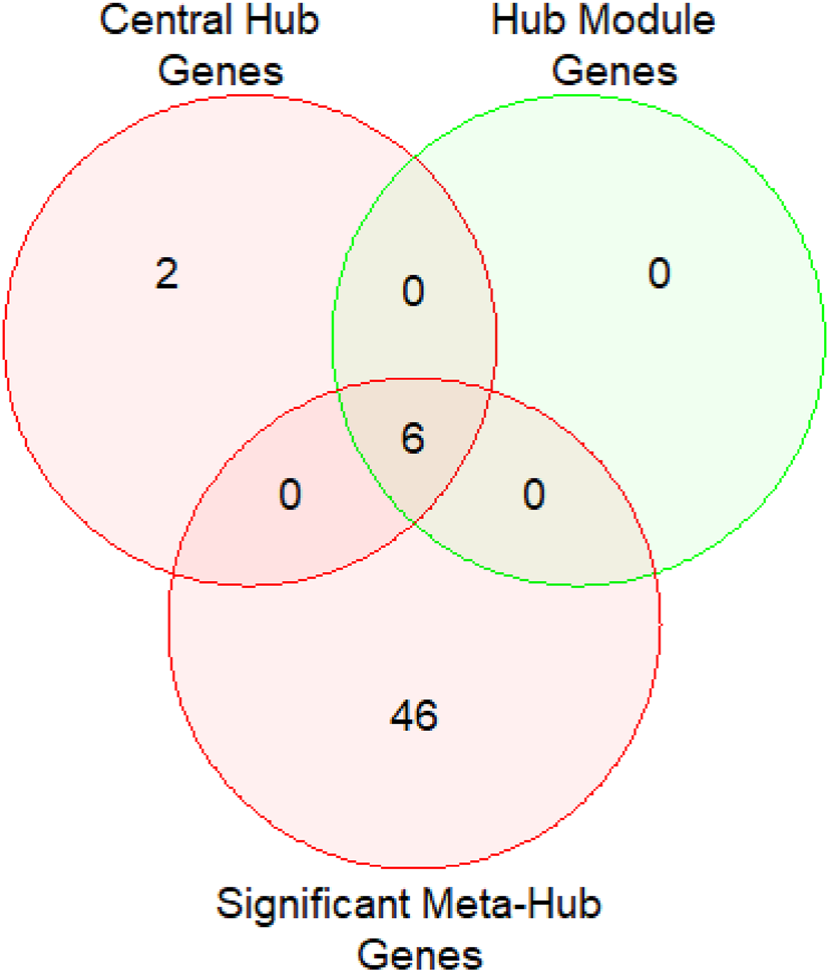
Identification of key candidate genes of HCC from central hub genes, hub module genes, and significant meta-hub genes.

Eight central hub genes were identified from five methods (degree of connectivity, MNC, MCC, closeness, and betweenness), 6 hub module genes from potential hub modules, and 52 significant meta-hub genes from meta-hub genes. Six overlapping genes were identified using the Venn diagram from these three gene identification methods, which is presented in Fig. 7. These six genes (TOP2A, CDC20, ASPM, PRC1, UBE2C, and NUSAP1) were considered as key genes, which can be easily classified into the subjects as HCC and healthy.

### Validation of key candidate genes

#### Discriminative power analysis using ROC curve

**Figure 8:**
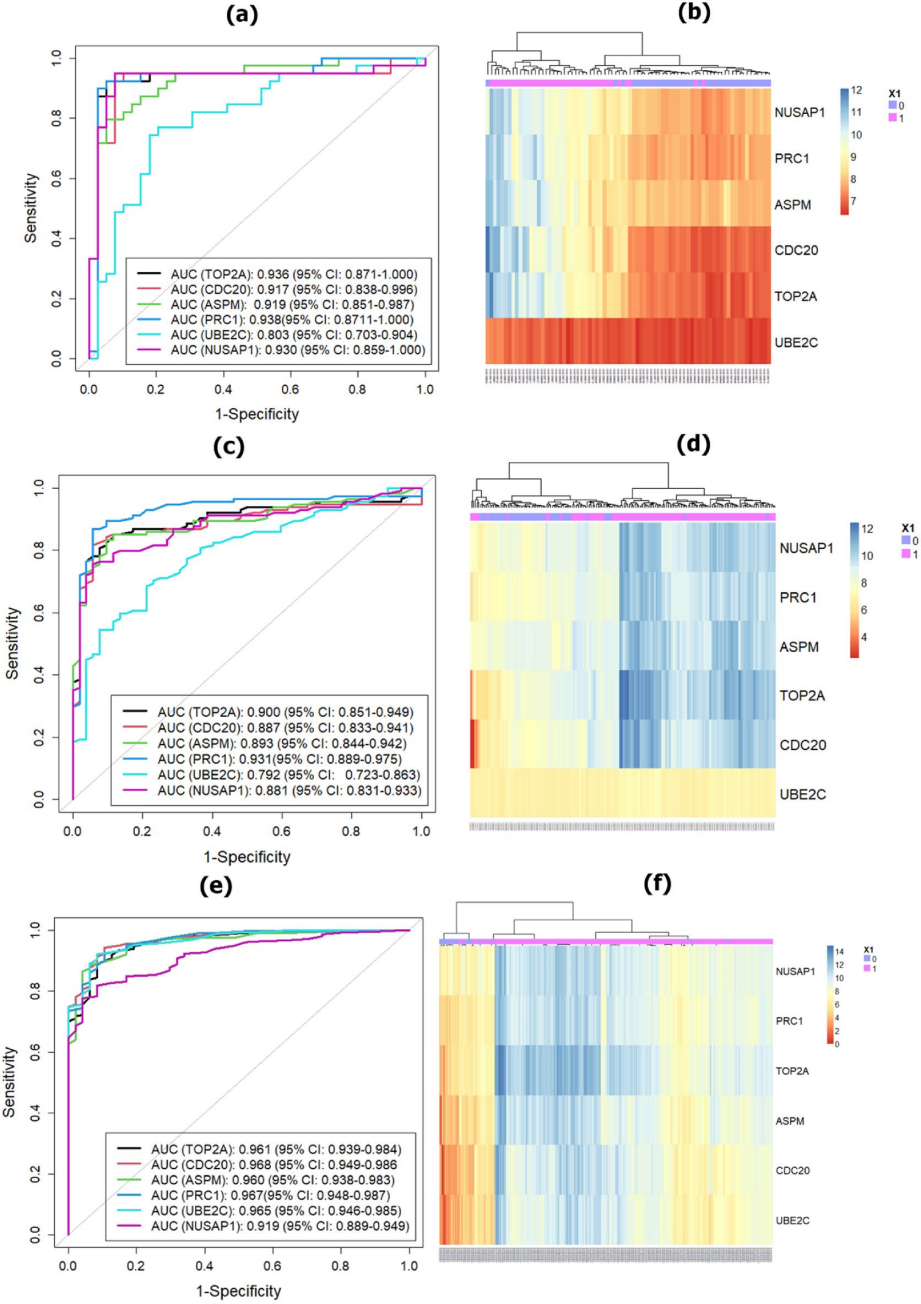
Validation of the six key candidate genes using AUC and heatmap: (**a**), (**b**) GSE57957-based training dataset; (**c**), (**d**) GSE76427-based independent test dataset; and (**e**), (**f**) TCGA-LIHC based independent test dataset. Whereas, ROC curves were generated using pROC version 1.18.0 package^121^ and heatmap was generated using “NMF” version 0.24.0 package in R^111^.

Six key or core genes (TOP2A, CDC20, ASPM, PRC1, UBE2C, and NUSAP1) were validated using AUC, computed from ROC curves. We compared the performance of two independent test datasets (GSE76427 and TCGA-LIHC) with one of our train datasets (GSE57957) in order to show the precision of the selected key candidate genes. The ROC curves of six key genes as well as their heatmap for both training and independent test datasets were illustrated in Fig. 8.

The ROC curve of six key candidate genes with their AUC values for the training dataset (GSE57957) was displayed in Fig. 8a: TOP2A (AUC: 0.936, 95% CI 0.871–1.000), CDC20 (AUC: 0.917, 95% CI 0.838–0.996), ASPM (AUC: 0.919, 95% CI 0.851–0.987), PRC1 (AUC: 0.938, 95% CI 0.871–1.000), UBE2C (AUC: 0.803, 95% CI 0.703–0.904), and NUSAP1 (AUC: 0.930, 95% CI 0.895–1.000). As displayed in Fig. 8c, the AUC values of six key or core genes were more than almost 0.780. The AUC values of six key or core genes for the GSE76427 dataset were: TOP2A (AUC: 0.900, 95% CI 0.851–0.949), CDC20 (AUC: 0.887, 95% CI 0.883–0.941), ASPM (AUC: 0.893, 95% CI 0.844–0.942), PRC1 (AUC: 0.931, 95% CI 0.889–0.975), UBE2C (AUC: 0.792, 95% CI 0.723–0.863), and NUSAP1 (AUC: 0.881, 95% CI 0.831–0.933).

Similarly, the ROC curves of six key candidate genes with their AUC values for the TCGA-LIHC-independent test dataset were presented in Fig. 8e. As presented in Fig. 8e, it was observed that six key candidate genes were provided the AUC values of more than 0.900 and their individual AUC values were as follows: TOP2A (AUC: 0.961, 95% CI 0.939–0.984), CDC20 (AUC: 0.968, 95% CI 0.949–0.986), ASPM (AUC: 0.960, 95% CI 0.938–0.983), PRC1 (AUC: 0.967, 95% CI 0.948–0.987), UBE2C (AUC: 0.965, 95% CI 0.946–0.985), and NUSAP1 (AUC: 0.919, 95% CI 0.889–0.949). Therefore, these six key genes (TOP2A, CDC20, ASPM, PRC1, UBE2C, and NUSAP1) showed strong discriminative power to classify HCC patients from healthy controls. These validations would be supported our findings and provided them more robust.

#### Survival analysis

**Figure 9:**
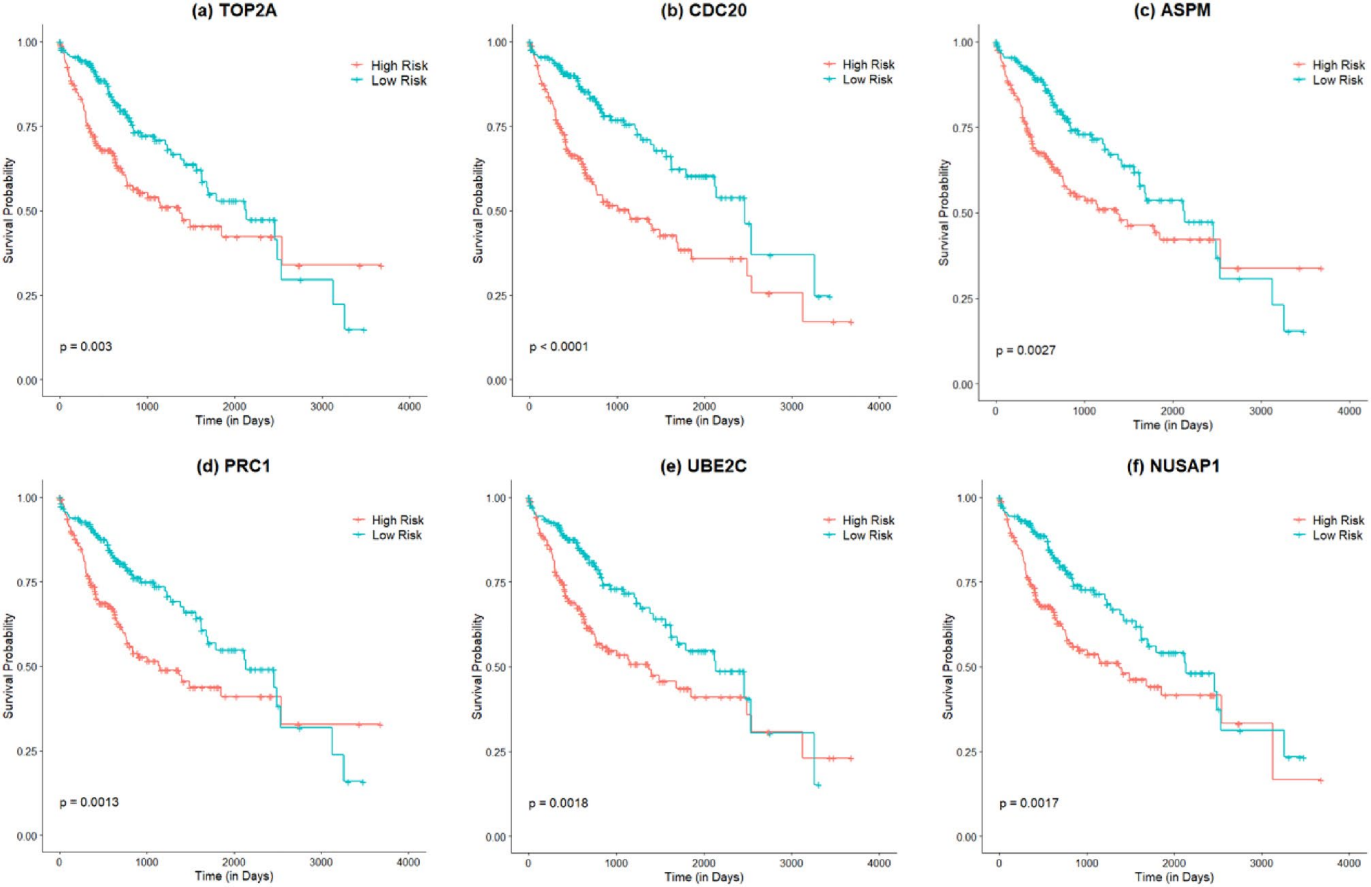
Survival analysis of six key candidate genes for HCC: (**a**) TOP2A; (**b**) CDC20; (**c**) ASPM; (**d**) PRC1; (**e**) NUSAP1; and (**f**) UBE2C. The horizontal axis (x-axis) represents the time to event (in days) and the vertical axis (y-axis) represents survival probability. The HCC patients were divided into two groups: high-risk and low-risk and assigned a color. The red line designates the samples with high risk, and the green line represents the samples with low risk. $\hbox {p} < 0.05$ indicates a statistically significant difference in mortality between groups. The survival plots were generated using the “Survfit” package in R^122^.

In this work, we adopted survival analysis of six key candidate genes (TOP2A, CDC20, ASPM, PRC1, NUSAP1, and UBE2C) using univariate Cox regression in R and its results are presented in Fig. 9. As shown in Fig. 9, we observed that our identified six key candidate genes for HCC patinets such as TOP2A, CDC20, ASPM, PRC1, NUSAP1, and UBE2C were strongly associated with the survival status of HCC patients ($\hbox {p}<0.05$). So, the over-expression levels of TOP2A, CDC20, ASPM, PRC1, NUSAP1, and UBE2C had poor survival periods compared to lower expression levels of that key candidate genes.

## Discussion

In this work, we assessed three datasets, namely GSE36376, GSE39791, and GSE57957, to detect the DEGs for HCC patients. We determined 699, 428, and 413 DEGs using “limma” from the GSE36376, GSE39791, and GSE57957 datasets, which were illustrated in Fig. 2. Moreover, we implemented SVM to determine the DEDGs from individual datasets (see in Fig. 3) and selected overlapping or shared 75 DEDGs among the identified DEDGS from GSE36376, GSE39791, and GSE57957 datasets, which were clearly shown in Fig. 4. At the same time, enrichment analysis was executed on overlapping or shared DEDGs to clear understand their better exploration and molecular mechanism (see in Table 1). We found that the potential BP functional categories were strongly related to the development and progression of HCC patients. Retinol and retinoid metabolic processes have been linked to a variety of liver diseases, including fatty liver disease, which leads to HCC^68,69^. The rest of the BP categories were also enriched with common DEDGs, which also coincided with existing studies, like cellular response to cadmium ion^42,57,70^, cellular response to copper ion^36,70^, and steroid catabolic process^42^.

The top 5 GO terms were significantly enriched with common DEDGS, which were also consistent with previous results, such as extra cellular region^35,37,38,57^, extracellular exosome^37,38^, extracellular space^37,38,57^, high-density lipoprotein particle^57^, and apical plasma membrane^53^. In the case of MFs, common DEDGs were also enriched with top five GO terms. Existing studies supported these enrichment factional categories, including retinol dehydrogenase activity^14^, and oxidoreductase activity^37,38,42^. We also analyzed KEGG pathways and chose five pathways that were closely related to our overlapping DEDGs (see in Table 2). Different existing studies supported our findings, such as retinol metabolism^35,37,38,40,43,70^, metabolic pathways^37,38^, tryptophan metabolism^38,42,70^, steroid hormone biosynthesis^42,70^, and drug metabolism-cytochrome P450^35,42,70^.

A PPI network was built with shared DEDGs using Cystoscape (see in Fig. 5a and then eight central hub genes (NUSAP1, TOP2A, CDC20, PRC1, UBE2C, ASPM, PNPLA7, and MT1E) were identified from five hub gene selection methods, which were presented in Fig. 5b. The potential modules were identified using MCODE scores and module 1 was identified due to having the highest MCODE scores. We selected six hub module genes from module 1 as well as constructed their PPI network (see in Fig. 6). In addition, we examined 52 papers and took the hub genes from earlier studies^8–58^ in order to make metadata. At the same time, we listed 214 meta-hub genes by taking the union of extracted hub genes, which were presented in Table 3. We selected 52 significant meta-hub genes from the list of meta-hub genes whose frequency was greater than 3. Finally, we identified the six shared genes (TOP2A,CDC20, ASPM, PRC1, UBE2C, and NUSAP1) by intersecting central hub genes, hub module genes, and significant meta-hub genes, extracted from the earlier studies, known as key relevant or candidate genes, which were clearly depicted in Fig. 7. We validated these key relevant or candidate genes using AUC for one training and two independent test datasets (see Fig. 8). We observed that these six key relevant or candidate genes had high discriminative power for the differentiation of HCC patients.

TOP2A is a cell cycle-related gene that encoded a DNA topoisomerase which controls and alters the topologic states of DNA during transcription. TOP2A overexpression has been identified as a core or potential biomarker for ovarian cancers^71^, glioma^72^, and lung cancers^73^. A study showed that TOP2A overexpression in HCC patients was significantly correlated with progression and poor prognosis^74,75^. In the case of our study, TOP2A was also considered as a key or core gene for the progression and development of HCC. This finding was coincided with previous studies^12,14,15,18,20–25,27,32–36,39,41–43,45,46,48,50,51,54–58,61^.

CDC20 is a vital regulator of cell division in humans^76,77^. Overexpression or high expression of CDC20 has also been linked to lung cancer^78^, colorectal cancer^79^, breast cancer^80,81^, and other cancers. Moreover, CDC20 was strongly correlated with poor prognosis in gastric cancer^82^, bladder cancer^83^, and breast cancer^84^. A study revealed that CDC20 over-expression was significantly associated with HCC^85^. Another recent study demonstrated that there existed a strong relationship between CDC20 overexpression and the prognosis of HCC^86^. Our findings also showed that CDC20 was a potential key biomarker that played an crucial or essential role for the development and progression of HCC. Different existing studies also supported our findings^10,13,14,17,18,20,24,30,32,37,40,41,44,48,50,55–57^.

ASPM is a protein that have a major influence in the development of HCC. ASPM is located on chromosome 1 and band 1q31 and consists of 28 exons and 3477 amino-acid proteins^87^. Lots of studies have identified ASPM as a hub gene or key biomarker for multiple cancers^88–90^. Zhang et al.^90^ reported that ASPM can be a promising therapeutic target for liver. Moreover, ASPM overexpression was strongly correlated with bladder cancer and consiered as promising predictor^91^. Our findings also illustrated that ASPM was a novel key biomarker for HCC, which was supported by the existing studies^9,22,35,38,39,41–43,45–48,58^.

PRC1 is an essential protein that is the regulator of cytokinesis^92^. The higher expression level of PRC1 was found among HCC patients than healthy controls. The overexpression of PRC1 was associated with a poor prognosis for HCC patients^93^. Our work also indicated that PRC1 was a promising or key biomarker for the development of HCC, which coincided with previous studies^15,22,25,33,35,39,42,43,45,46,56,57,61^.

Similarly, we proposed UBE2C as a key or core predictor for development of HCC, which was supported by various existing studies^10,18,33,36,41,44,58^. Xiong et al.^94^ suggested UBE2C as a potential biomarker or gene for HCC. High expression of UBE2C was also found in HCC than healthy subjects^95^. UBE2C is not play a crucial role HCC but also in variety of cancers: lung cancer, gastric cancer^96,97^.

NUSAP1 is a protein associated with the nucleolar-spindle that have a vital role in spindle microtubule organization^98^. overexpression of NUSAP1 was found in a variety of malignancies, including HCC^58,99^, colon cancer^100,101^, prostate cancer^102,103^, and cervical carcinoma^104^. Moreover, overexpression of NUSAP1 was strongly linked with poor prognosis of prostate cancer^103^ and colon cancer^101^. Another study revealed that NUSAP1 is related to HCC^105^. Roy et al.^105^ illustrated that NUSAP1 expression might rise in HCC samples with low expression levels of miRNA 193a-5p, and that this overexpression was strongly associated with a shorter patient survival time. Our findings also illustrated that NUSAP1 was one of the key candidate genes that the highest expression levels were found in HCC subjects compared to healthy subjects. These findings were consistent with existing studies^15,22,46,56,58,61^.

Moreover, two independent test datasets were also used to validate these six key candidate genes using AUC. A survival analysis was also performed of these six candidate genes for HCC patients. In both cases, our identified six key candidate genes (TOP2A, CDC20, ASPM, PRC1, UBE2C, and NUSAP1) showed significant association with the development and progression of HCC. This finding will provide evidence and new insight to physicians and readers in determining the diagnosis of HCC as well as the correlated pathway of HCC.

## Materials and methods

### Data acquisition and preprocessing

In this work, three publicly available microarray gene expression datasets with GEO accession: GSE36376^66^, GSE39791^106^, and GSE57957^107^ with GPL10558 [Illumina HumanHT-12 V4.0 expression bead chip] were used to determine the key candidate genes. Another two independent test datasets were used to validate key candidate genes. One independent dataset was taken from the GEO database with accession number: GSE76427 with GPL10558 platform^102^ and another independent test dataset was taken from the Cancer Genome Atlas (TCGA) database. Microarray gene expression datasets were downloaded from the GEO database (www.ncbi.nlm.nih.gov/geo/) and TCGA-liver hepatocellular carcinoma (TCGA-LIHC) dataset was downloaded from the TCGA database (https://portal.gdc.cancer.gov/). The datasets underwent a log2 transformation and quintile normalization. Although these datasets were taken from the publicly available GEO repository, being human data, all methods were performed in accordance with the relevant guidelines and regulations. Table 4 presents a summary of the utilized datasets.

**Table 4:** Summary of utilized HCC datasets.

Datasets	Platform	Total samples	HCC	Control
GSE36376^66^	GPL10558	433	240	193
GSE39791^106^	GPL10558	144	72	72
GSE57957^107^	GPL10558	78	39	39
GSE76427^102^	GPL10558	167	115	52
TCGA-LIHC	–	424	374	50

### Identification of DEGs from each dataset

To identify the DEGs between HCC and healthy controls, each of the selected datasets was analyzed using the “limma” package^108^ in R-software with version 4.1.2. We computed the $|log_2 FC|$ and adj. p-value of each gene from the selected dataset. “Bioconductor annotation”^109^ package was used to convert microarray data probes into gene symbols. If multiple probes were matched with a gene symbol, take the gene with their associated expression values that provided the lowest or minimum adjusted p-value. The DEGs between HCC and healthy controls were identified with a cutoff of point: $|log_2FC|>1$ and $adj. p-value<0.01$ (false discovery rate). The volcano plot of DEGs was generated using the “ggplot2 version 3.3.6” package in R^110^. Moreover, a heat map of the expression of DEGs was generated with the “NMF” version 0.24.0 package in R^111^.

### SVM-based identification of DEDGs from DEGs for each dataset

The main purpose of SVM is to identify a hyperplane in a high dimensional space^112,113^ that can easily discriminate HCC patients from healthy control patients using the following discriminate function:

1 $$\begin{aligned} f(x)=\ \sum _{i=1}^{n}{\alpha _iK(x_i,\ x_j)}+b \end{aligned}$$

where, b is the bias term.

In this study, we have used radial basis kernel, which is defined as follows:

2 $$\begin{aligned} K(x_i,\ x_j)=\text {exp}(-\gamma \Vert x_i-x_j \Vert ^2) \end{aligned}$$

We set the different values of cost (C) and gamma $(\gamma )$ and tuned these values using a grid search method and select the optimal value of C and $(\gamma )$ to improve classification accuracy. In this current study, we adopted SVM as a gene selection method, and its identification procedure is described as follows:

Step 1 Select one gene from a list of identified DEGs.
Step 2 Trained SVM-based model with five-fold cross-validation (CV) protocols.
Step 3 Calculate the classification accuracy for this selected gene.
Step 4 Repeat Step 2 to Step 3 for all identified DEGs.
Step 5 Sort the classification accuracy of all DEGs in descending order of magnitude.
Step 6 Choose the genes that will produce a classification accuracy of more than 80.0.

### Identification of common DEDGs

After selecting differentially expressed discrimination genes (DEDGs) using SVM, we identified the shared or overlapping or common DEDGs among three datasets using the following formula:

3 $$\begin{aligned} \text {Common DEDGs =}\bigcap _{i=1}^{r}{\text {Identified DEDGs from GEO Datasets}}_i \end{aligned}$$

where, r is the number of utilizing GEO dataset (here, r = 3).

### Enrichment analysis of common DEDGs

To better understand the mechanism and progression of HCC patients, we obtained enrichment analysis, including GO and KEEG analysis^114,115^ on DEDGs using DAVID version 6.8 tools^116^ (david.ncifcrf.gov). A *p*-value < 0.05 was considered for significant.

### PPI network analysis and central hub gene identification

The STRING version 11.5 software (www.string-db.org) was utilized to obtain the potential interactions among common DEDGs^117^. A protein-protein interaction (PPI) with a confidence score of $> 0.70$ and a maximum number of interactors of 0 was preserved and loaded into Cystoscape version 3.9.1^118^ to build a PPI network. The degree of connectivity, maximum neighborhood component (MNC), maximal clique centrality (MCC), centralities of closeness, and betweenness were computed using cytoHubba^119^. Then, we sorted the values of degree of connectivity, MNC, MCC, centralities of closeness, and betweenness in descending order of magnitude and chose the top 30 DEDGs, known as hub genes. The central hub genes were selected by overlapping hub genes, which were computed from the degree of connectivity, MNC, MCC, centralities of closeness, and betweenness. Mathematically, it is defined as follows:

4 $$\begin{aligned} \text {Central Hub Genes=}\bigcap _{i=1}^{hg}{\text {Hub Genes from Identification Methods}}_i \end{aligned}$$

where, hg is the number of hub gene identification methods (Here, hg=5).

### Hub modules and its associated genes identification

MCODE was used to determine the most closely connected modules from the PPI network^120^. We analyzed the modules with the following cutoff points: degree =2, cluster finding =haircut, nodes score =0.2, K-score =2, and max depth =100, respectively. We determined the potential modules that provided the MCODE with scores of $\ge 6$ and the number of nodes of $\ge 6$. Then, the hub module genes were identified using the following formula:

5 $$\begin{aligned} \text {Hub Module Genes =}\bigcup _{i=1}^{h_m}{\text {Genes from Module}}_i \end{aligned}$$

where, $h_m$ is the number of significant modules.

### Significant meta-hub genes identification from metadata

We reviewed some existing studies related to HCC-based gene identification. To make metadata, we listed their identified hub genes for HCC, called “meta-hub genes,” which can be written as follows:

6 $$\begin{aligned} \text {Meta-Hub Genes} =\bigcup _{i=1}^{m}{\text {Hub Genes from Previous Study}}_i \end{aligned}$$

where, m is the number of studies obtained from obtaining hub genes (here, m = 52).

We also counted the frequency of each meta-hub gene depending on how many studies identified that gene as a hub gene. Finally, we identified significant meta-hub genes from meta-hub genes whose frequency was greater than or equal to 3, which can be written as follows:

7 $$\begin{aligned} \text {Significant Meta-Hub Genes} =\{g_i\}; i=1,2,...,n \end{aligned}$$

where, $g_i \in \text {meta-hub gene}$ and n is the number of meta-hub genes whose frequency is $\ge 3$

### Key candidate genes identification

To identify the key candidate genes, we selected the central hub genes from the PPI network, hub module genes from significant modules, and significant meta-hub genes from existing studies. Therefore, we identified the key candidate genes for HCC using the following formula:

8 $$\begin{aligned} \text {Key Candidate Genes =}\bigcap _{i=1}^{k}{\text {Important Genes from Identification Methods}}_i \end{aligned}$$

where, k is the number of significant gene identification methods (Here, k = 3). In this work, central hub genes, hub module genes, and significant gene selection methods will be considered “Important Gene Identification Methods”.

### Validation of key candidate genes

#### Discriminative power analysis using ROC curve

In this work, we used two independent test datasets in order to validate the key candidate genes. One independent test dataset (GSE76427) was taken from the GEO database, and another independent dataset was taken from the TCGA database. The description of these independent test datasets is more clearly explained in Table 4. We validated the selected key candidate genes using the area under the curve (AUC), computed from the receiver operating characteristic curve (ROC). In ROC analysis, first, we selected one gene and class label, and then we adopted logistic regression with the leave-one-out CV protocol. We computed AUC values using the “pROC” R-package^121^. Moreover, we also compared the performances of independent test datasets with one of our training datasets (GSE57957) in order to show the precision of the selected key candidate genes.

#### Survival analysis

In this work, we used TCGA-LIHC dataset for survival analysis in order to show prognostic status of key candidate genes. We classified HCC patients into high-risk and low-risk groups on the basis of median expression level of each key candidate gene. We performed survival analysis of our identified key candidate genes using the “Survfit” package in R language^122^. A p-value < 0.05 was considered statistically significant (“Supplementary information”).

## Supplementary Information

Supplementary Information.

## Data Availability

The datasets generated and/or analyzed during the current study are available in the Gene Expression Omnibus (GEO) repository with accession numbers: GSE36376, GSE39791, GSE57957, and GSE76427 with GPL10558 platforms. One can easily download these datasets from the link: www.ncbi.nlm.nih.gov/geo/.
