# Supplementary material for: Differentially expressed discriminative genes and significant meta-hub genes based key genes identification for hepatocellular carcinoma using statistical machine learning
Source: Sci Rep. 2023 Mar 7;13:3771. doi: 10.1038/s41598-023-30851-1 (PMC9992474; doi:10.1038/s41598-023-30851-1)
Supplement: Supplementary file 1 — Supplementary Information. [file 41598_2023_30851_MOESM1_ESM.docx]

**Supplementary Materials**

In this work, we used three microarray datasets (GSE36376, GSE39791, and GSE57957) with GPL10558 platform to determine the key candidate genes. We adopted “limma” in order to determine the differential expressed genes (DEGs) from each GEO dataset. We selected the genes with the following cutoff of points: $\left| {log}_{2} FC \right|>1$ and $adj.p-value<0.01$. Using this cutoff of points, we selected 699 DEGs, 428 DEGs, and 413 DEGs from GSE36376, GSE39791, and GSE57957 datasets, respectively. The list of these DEGs for GSE36376, GSE39791, and GSE57957 datasets are presented in Table S1, Table S2, and Table S3.

Table S1: List of DEGs for GSE36376 datasets

| **Symbol** | **logFC** | **AveExpr** | **t** | **P.Value** | **adj.P.Val** | **B** |
| --- | --- | --- | --- | --- | --- | --- |
| A2M | -1.05 | 11.44 | -8.35 | 9.30E-16 | 5.43E-15 | 24.56 |
| AADAT | -1.24 | 8.18 | -22.66 | 1.52E-75 | 2.07E-73 | 161.48 |
| AATF | 1.01 | 7.84 | 25.53 | 2.09E-88 | 6.39E-86 | 191.00 |
| ABCF1 | 1.02 | 9.45 | 23.85 | 6.15E-81 | 1.15E-78 | 173.86 |
| ABLIM1 | 1.03 | 8.47 | 20.20 | 2.07E-64 | 1.44E-62 | 135.93 |
| ACAA1 | -1.16 | 10.35 | -15.69 | 2.87E-44 | 6.80E-43 | 89.73 |
| ACAA2 | -1.27 | 10.14 | -15.57 | 9.54E-44 | 2.20E-42 | 88.54 |
| ACACA | 1.22 | 7.87 | 21.70 | 3.06E-71 | 3.12E-69 | 151.61 |
| ACACB | -1.23 | 9.16 | -12.16 | 1.76E-29 | 2.00E-28 | 55.86 |
| ACAD11 | -1.23 | 9.21 | -19.04 | 3.36E-59 | 1.71E-57 | 123.98 |
| ACLY | 1.47 | 9.09 | 25.66 | 5.45E-89 | 1.77E-86 | 192.34 |
| ACOT12 | -1.20 | 8.31 | -13.35 | 2.66E-34 | 3.84E-33 | 66.90 |
| ACSL1 | -1.27 | 9.80 | -11.58 | 3.28E-27 | 3.34E-26 | 50.67 |
| ACSL3 | 1.02 | 8.74 | 16.13 | 3.41E-46 | 8.93E-45 | 94.15 |
| ACSL4 | 2.52 | 8.49 | 20.51 | 7.81E-66 | 5.90E-64 | 139.20 |
| ACSM2B | -1.02 | 9.38 | -8.80 | 3.18E-17 | 2.00E-16 | 27.89 |
| ACSM3 | -1.52 | 8.64 | -19.72 | 2.90E-62 | 1.78E-60 | 131.01 |
| ACSM5 | -1.15 | 8.19 | -12.64 | 2.09E-31 | 2.60E-30 | 60.27 |
| ACTA2 | 1.32 | 10.32 | 16.67 | 1.41E-48 | 4.10E-47 | 99.61 |
| ACTG2 | 1.71 | 7.24 | 20.54 | 5.68E-66 | 4.32E-64 | 139.52 |
| ACTN4 | 1.03 | 8.90 | 15.64 | 5.02E-44 | 1.17E-42 | 89.18 |
| ADAM15 | 1.36 | 9.26 | 20.20 | 1.97E-64 | 1.37E-62 | 135.98 |
| ADAR | 1.03 | 10.21 | 17.35 | 1.31E-51 | 4.38E-50 | 106.57 |
| ADGRG7 | -1.34 | 7.64 | -23.67 | 4.05E-80 | 7.13E-78 | 171.98 |
| ADH1A | -1.59 | 13.23 | -13.89 | 1.46E-36 | 2.34E-35 | 72.07 |
| ADH1B | -2.60 | 9.97 | -21.46 | 3.94E-70 | 3.81E-68 | 149.06 |
| ADH1C | -2.49 | 11.16 | -16.01 | 1.18E-45 | 3.00E-44 | 92.91 |
| ADH4 | -3.12 | 10.80 | -19.39 | 9.43E-61 | 5.31E-59 | 127.54 |
| ADH6 | -1.39 | 8.49 | -14.09 | 2.28E-37 | 3.79E-36 | 73.92 |
| ADIRF | -1.47 | 9.43 | -12.38 | 2.42E-30 | 2.87E-29 | 57.83 |
| AFM | -2.41 | 11.77 | -17.58 | 1.26E-52 | 4.54E-51 | 108.90 |
| AFP | 1.15 | 9.22 | 6.50 | 2.26E-10 | 9.90E-10 | 12.36 |
| AGO2 | 1.02 | 8.15 | 13.08 | 3.37E-33 | 4.57E-32 | 64.37 |
| AGXT | -1.12 | 12.23 | -9.25 | 1.04E-18 | 7.06E-18 | 31.27 |
| AGXT2 | -1.22 | 7.87 | -15.26 | 2.21E-42 | 4.75E-41 | 85.41 |
| AKR1B10 | 3.14 | 9.04 | 14.72 | 4.58E-40 | 8.75E-39 | 80.10 |
| AKR1B15 | 2.16 | 7.48 | 14.15 | 1.21E-37 | 2.05E-36 | 74.55 |
| AKR1C3 | 1.71 | 11.89 | 24.83 | 2.65E-85 | 6.36E-83 | 183.88 |
| AKR1D1 | -1.87 | 8.99 | -16.55 | 5.02E-48 | 1.43E-46 | 98.35 |
| AKR7A3 | -1.80 | 10.89 | -16.20 | 1.70E-46 | 4.51E-45 | 94.84 |
| ALDH1L1 | -1.08 | 10.32 | -8.13 | 4.56E-15 | 2.57E-14 | 22.99 |
| ALDH2 | -1.21 | 11.10 | -16.09 | 5.35E-46 | 1.39E-44 | 93.70 |
| ALDH6A1 | -1.20 | 10.73 | -13.27 | 5.66E-34 | 8.01E-33 | 66.15 |
| ALDH8A1 | -1.31 | 9.17 | -17.72 | 3.20E-53 | 1.18E-51 | 110.27 |
| ALDOA | 1.20 | 8.47 | 15.89 | 3.79E-45 | 9.40E-44 | 91.75 |
| ALDOB | -1.80 | 13.00 | -12.15 | 1.95E-29 | 2.21E-28 | 55.76 |
| ALG1L | 1.86 | 7.09 | 19.75 | 2.23E-62 | 1.37E-60 | 131.27 |
| ALPL | -1.12 | 8.64 | -11.72 | 9.81E-28 | 1.02E-26 | 51.87 |
| ANG | -1.10 | 13.17 | -12.44 | 1.40E-30 | 1.67E-29 | 58.38 |
| ANGPTL3 | -1.12 | 12.20 | -10.65 | 1.07E-23 | 9.25E-23 | 42.64 |
| ANXA10 | -1.80 | 8.95 | -18.13 | 4.40E-55 | 1.80E-53 | 114.53 |
| ANXA2 | 1.79 | 10.57 | 24.10 | 4.73E-82 | 9.52E-80 | 176.42 |
| ANXA2P1 | 1.40 | 8.46 | 16.67 | 1.45E-48 | 4.21E-47 | 99.59 |
| AOX1 | -1.55 | 10.43 | -11.37 | 2.09E-26 | 2.04E-25 | 48.83 |
| AP3B1 | 1.14 | 9.08 | 25.77 | 1.69E-89 | 5.69E-87 | 193.51 |
| APCS | -1.12 | 12.38 | -8.50 | 3.10E-16 | 1.86E-15 | 25.64 |
| APH1A | 1.11 | 9.76 | 25.16 | 9.38E-87 | 2.44E-84 | 187.21 |
| APOA5 | -1.77 | 9.95 | -15.35 | 9.04E-43 | 1.99E-41 | 86.30 |
| APOC4 | -1.44 | 11.20 | -10.48 | 4.84E-23 | 4.05E-22 | 41.15 |
| APOF | -3.02 | 9.83 | -25.25 | 3.40E-87 | 9.31E-85 | 188.22 |
| AQP9 | -1.58 | 11.35 | -10.53 | 2.95E-23 | 2.49E-22 | 41.64 |
| ARG1 | -1.04 | 11.28 | -9.30 | 6.92E-19 | 4.72E-18 | 31.67 |
| ARHGEF2 | 1.26 | 9.26 | 15.01 | 2.57E-41 | 5.25E-40 | 82.97 |
| ARHGEF3 | 1.00 | 8.00 | 14.00 | 5.41E-37 | 8.84E-36 | 73.06 |
| ARID3A | 1.01 | 7.52 | 12.32 | 4.05E-30 | 4.74E-29 | 57.32 |
| ARL2 | 1.07 | 8.71 | 15.52 | 1.54E-43 | 3.53E-42 | 88.06 |
| ASAP1 | 1.15 | 8.65 | 15.79 | 1.08E-44 | 2.61E-43 | 90.71 |
| ASNS | 1.12 | 6.81 | 12.06 | 4.28E-29 | 4.76E-28 | 54.98 |
| ASPDH | -1.50 | 8.27 | -17.66 | 5.72E-53 | 2.09E-51 | 109.69 |
| ASPG | -1.66 | 7.83 | -21.12 | 1.42E-68 | 1.23E-66 | 145.49 |
| ASPM | 1.59 | 7.77 | 26.71 | 1.29E-93 | 5.81E-91 | 202.96 |
| ASPSCR1 | 1.03 | 10.08 | 12.91 | 1.68E-32 | 2.20E-31 | 62.78 |
| ASS1 | -1.74 | 11.85 | -22.37 | 3.04E-74 | 3.68E-72 | 158.50 |
| ATF5 | -1.00 | 10.85 | -7.41 | 6.86E-13 | 3.46E-12 | 18.05 |
| ATIC | 1.08 | 10.04 | 23.21 | 4.79E-78 | 7.71E-76 | 167.23 |
| ATOH8 | -1.03 | 7.57 | -18.93 | 1.13E-58 | 5.58E-57 | 122.77 |
| ATP1A1 | 1.06 | 8.97 | 18.31 | 6.71E-56 | 2.84E-54 | 116.41 |
| ATP2A2 | 1.06 | 8.88 | 23.64 | 5.38E-80 | 9.36E-78 | 171.70 |
| ATP6AP1 | 1.29 | 10.48 | 23.07 | 2.05E-77 | 3.14E-75 | 165.78 |
| ATP6V1C1 | 1.33 | 7.52 | 23.24 | 3.47E-78 | 5.65E-76 | 167.55 |
| ATP6V1F | 1.13 | 10.02 | 30.10 | 3.07E-108 | 4.84E-105 | 236.50 |
| AURKA | 1.52 | 7.48 | 22.70 | 9.97E-76 | 1.37E-73 | 161.91 |
| AURKB | 1.02 | 6.32 | 19.96 | 2.41E-63 | 1.58E-61 | 133.49 |
| AZGP1 | -1.33 | 10.43 | -13.48 | 8.10E-35 | 1.20E-33 | 68.08 |
| B4GALT3 | 1.04 | 8.04 | 26.28 | 1.00E-91 | 3.79E-89 | 198.62 |
| BAIAP2L1 | 1.14 | 7.96 | 15.44 | 3.61E-43 | 8.13E-42 | 87.21 |
| BAIAP2L2 | 1.36 | 6.90 | 14.19 | 8.48E-38 | 1.44E-36 | 74.90 |
| BBOX1 | -2.36 | 8.64 | -18.66 | 1.88E-57 | 8.65E-56 | 119.97 |
| BCAP31 | 1.01 | 10.42 | 20.97 | 6.62E-68 | 5.63E-66 | 143.96 |
| BCHE | -1.83 | 9.91 | -14.84 | 1.39E-40 | 2.73E-39 | 81.29 |
| BCL2L12 | 1.11 | 7.68 | 22.54 | 4.82E-75 | 6.22E-73 | 160.34 |
| BEX2 | 1.01 | 7.47 | 11.15 | 1.45E-25 | 1.37E-24 | 46.91 |
| BHMT | -2.35 | 11.30 | -15.75 | 1.58E-44 | 3.80E-43 | 90.33 |
| BLVRA | 1.06 | 7.96 | 16.50 | 7.93E-48 | 2.24E-46 | 97.89 |
| BOLA2 | 1.63 | 8.94 | 26.81 | 4.54E-94 | 2.15E-91 | 204.00 |
| BOP1 | 1.54 | 8.81 | 18.86 | 2.26E-58 | 1.10E-56 | 122.08 |
| BRSK1 | 1.47 | 7.76 | 18.63 | 2.36E-57 | 1.08E-55 | 119.74 |
| BSG | 1.38 | 9.67 | 17.71 | 3.40E-53 | 1.26E-51 | 110.20 |
| C19orf48 | 1.10 | 8.17 | 15.34 | 9.46E-43 | 2.07E-41 | 86.25 |
| C1orf131 | 1.00 | 8.07 | 17.21 | 5.89E-51 | 1.91E-49 | 105.07 |
| C1R | -1.27 | 9.56 | -10.35 | 1.42E-22 | 1.16E-21 | 40.08 |
| C4BPA | -1.09 | 12.45 | -8.49 | 3.35E-16 | 2.00E-15 | 25.56 |
| C6 | -2.02 | 10.87 | -16.60 | 2.88E-48 | 8.23E-47 | 98.90 |
| C6orf48 | 1.21 | 9.72 | 18.28 | 9.05E-56 | 3.80E-54 | 116.11 |
| C7 | -1.48 | 7.59 | -13.23 | 8.14E-34 | 1.14E-32 | 65.78 |
| C7orf50 | 1.01 | 10.06 | 19.37 | 1.12E-60 | 6.26E-59 | 127.37 |
| C8A | -1.90 | 11.52 | -16.91 | 1.30E-49 | 3.97E-48 | 101.99 |
| C8B | -1.26 | 10.68 | -13.27 | 5.72E-34 | 8.10E-33 | 66.13 |
| C8orf33 | 1.38 | 8.35 | 28.08 | 1.40E-99 | 1.12E-96 | 216.64 |
| C8orf4 | -1.44 | 7.99 | -15.26 | 2.08E-42 | 4.48E-41 | 85.47 |
| C9 | -2.57 | 8.85 | -16.80 | 3.70E-49 | 1.11E-47 | 100.95 |
| CA2 | -1.67 | 9.20 | -15.80 | 9.35E-45 | 2.27E-43 | 90.85 |
| CAP2 | 2.16 | 8.43 | 32.32 | 1.53E-117 | 8.03E-114 | 257.83 |
| CAPNS1 | 1.12 | 9.48 | 24.12 | 4.04E-82 | 8.17E-80 | 176.58 |
| CASP2 | 1.36 | 8.47 | 28.23 | 2.95E-100 | 2.69E-97 | 218.19 |
| CAT | -1.29 | 10.81 | -12.84 | 3.18E-32 | 4.11E-31 | 62.14 |
| CCDC34 | 1.50 | 8.66 | 23.42 | 5.51E-79 | 9.21E-77 | 169.38 |
| CCL14 | -1.07 | 9.10 | -13.34 | 2.96E-34 | 4.26E-33 | 66.79 |
| CCL20 | 2.54 | 8.09 | 17.01 | 4.33E-50 | 1.35E-48 | 103.08 |
| CCNB2 | 1.67 | 6.90 | 25.63 | 7.54E-89 | 2.43E-86 | 192.02 |
| CCT3 | 1.10 | 8.59 | 26.62 | 3.22E-93 | 1.37E-90 | 202.04 |
| CCT6P1 | 1.00 | 7.60 | 15.04 | 2.02E-41 | 4.16E-40 | 83.21 |
| CD14 | -1.15 | 10.50 | -12.63 | 2.43E-31 | 3.01E-30 | 60.12 |
| CD24 | 1.61 | 9.62 | 9.68 | 3.52E-20 | 2.57E-19 | 34.62 |
| CD34 | 1.74 | 7.46 | 28.00 | 2.89E-99 | 2.24E-96 | 215.91 |
| CDC20 | 2.47 | 7.49 | 27.41 | 1.08E-96 | 6.62E-94 | 210.02 |
| CDC25B | 1.32 | 7.63 | 21.82 | 9.40E-72 | 9.97E-70 | 152.79 |
| CDC45 | 1.01 | 6.51 | 18.40 | 2.58E-56 | 1.12E-54 | 117.36 |
| CDCA5 | 1.20 | 7.26 | 23.83 | 7.90E-81 | 1.46E-78 | 173.61 |
| CDHR2 | -1.48 | 7.61 | -22.31 | 5.49E-74 | 6.56E-72 | 157.91 |
| CDK4 | 1.17 | 8.86 | 25.88 | 5.54E-90 | 1.91E-87 | 194.62 |
| CDKN3 | 1.52 | 6.73 | 21.16 | 8.89E-69 | 7.79E-67 | 145.96 |
| CENPM | 1.23 | 6.87 | 19.56 | 1.54E-61 | 8.96E-60 | 129.35 |
| CES1 | -1.05 | 12.93 | -10.19 | 5.09E-22 | 4.07E-21 | 38.82 |
| CETP | -1.73 | 8.28 | -25.34 | 1.38E-87 | 3.90E-85 | 189.12 |
| CFHR3 | -1.30 | 8.71 | -10.66 | 9.86E-24 | 8.52E-23 | 42.73 |
| CFI | -1.21 | 10.81 | -14.94 | 5.29E-41 | 1.07E-39 | 82.25 |
| CFL1 | 1.15 | 10.50 | 26.73 | 1.01E-93 | 4.62E-91 | 203.20 |
| CHKA | 1.23 | 8.06 | 22.01 | 1.25E-72 | 1.38E-70 | 154.80 |
| CHST4 | -1.37 | 7.40 | -23.90 | 3.68E-81 | 7.00E-79 | 174.37 |
| CHTOP | 1.04 | 7.88 | 27.48 | 5.53E-97 | 3.64E-94 | 210.68 |
| CIDEB | -1.30 | 9.30 | -13.14 | 2.00E-33 | 2.75E-32 | 64.89 |
| CKAP2L | 1.14 | 6.49 | 21.48 | 3.22E-70 | 3.12E-68 | 149.27 |
| CKAP4 | 1.39 | 9.20 | 24.37 | 3.09E-83 | 6.56E-81 | 179.14 |
| CKAP5 | 1.32 | 8.19 | 28.66 | 4.40E-102 | 4.73E-99 | 222.38 |
| CKS1B | 1.12 | 8.48 | 14.05 | 3.30E-37 | 5.45E-36 | 73.55 |
| CKS2 | 1.15 | 8.46 | 17.23 | 4.71E-51 | 1.53E-49 | 105.29 |
| CLCN7 | 1.14 | 9.84 | 17.63 | 7.66E-53 | 2.78E-51 | 109.39 |
| CLDN15 | 1.58 | 8.40 | 15.38 | 6.78E-43 | 1.50E-41 | 86.58 |
| CLEC1B | -2.75 | 7.20 | -31.33 | 2.07E-113 | 5.15E-110 | 248.36 |
| CLEC4G | -2.13 | 7.68 | -33.89 | 6.33E-124 | 7.49E-120 | 272.46 |
| CLIC1 | 1.30 | 9.39 | 22.09 | 5.23E-73 | 5.91E-71 | 155.67 |
| CLN3 | 1.08 | 7.39 | 21.59 | 1.01E-70 | 1.00E-68 | 150.42 |
| CLPTM1L | 1.03 | 9.25 | 20.43 | 1.87E-65 | 1.39E-63 | 138.33 |
| CLRN3 | -1.89 | 8.26 | -15.93 | 2.74E-45 | 6.83E-44 | 92.07 |
| CLSTN1 | 1.10 | 8.31 | 15.38 | 6.90E-43 | 1.53E-41 | 86.57 |
| CMBL | -1.26 | 11.00 | -13.19 | 1.20E-33 | 1.67E-32 | 65.40 |
| CNDP1 | -1.45 | 6.73 | -23.88 | 4.58E-81 | 8.59E-79 | 174.16 |
| CNIH4 | 1.35 | 9.63 | 21.31 | 1.93E-69 | 1.78E-67 | 147.48 |
| CNOT11 | 1.21 | 9.62 | 21.72 | 2.68E-71 | 2.74E-69 | 151.74 |
| COL15A1 | 1.05 | 6.25 | 18.82 | 3.40E-58 | 1.64E-56 | 121.68 |
| COL1A1 | 1.70 | 8.04 | 18.53 | 7.09E-57 | 3.17E-55 | 118.65 |
| COL1A2 | 1.42 | 7.67 | 16.04 | 8.60E-46 | 2.20E-44 | 93.23 |
| COL4A1 | 2.17 | 9.71 | 30.40 | 1.62E-109 | 2.73E-106 | 239.43 |
| COL5A2 | 1.43 | 7.58 | 17.73 | 2.81E-53 | 1.05E-51 | 110.39 |
| COMMD5 | 1.08 | 7.87 | 25.80 | 1.35E-89 | 4.55E-87 | 193.73 |
| COPA | 1.11 | 10.27 | 28.42 | 4.54E-101 | 4.38E-98 | 220.06 |
| CORO1B | 1.03 | 8.18 | 22.98 | 5.00E-77 | 7.46E-75 | 164.89 |
| COX7B2 | 1.36 | 6.57 | 10.45 | 6.09E-23 | 5.06E-22 | 40.92 |
| CPD | 1.18 | 9.24 | 19.23 | 4.95E-60 | 2.67E-58 | 125.89 |
| CPEB3 | -1.13 | 8.60 | -18.60 | 3.34E-57 | 1.51E-55 | 119.40 |
| CPED1 | -1.58 | 8.61 | -18.36 | 4.10E-56 | 1.76E-54 | 116.90 |
| CPS1 | -1.52 | 12.39 | -10.00 | 2.45E-21 | 1.89E-20 | 37.26 |
| CPSF4 | 1.05 | 9.23 | 22.76 | 5.15E-76 | 7.20E-74 | 162.57 |
| CREB3L2 | 1.07 | 8.67 | 26.67 | 1.83E-93 | 8.03E-91 | 202.60 |
| CRHBP | -1.45 | 7.51 | -20.83 | 2.71E-67 | 2.21E-65 | 142.55 |
| CS | 1.36 | 9.25 | 29.12 | 4.50E-104 | 5.46E-101 | 226.94 |
| CSAD | -1.22 | 8.25 | -16.44 | 1.53E-47 | 4.26E-46 | 97.24 |
| CSNK1E | 1.18 | 8.55 | 17.25 | 3.75E-51 | 1.23E-49 | 105.52 |
| CSRNP1 | -1.15 | 9.40 | -15.89 | 3.79E-45 | 9.40E-44 | 91.75 |
| CSTB | 1.30 | 10.03 | 17.12 | 1.51E-50 | 4.79E-49 | 104.13 |
| CTH | -1.53 | 8.70 | -14.21 | 6.71E-38 | 1.15E-36 | 75.14 |
| CTNNA1 | 1.20 | 9.22 | 29.35 | 4.60E-105 | 6.40E-102 | 229.22 |
| CTPS2 | 1.03 | 7.88 | 19.60 | 1.04E-61 | 6.14E-60 | 129.74 |
| CTSA | 1.25 | 9.03 | 21.18 | 6.86E-69 | 6.08E-67 | 146.22 |
| CXCL12 | -1.49 | 8.71 | -18.64 | 2.21E-57 | 1.01E-55 | 119.81 |
| CYP1A2 | -4.97 | 9.56 | -30.50 | 6.05E-110 | 1.29E-106 | 240.41 |
| CYP26A1 | -1.78 | 7.69 | -22.59 | 2.91E-75 | 3.85E-73 | 160.84 |
| CYP2A6 | -2.47 | 8.87 | -15.08 | 1.30E-41 | 2.71E-40 | 83.64 |
| CYP2A7 | -1.84 | 8.11 | -14.70 | 5.68E-40 | 1.08E-38 | 79.89 |
| CYP2C8 | -3.05 | 11.94 | -17.41 | 7.36E-52 | 2.50E-50 | 107.14 |
| CYP2C9 | -2.36 | 9.66 | -14.94 | 5.44E-41 | 1.10E-39 | 82.22 |
| CYP2E1 | -3.00 | 12.59 | -15.07 | 1.42E-41 | 2.95E-40 | 83.56 |
| CYP2J2 | -1.28 | 10.66 | -14.19 | 8.28E-38 | 1.41E-36 | 74.93 |
| CYP39A1 | -1.16 | 7.21 | -22.73 | 7.24E-76 | 1.00E-73 | 162.23 |
| CYP3A4 | -3.91 | 9.85 | -22.47 | 1.03E-74 | 1.30E-72 | 159.58 |
| CYP4A11 | -2.06 | 12.61 | -14.31 | 2.65E-38 | 4.62E-37 | 76.06 |
| CYP4F2 | -1.23 | 8.45 | -14.70 | 5.71E-40 | 1.09E-38 | 79.88 |
| CYP4V2 | -1.47 | 10.50 | -15.12 | 9.19E-42 | 1.92E-40 | 83.99 |
| CYP8B1 | -2.18 | 10.42 | -12.98 | 8.90E-33 | 1.18E-31 | 63.41 |
| CYR61 | -1.38 | 8.77 | -13.90 | 1.34E-36 | 2.15E-35 | 72.16 |
| DAP3 | 1.12 | 9.31 | 22.52 | 6.21E-75 | 7.96E-73 | 160.08 |
| DARS2 | 1.01 | 7.30 | 22.55 | 4.50E-75 | 5.82E-73 | 160.41 |
| DBH | -1.53 | 6.90 | -30.49 | 7.04E-110 | 1.39E-106 | 240.26 |
| DBN1 | 1.13 | 7.02 | 14.96 | 4.32E-41 | 8.78E-40 | 82.45 |
| DBNDD1 | 1.15 | 8.15 | 14.64 | 1.03E-39 | 1.94E-38 | 79.29 |
| DBNDD2 | 1.11 | 8.74 | 17.64 | 6.72E-53 | 2.44E-51 | 109.52 |
| DCAF7 | 1.07 | 8.61 | 24.26 | 9.47E-83 | 1.96E-80 | 178.02 |
| DCN | -2.40 | 10.06 | -17.23 | 4.75E-51 | 1.54E-49 | 105.28 |
| DCTN2 | 1.00 | 9.32 | 32.94 | 4.40E-120 | 3.47E-116 | 263.65 |
| DCXR | -1.22 | 11.95 | -12.54 | 5.60E-31 | 6.81E-30 | 59.29 |
| DDX39A | 1.78 | 8.74 | 34.09 | 1.00E-124 | 1.58E-120 | 274.29 |
| DEDD | 1.12 | 8.48 | 26.79 | 5.52E-94 | 2.56E-91 | 203.80 |
| DEFB1 | -1.13 | 10.73 | -6.83 | 2.90E-11 | 1.33E-10 | 14.38 |
| DHCR7 | 1.02 | 9.34 | 13.89 | 1.49E-36 | 2.38E-35 | 72.06 |
| DKK1 | 1.21 | 7.17 | 9.86 | 7.67E-21 | 5.79E-20 | 36.13 |
| DLK1 | 1.43 | 6.64 | 7.10 | 5.25E-12 | 2.52E-11 | 16.05 |
| DMGDH | -1.15 | 8.45 | -13.69 | 1.01E-35 | 1.56E-34 | 70.15 |
| DNAJB11 | 1.21 | 10.17 | 26.92 | 1.54E-94 | 7.86E-92 | 205.07 |
| DNAJB6 | 1.10 | 8.16 | 23.89 | 4.18E-81 | 7.91E-79 | 174.25 |
| DNAJC12 | -1.20 | 8.09 | -12.27 | 6.70E-30 | 7.76E-29 | 56.82 |
| DNAJC9 | 1.03 | 7.92 | 20.37 | 3.51E-65 | 2.56E-63 | 137.71 |
| DNASE1L3 | -2.11 | 8.67 | -20.57 | 4.00E-66 | 3.07E-64 | 139.87 |
| DNMT1 | 1.40 | 8.35 | 22.61 | 2.51E-75 | 3.33E-73 | 160.99 |
| DNMT3L | -1.08 | 7.23 | -12.17 | 1.60E-29 | 1.82E-28 | 55.96 |
| DPH2 | 1.02 | 7.68 | 24.82 | 3.03E-85 | 7.19E-83 | 183.75 |
| DPYS | -1.22 | 11.00 | -11.01 | 5.01E-25 | 4.61E-24 | 45.68 |
| DUSP1 | -1.29 | 10.94 | -12.97 | 1.05E-32 | 1.38E-31 | 63.25 |
| DYNLRB1 | 1.03 | 9.06 | 29.76 | 8.54E-107 | 1.26E-103 | 233.19 |
| ECM1 | -1.56 | 7.78 | -28.55 | 1.22E-101 | 1.22E-98 | 221.37 |
| EEF1A2 | 1.03 | 7.43 | 7.49 | 3.82E-13 | 1.96E-12 | 18.63 |
| EFTUD2 | 1.12 | 8.57 | 29.21 | 1.78E-104 | 2.34E-101 | 227.87 |
| EGR1 | -1.37 | 9.81 | -14.45 | 6.69E-39 | 1.20E-37 | 77.43 |
| EIF3B | 1.05 | 9.03 | 24.51 | 6.76E-84 | 1.51E-81 | 180.65 |
| ENO3 | -1.68 | 9.06 | -14.58 | 1.77E-39 | 3.29E-38 | 78.75 |
| EPHX2 | -1.22 | 9.68 | -14.08 | 2.35E-37 | 3.91E-36 | 73.89 |
| EPRS | 1.21 | 9.08 | 28.72 | 2.35E-102 | 2.59E-99 | 223.00 |
| ERBB3 | 1.06 | 8.72 | 19.26 | 3.63E-60 | 1.98E-58 | 126.20 |
| ETNPPL | -1.14 | 7.75 | -10.90 | 1.25E-24 | 1.13E-23 | 44.77 |
| EVC2 | -1.50 | 8.01 | -19.40 | 8.38E-61 | 4.74E-59 | 127.66 |
| F12 | -1.06 | 11.41 | -10.02 | 2.21E-21 | 1.71E-20 | 37.36 |
| F9 | -2.10 | 10.17 | -18.40 | 2.60E-56 | 1.12E-54 | 117.35 |
| FADS1 | 1.76 | 7.84 | 17.27 | 3.14E-51 | 1.03E-49 | 105.70 |
| FAM134B | -1.98 | 7.69 | -18.71 | 1.14E-57 | 5.29E-56 | 120.47 |
| FAM13A | -1.23 | 8.10 | -21.07 | 2.36E-68 | 2.04E-66 | 144.98 |
| FAM189B | 1.59 | 8.07 | 28.44 | 3.74E-101 | 3.69E-98 | 220.25 |
| FAM20B | 1.08 | 8.71 | 25.35 | 1.26E-87 | 3.58E-85 | 189.21 |
| FAM50A | 1.12 | 9.70 | 18.64 | 2.26E-57 | 1.04E-55 | 119.79 |
| FAM83H | 1.41 | 7.72 | 17.76 | 2.04E-53 | 7.65E-52 | 110.71 |
| FASN | 1.37 | 11.56 | 14.80 | 2.09E-40 | 4.07E-39 | 80.88 |
| FAT1 | 1.23 | 8.75 | 15.39 | 5.67E-43 | 1.26E-41 | 86.76 |
| FBL | 1.05 | 9.00 | 21.00 | 4.70E-68 | 4.01E-66 | 144.30 |
| FBP1 | -1.95 | 11.57 | -18.78 | 5.23E-58 | 2.49E-56 | 121.25 |
| FBXL18 | 1.13 | 7.19 | 22.64 | 1.73E-75 | 2.33E-73 | 161.36 |
| FCN2 | -1.61 | 7.21 | -32.44 | 5.14E-118 | 3.48E-114 | 258.91 |
| FCN3 | -4.25 | 10.05 | -31.48 | 4.80E-114 | 1.51E-110 | 249.81 |
| FDPS | 1.12 | 8.46 | 16.85 | 2.26E-49 | 6.82E-48 | 101.43 |
| FEN1 | 1.33 | 8.31 | 25.24 | 4.14E-87 | 1.12E-84 | 188.03 |
| FETUB | -1.30 | 9.28 | -11.04 | 3.84E-25 | 3.56E-24 | 45.95 |
| FGA | -1.09 | 10.13 | -12.32 | 4.02E-30 | 4.71E-29 | 57.33 |
| FGB | -1.15 | 11.78 | -10.19 | 5.48E-22 | 4.38E-21 | 38.74 |
| FGFR4 | 1.02 | 8.93 | 12.09 | 3.35E-29 | 3.74E-28 | 55.23 |
| FGG | -1.57 | 11.43 | -12.23 | 9.34E-30 | 1.07E-28 | 56.49 |
| FKBP11 | 1.10 | 8.99 | 17.26 | 3.33E-51 | 1.09E-49 | 105.64 |
| FLAD1 | 1.15 | 8.31 | 24.88 | 1.55E-85 | 3.77E-83 | 184.42 |
| FLNC | 1.07 | 6.65 | 9.47 | 1.75E-19 | 1.24E-18 | 33.03 |
| FMO3 | -1.45 | 10.77 | -11.09 | 2.56E-25 | 2.39E-24 | 46.35 |
| FOS | -2.52 | 9.10 | -21.88 | 4.86E-72 | 5.24E-70 | 153.44 |
| FOSB | -1.82 | 8.31 | -15.49 | 2.29E-43 | 5.21E-42 | 87.66 |
| FTCD | -1.30 | 8.35 | -14.51 | 3.69E-39 | 6.72E-38 | 78.02 |
| FXYD1 | -1.42 | 7.76 | -18.62 | 2.88E-57 | 1.31E-55 | 119.55 |
| G6PC | -1.44 | 10.35 | -12.18 | 1.55E-29 | 1.76E-28 | 55.99 |
| G6PD | 1.30 | 8.03 | 15.26 | 2.10E-42 | 4.53E-41 | 85.46 |
| GAGE2B | 1.09 | 6.66 | 7.65 | 1.36E-13 | 7.11E-13 | 19.65 |
| GAGE4 | 1.02 | 6.34 | 6.97 | 1.19E-11 | 5.60E-11 | 15.25 |
| GARS | 1.24 | 9.63 | 26.61 | 3.33E-93 | 1.41E-90 | 202.01 |
| GATM | -1.27 | 10.75 | -9.33 | 5.30E-19 | 3.64E-18 | 31.94 |
| GBA | 1.34 | 9.14 | 25.48 | 3.50E-88 | 1.04E-85 | 190.49 |
| GBA3 | -2.58 | 9.37 | -20.43 | 1.74E-65 | 1.29E-63 | 138.40 |
| GBP2 | 1.56 | 9.37 | 18.37 | 3.70E-56 | 1.59E-54 | 117.00 |
| GCGR | -2.03 | 9.13 | -13.77 | 5.04E-36 | 7.88E-35 | 70.84 |
| GCNT3 | 1.17 | 6.52 | 10.15 | 7.20E-22 | 5.72E-21 | 38.47 |
| GHR | -2.19 | 9.48 | -20.32 | 5.88E-65 | 4.22E-63 | 137.19 |
| GLA | 1.21 | 7.87 | 21.36 | 1.06E-69 | 9.96E-68 | 148.08 |
| GLMP | 1.41 | 9.97 | 24.10 | 4.95E-82 | 9.92E-80 | 176.37 |
| GLS2 | -1.47 | 8.30 | -15.76 | 1.39E-44 | 3.33E-43 | 90.46 |
| GLYAT | -1.71 | 7.09 | -24.90 | 1.23E-85 | 3.05E-83 | 184.65 |
| GMFB | 1.00 | 9.08 | 22.96 | 6.67E-77 | 9.86E-75 | 164.60 |
| GMNN | 1.16 | 7.05 | 15.23 | 2.87E-42 | 6.14E-41 | 85.15 |
| GNMT | -1.30 | 8.42 | -10.84 | 2.14E-24 | 1.91E-23 | 44.24 |
| GNPAT | 1.14 | 8.38 | 22.34 | 3.91E-74 | 4.72E-72 | 158.25 |
| GNPDA1 | 1.06 | 8.71 | 20.72 | 8.87E-67 | 7.02E-65 | 141.37 |
| GPAA1 | 1.29 | 8.41 | 20.82 | 3.02E-67 | 2.46E-65 | 142.44 |
| GPC3 | 3.76 | 8.36 | 24.74 | 6.75E-85 | 1.57E-82 | 182.95 |
| GPT | -1.23 | 8.77 | -12.14 | 2.23E-29 | 2.51E-28 | 55.63 |
| GRAMD1A | 1.12 | 7.24 | 17.41 | 7.29E-52 | 2.48E-50 | 107.15 |
| GSTA1 | -1.07 | 11.09 | -6.35 | 5.36E-10 | 2.29E-09 | 11.52 |
| GSTA2 | -1.62 | 9.30 | -9.21 | 1.42E-18 | 9.51E-18 | 30.96 |
| GSTZ1 | -1.39 | 8.17 | -20.72 | 8.63E-67 | 6.84E-65 | 141.40 |
| GTPBP4 | 1.04 | 9.16 | 25.43 | 5.53E-88 | 1.62E-85 | 190.03 |
| H1F0 | 1.05 | 10.73 | 16.74 | 6.78E-49 | 2.00E-47 | 100.34 |
| H2AFY | 1.06 | 10.85 | 23.97 | 1.89E-81 | 3.64E-79 | 175.04 |
| H2AFZ | 1.14 | 9.94 | 13.32 | 3.75E-34 | 5.35E-33 | 66.56 |
| HAMP | -4.30 | 10.78 | -25.21 | 5.34E-87 | 1.42E-84 | 187.77 |
| HAO1 | -1.33 | 10.01 | -12.86 | 2.69E-32 | 3.49E-31 | 62.31 |
| HAO2 | -1.32 | 7.63 | -21.76 | 1.76E-71 | 1.84E-69 | 152.16 |
| HBA1 | -1.32 | 8.51 | -12.49 | 8.89E-31 | 1.07E-29 | 58.83 |
| HBA2 | -1.67 | 10.36 | -17.47 | 4.15E-52 | 1.44E-50 | 107.71 |
| HBB | -1.61 | 10.25 | -12.51 | 6.98E-31 | 8.45E-30 | 59.07 |
| HCFC1 | 1.13 | 8.27 | 21.45 | 4.50E-70 | 4.32E-68 | 148.93 |
| HEATR1 | 1.11 | 7.55 | 19.67 | 5.08E-62 | 3.06E-60 | 130.45 |
| HEPACAM | -1.54 | 8.21 | -13.10 | 2.97E-33 | 4.05E-32 | 64.50 |
| HGFAC | -2.44 | 8.82 | -18.90 | 1.50E-58 | 7.36E-57 | 122.49 |
| HGS | 1.45 | 9.07 | 27.99 | 3.33E-99 | 2.50E-96 | 215.78 |
| HIST1H4C | 1.03 | 11.98 | 16.08 | 5.64E-46 | 1.46E-44 | 93.65 |
| HKDC1 | 1.15 | 7.74 | 14.39 | 1.16E-38 | 2.06E-37 | 76.88 |
| HMGA1 | 1.02 | 7.85 | 17.89 | 5.11E-54 | 1.97E-52 | 112.09 |
| HMMR | 1.10 | 6.47 | 19.57 | 1.47E-61 | 8.59E-60 | 129.39 |
| HNRNPUL1 | 1.07 | 9.47 | 22.85 | 1.97E-76 | 2.85E-74 | 163.52 |
| HP | -1.47 | 13.38 | -10.76 | 4.30E-24 | 3.77E-23 | 43.55 |
| HPD | -2.11 | 10.65 | -13.81 | 3.38E-36 | 5.31E-35 | 71.24 |
| HPR | -1.12 | 10.03 | -8.50 | 3.01E-16 | 1.80E-15 | 25.67 |
| HPX | -1.61 | 12.86 | -13.13 | 2.18E-33 | 2.99E-32 | 64.80 |
| HRG | -1.56 | 12.58 | -9.81 | 1.22E-20 | 9.13E-20 | 35.67 |
| HSD11B1 | -2.40 | 10.36 | -12.66 | 1.75E-31 | 2.18E-30 | 60.45 |
| HSD17B13 | -2.51 | 8.95 | -18.94 | 1.01E-58 | 5.04E-57 | 122.88 |
| HSD17B6 | -1.64 | 11.24 | -10.02 | 2.24E-21 | 1.73E-20 | 37.35 |
| HSP90AB1 | 1.46 | 9.97 | 28.78 | 1.32E-102 | 1.48E-99 | 223.58 |
| HSPA1A | 1.10 | 9.07 | 11.10 | 2.20E-25 | 2.06E-24 | 46.50 |
| HSPA1B | 1.17 | 9.20 | 12.35 | 3.21E-30 | 3.78E-29 | 57.55 |
| HSPB1 | 1.47 | 11.96 | 27.58 | 2.07E-97 | 1.38E-94 | 211.66 |
| HSPH1 | 1.02 | 8.54 | 18.98 | 6.31E-59 | 3.16E-57 | 123.35 |
| ID1 | -1.08 | 8.17 | -13.14 | 2.03E-33 | 2.79E-32 | 64.88 |
| ID2 | -1.09 | 9.76 | -10.56 | 2.35E-23 | 2.00E-22 | 41.86 |
| IDNK | -1.07 | 9.29 | -16.16 | 2.57E-46 | 6.76E-45 | 94.43 |
| IGF1 | -1.04 | 6.90 | -12.44 | 1.35E-30 | 1.61E-29 | 58.42 |
| IGF2BP2 | 1.52 | 7.75 | 15.66 | 4.09E-44 | 9.63E-43 | 89.38 |
| IGF2BP3 | 1.04 | 6.38 | 12.83 | 3.65E-32 | 4.70E-31 | 62.00 |
| IGFALS | -2.20 | 8.14 | -27.31 | 2.89E-96 | 1.69E-93 | 209.04 |
| IGFBP1 | -1.21 | 8.86 | -9.69 | 3.22E-20 | 2.36E-19 | 34.71 |
| IGFBP3 | -1.29 | 10.31 | -14.88 | 9.16E-41 | 1.82E-39 | 81.70 |
| IGLL1 | -1.45 | 10.13 | -8.07 | 7.03E-15 | 3.93E-14 | 22.56 |
| IL1RAP | -1.13 | 7.61 | -14.75 | 3.46E-40 | 6.67E-39 | 80.38 |
| ILF2 | 1.43 | 9.31 | 20.10 | 5.73E-64 | 3.85E-62 | 134.92 |
| IMPDH2 | 1.06 | 9.60 | 18.61 | 3.20E-57 | 1.45E-55 | 119.44 |
| INMT | -1.31 | 7.40 | -16.66 | 1.66E-48 | 4.82E-47 | 99.45 |
| IRAK1 | 1.23 | 8.38 | 26.27 | 1.08E-91 | 4.07E-89 | 198.54 |
| IRX3 | 1.11 | 7.12 | 10.07 | 1.39E-21 | 1.09E-20 | 37.82 |
| ITGA5 | 1.01 | 8.07 | 14.58 | 1.83E-39 | 3.40E-38 | 78.72 |
| KANSL3 | 1.03 | 9.46 | 15.62 | 5.73E-44 | 1.33E-42 | 89.05 |
| KBTBD11 | -1.70 | 7.29 | -22.13 | 3.56E-73 | 4.06E-71 | 156.05 |
| KCTD6 | 1.09 | 8.12 | 18.26 | 1.13E-55 | 4.73E-54 | 115.89 |
| KDM5B | 1.16 | 7.71 | 16.59 | 3.26E-48 | 9.31E-47 | 98.78 |
| KHK | -1.05 | 7.75 | -10.89 | 1.44E-24 | 1.30E-23 | 44.63 |
| KIAA0101 | 1.41 | 7.03 | 18.99 | 6.15E-59 | 3.08E-57 | 123.38 |
| KIAA0196 | 1.16 | 8.00 | 14.80 | 2.05E-40 | 4.00E-39 | 80.90 |
| KIFC1 | 1.18 | 6.76 | 23.17 | 7.36E-78 | 1.17E-75 | 166.80 |
| KLHL12 | 1.12 | 7.83 | 22.41 | 2.04E-74 | 2.50E-72 | 158.90 |
| KLKB1 | -1.69 | 9.82 | -18.43 | 1.98E-56 | 8.60E-55 | 117.63 |
| KMO | -1.52 | 8.18 | -22.58 | 3.40E-75 | 4.44E-73 | 160.69 |
| KPNB1 | 1.04 | 9.39 | 25.71 | 3.38E-89 | 1.12E-86 | 192.82 |
| KRTCAP2 | 1.18 | 11.49 | 21.04 | 3.11E-68 | 2.67E-66 | 144.71 |
| LAD1 | 1.07 | 9.30 | 9.32 | 5.84E-19 | 4.00E-18 | 31.84 |
| LAGE3 | 1.12 | 8.73 | 17.94 | 3.27E-54 | 1.27E-52 | 112.54 |
| LAMC1 | 1.79 | 8.94 | 24.85 | 2.07E-85 | 5.01E-83 | 184.13 |
| LAPTM4B | 1.57 | 9.22 | 15.61 | 6.61E-44 | 1.53E-42 | 88.90 |
| LASP1 | 1.10 | 10.62 | 20.53 | 6.46E-66 | 4.90E-64 | 139.39 |
| LCAT | -2.41 | 8.23 | -28.59 | 8.28E-102 | 8.71E-99 | 221.75 |
| LCN2 | 1.69 | 8.53 | 13.04 | 5.01E-33 | 6.76E-32 | 63.98 |
| LEAP2 | -1.08 | 11.78 | -11.35 | 2.66E-26 | 2.59E-25 | 48.59 |
| LECT2 | -1.92 | 10.33 | -13.74 | 6.56E-36 | 1.02E-34 | 70.58 |
| LINC01018 | -1.10 | 8.26 | -10.79 | 3.39E-24 | 3.00E-23 | 43.78 |
| LIPC | -1.03 | 10.00 | -10.93 | 9.72E-25 | 8.84E-24 | 45.02 |
| LMNA | 1.27 | 8.79 | 24.70 | 9.75E-85 | 2.25E-82 | 182.58 |
| LOXL4 | 1.29 | 7.49 | 10.10 | 1.12E-21 | 8.79E-21 | 38.04 |
| LRRC1 | 1.23 | 7.70 | 17.22 | 5.25E-51 | 1.71E-49 | 105.18 |
| LSM2 | 1.13 | 9.70 | 26.89 | 1.99E-94 | 1.00E-91 | 204.82 |
| LSM4 | 1.26 | 9.18 | 22.03 | 1.01E-72 | 1.12E-70 | 155.01 |
| LY6E | -1.96 | 10.72 | -18.45 | 1.58E-56 | 6.88E-55 | 117.85 |
| LYZ | 1.44 | 8.90 | 8.64 | 1.07E-16 | 6.54E-16 | 26.69 |
| MANBAL | 1.08 | 9.18 | 27.03 | 5.25E-95 | 2.76E-92 | 206.15 |
| MANF | 1.26 | 10.43 | 21.18 | 7.33E-69 | 6.46E-67 | 146.15 |
| MAPRE1 | 1.11 | 8.64 | 20.18 | 2.55E-64 | 1.75E-62 | 135.73 |
| MARCKS | 1.09 | 9.69 | 12.61 | 2.91E-31 | 3.59E-30 | 59.94 |
| MARCO | -1.18 | 7.14 | -18.90 | 1.50E-58 | 7.38E-57 | 122.49 |
| MASP2 | -1.17 | 8.18 | -15.21 | 3.53E-42 | 7.52E-41 | 84.94 |
| MAT1A | -1.34 | 10.13 | -14.62 | 1.23E-39 | 2.30E-38 | 79.12 |
| MBL2 | -1.74 | 9.74 | -14.51 | 3.50E-39 | 6.39E-38 | 78.08 |
| MCM2 | 1.19 | 6.93 | 18.90 | 1.56E-58 | 7.65E-57 | 122.45 |
| MCM3 | 1.45 | 9.27 | 26.31 | 7.62E-92 | 2.93E-89 | 198.89 |
| MCM4 | 1.25 | 7.49 | 19.40 | 8.27E-61 | 4.68E-59 | 127.67 |
| MCM5 | 1.02 | 7.93 | 20.62 | 2.61E-66 | 2.03E-64 | 140.29 |
| MCM6 | 1.32 | 8.47 | 22.99 | 4.98E-77 | 7.46E-75 | 164.89 |
| MCM7 | 1.43 | 8.85 | 25.20 | 5.69E-87 | 1.50E-84 | 187.71 |
| MCRS1 | 1.18 | 8.59 | 29.96 | 1.14E-107 | 1.74E-104 | 235.19 |
| MDK | 2.39 | 8.82 | 22.91 | 1.06E-76 | 1.56E-74 | 164.14 |
| MED20 | 1.14 | 8.61 | 27.06 | 3.65E-95 | 1.96E-92 | 206.51 |
| MELK | 1.08 | 6.99 | 20.56 | 4.49E-66 | 3.43E-64 | 139.75 |
| MEP1A | 1.16 | 6.50 | 10.57 | 2.20E-23 | 1.87E-22 | 41.93 |
| MFGE8 | 1.21 | 8.40 | 16.84 | 2.64E-49 | 7.93E-48 | 101.28 |
| MFSD10 | 1.08 | 8.25 | 15.63 | 5.22E-44 | 1.22E-42 | 89.14 |
| MID1IP1 | 1.30 | 7.40 | 20.70 | 1.12E-66 | 8.83E-65 | 141.14 |
| MMP9 | 1.22 | 6.81 | 12.27 | 6.70E-30 | 7.76E-29 | 56.82 |
| MNS1 | 1.06 | 6.89 | 12.60 | 3.12E-31 | 3.85E-30 | 59.87 |
| MPV17 | 1.07 | 7.39 | 22.74 | 6.68E-76 | 9.27E-74 | 162.31 |
| MPZL1 | 1.28 | 7.69 | 26.32 | 6.27E-92 | 2.47E-89 | 199.08 |
| MRGBP | 1.25 | 7.77 | 19.09 | 2.16E-59 | 1.11E-57 | 124.42 |
| MRPL24 | 1.03 | 10.55 | 23.93 | 2.92E-81 | 5.60E-79 | 174.61 |
| MT1A | -2.84 | 12.15 | -23.64 | 5.44E-80 | 9.44E-78 | 171.69 |
| MT1E | -3.28 | 11.35 | -22.80 | 3.55E-76 | 5.01E-74 | 162.94 |
| MT1F | -4.05 | 10.42 | -28.96 | 2.26E-103 | 2.61E-100 | 225.34 |
| MT1G | -4.31 | 11.65 | -26.06 | 8.80E-91 | 3.18E-88 | 196.45 |
| MT1H | -4.50 | 9.60 | -29.13 | 4.11E-104 | 5.17E-101 | 227.03 |
| MT1M | -3.38 | 8.35 | -24.39 | 2.45E-83 | 5.30E-81 | 179.37 |
| MT1X | -3.18 | 11.56 | -21.74 | 2.18E-71 | 2.25E-69 | 151.95 |
| MT2A | -2.44 | 12.25 | -21.85 | 6.52E-72 | 6.99E-70 | 153.15 |
| MTFP1 | 1.13 | 7.79 | 17.87 | 6.29E-54 | 2.41E-52 | 111.89 |
| MTMR11 | 1.05 | 7.09 | 13.60 | 2.60E-35 | 3.94E-34 | 69.21 |
| MUC13 | 1.46 | 7.54 | 11.78 | 5.79E-28 | 6.11E-27 | 52.39 |
| NABP2 | 1.05 | 7.70 | 24.37 | 2.88E-83 | 6.14E-81 | 179.21 |
| NAT2 | -2.24 | 7.80 | -29.39 | 3.02E-105 | 4.33E-102 | 229.63 |
| NAXE | 1.15 | 9.73 | 25.31 | 1.98E-87 | 5.52E-85 | 188.76 |
| NCAPD2 | 1.09 | 6.85 | 20.65 | 1.84E-66 | 1.44E-64 | 140.64 |
| NCAPG | 1.61 | 6.80 | 24.16 | 2.50E-82 | 5.11E-80 | 177.06 |
| NCSTN | 1.19 | 10.13 | 23.25 | 3.21E-78 | 5.24E-76 | 167.63 |
| NDRG2 | -1.04 | 9.03 | -17.42 | 6.69E-52 | 2.29E-50 | 107.24 |
| NDUFA4L2 | 1.15 | 6.75 | 16.51 | 7.53E-48 | 2.13E-46 | 97.94 |
| NEU1 | 1.39 | 8.52 | 26.07 | 8.32E-91 | 3.03E-88 | 196.51 |
| NME1 | 1.26 | 10.96 | 25.84 | 9.02E-90 | 3.07E-87 | 194.13 |
| NME1-NME2 | 1.08 | 10.11 | 17.11 | 1.64E-50 | 5.18E-49 | 104.05 |
| NNMT | -2.20 | 9.79 | -15.04 | 2.01E-41 | 4.15E-40 | 83.21 |
| NOCT | -1.02 | 8.04 | -15.72 | 2.08E-44 | 4.95E-43 | 90.06 |
| NOL7 | 1.03 | 9.73 | 18.75 | 6.96E-58 | 3.29E-56 | 120.96 |
| NOP56 | 1.21 | 9.20 | 22.22 | 1.42E-73 | 1.66E-71 | 156.96 |
| NPC1 | 1.12 | 7.46 | 20.01 | 1.40E-63 | 9.23E-62 | 134.03 |
| NPLOC4 | 1.02 | 7.44 | 25.42 | 6.59E-88 | 1.90E-85 | 189.86 |
| NPW | -1.42 | 9.68 | -8.76 | 4.55E-17 | 2.83E-16 | 27.54 |
| NQO1 | 1.61 | 8.51 | 12.77 | 6.34E-32 | 8.05E-31 | 61.45 |
| NREP | 1.22 | 8.35 | 12.93 | 1.38E-32 | 1.81E-31 | 62.97 |
| NSMCE2 | 1.19 | 7.50 | 19.57 | 1.49E-61 | 8.67E-60 | 129.38 |
| NT5DC2 | 1.06 | 6.65 | 14.79 | 2.32E-40 | 4.50E-39 | 80.78 |
| NUAK1 | 1.07 | 8.24 | 18.84 | 2.96E-58 | 1.43E-56 | 121.81 |
| NUP205 | 1.08 | 7.90 | 26.70 | 1.46E-93 | 6.52E-91 | 202.83 |
| NUP37 | 1.10 | 7.98 | 19.95 | 2.70E-63 | 1.76E-61 | 133.38 |
| NUP62 | 1.20 | 8.91 | 30.44 | 1.10E-109 | 2.08E-106 | 239.81 |
| NUPR1 | 1.15 | 9.24 | 13.44 | 1.17E-34 | 1.73E-33 | 67.71 |
| NUSAP1 | 1.81 | 7.56 | 27.99 | 3.16E-99 | 2.41E-96 | 215.83 |
| OAT | -1.04 | 8.43 | -10.77 | 4.05E-24 | 3.56E-23 | 43.61 |
| OGDHL | -1.09 | 8.35 | -14.05 | 3.31E-37 | 5.47E-36 | 73.55 |
| OIT3 | -1.10 | 6.38 | -20.52 | 7.01E-66 | 5.30E-64 | 139.31 |
| ORM2 | -1.05 | 12.88 | -10.93 | 9.77E-25 | 8.88E-24 | 45.02 |
| OTC | -1.28 | 10.54 | -10.05 | 1.75E-21 | 1.36E-20 | 37.60 |
| PA2G4 | 1.09 | 8.93 | 19.98 | 1.97E-63 | 1.29E-61 | 133.69 |
| PABPC1 | 1.13 | 11.39 | 17.89 | 5.27E-54 | 2.03E-52 | 112.06 |
| PACSIN2 | 1.00 | 9.11 | 19.72 | 3.10E-62 | 1.89E-60 | 130.95 |
| PAFAH1B3 | 1.74 | 8.06 | 20.24 | 1.33E-64 | 9.42E-63 | 136.38 |
| PAH | -1.04 | 10.39 | -9.88 | 6.99E-21 | 5.28E-20 | 36.22 |
| PALLD | 1.07 | 7.91 | 14.07 | 2.76E-37 | 4.59E-36 | 73.73 |
| PAMR1 | -1.28 | 6.86 | -25.43 | 5.96E-88 | 1.73E-85 | 189.96 |
| PANK1 | -1.41 | 9.89 | -20.13 | 4.01E-64 | 2.72E-62 | 135.28 |
| PARP1 | 1.32 | 9.72 | 30.50 | 6.25E-110 | 1.29E-106 | 240.38 |
| PARP12 | 1.10 | 8.30 | 19.10 | 1.95E-59 | 1.01E-57 | 124.52 |
| PCK1 | -2.56 | 9.23 | -22.22 | 1.35E-73 | 1.58E-71 | 157.02 |
| PDK4 | -1.87 | 9.74 | -17.54 | 1.95E-52 | 6.88E-51 | 108.47 |
| PDXK | 1.04 | 7.79 | 14.50 | 3.98E-39 | 7.23E-38 | 77.95 |
| PEA15 | 1.54 | 9.25 | 32.41 | 6.99E-118 | 4.13E-114 | 258.61 |
| PEG10 | 1.45 | 7.44 | 11.99 | 8.36E-29 | 9.18E-28 | 54.32 |
| PEMT | -1.09 | 10.31 | -15.82 | 7.79E-45 | 1.90E-43 | 91.03 |
| PGLYRP2 | -2.01 | 11.25 | -14.10 | 1.99E-37 | 3.33E-36 | 74.06 |
| PHGDH | -1.28 | 10.90 | -11.98 | 9.13E-29 | 1.00E-27 | 54.23 |
| PHLDA1 | -1.06 | 8.93 | -9.85 | 8.69E-21 | 6.54E-20 | 36.01 |
| PHPT1 | 1.11 | 9.10 | 21.16 | 8.81E-69 | 7.74E-67 | 145.97 |
| PIGC | 1.17 | 8.42 | 24.95 | 7.82E-86 | 1.98E-83 | 185.10 |
| PIGU | 1.06 | 8.38 | 20.03 | 1.18E-63 | 7.83E-62 | 134.20 |
| PIK3R2 | 1.39 | 9.33 | 17.74 | 2.54E-53 | 9.48E-52 | 110.49 |
| PIPOX | -1.00 | 9.74 | -9.73 | 2.21E-20 | 1.63E-19 | 35.08 |
| PITX1 | 1.13 | 6.41 | 12.21 | 1.08E-29 | 1.24E-28 | 56.35 |
| PKM | 1.28 | 7.30 | 15.02 | 2.41E-41 | 4.94E-40 | 83.03 |
| PLA2G16 | -1.11 | 8.02 | -15.18 | 4.97E-42 | 1.05E-40 | 84.60 |
| PLAC8 | -1.09 | 6.65 | -14.15 | 1.26E-37 | 2.13E-36 | 74.51 |
| PLCB1 | 1.09 | 6.82 | 17.49 | 3.12E-52 | 1.09E-50 | 108.00 |
| PLG | -1.53 | 11.86 | -13.01 | 6.70E-33 | 8.98E-32 | 63.69 |
| PLGLB1 | -1.67 | 10.60 | -15.77 | 1.30E-44 | 3.14E-43 | 90.52 |
| PLIN2 | -1.19 | 10.09 | -12.41 | 1.81E-30 | 2.15E-29 | 58.13 |
| PLOD3 | 1.29 | 9.29 | 26.11 | 5.31E-91 | 1.96E-88 | 196.96 |
| PLVAP | 1.15 | 7.38 | 24.90 | 1.30E-85 | 3.19E-83 | 184.59 |
| PNPLA7 | -1.08 | 8.24 | -16.79 | 4.22E-49 | 1.26E-47 | 100.81 |
| PODXL | 1.38 | 7.15 | 23.77 | 1.45E-80 | 2.64E-78 | 173.01 |
| POGK | 1.13 | 8.61 | 26.83 | 3.65E-94 | 1.76E-91 | 204.21 |
| POLR2G | 1.05 | 9.87 | 37.84 | 1.95E-139 | 9.23E-135 | 307.99 |
| POLR3C | 1.10 | 8.34 | 29.32 | 5.99E-105 | 8.10E-102 | 228.95 |
| PON1 | -1.37 | 9.29 | -12.87 | 2.61E-32 | 3.39E-31 | 62.34 |
| PON3 | -1.18 | 11.71 | -12.97 | 9.56E-33 | 1.27E-31 | 63.34 |
| PPARG | 1.02 | 8.30 | 17.24 | 4.10E-51 | 1.34E-49 | 105.43 |
| PPM1G | 1.05 | 9.90 | 24.42 | 1.80E-83 | 3.93E-81 | 179.68 |
| PRC1 | 1.91 | 7.29 | 27.60 | 1.59E-97 | 1.08E-94 | 211.92 |
| PRICKLE4 | 1.12 | 9.96 | 22.58 | 3.24E-75 | 4.26E-73 | 160.73 |
| PROZ | -1.24 | 7.64 | -14.87 | 1.04E-40 | 2.05E-39 | 81.58 |
| PRPF3 | 1.14 | 7.50 | 23.82 | 8.95E-81 | 1.64E-78 | 173.49 |
| PSMB4 | 1.10 | 10.58 | 26.47 | 1.41E-92 | 5.70E-90 | 200.57 |
| PSMD4 | 1.37 | 9.95 | 31.50 | 3.87E-114 | 1.31E-110 | 250.03 |
| PSME3 | 1.02 | 7.42 | 27.84 | 1.51E-98 | 1.08E-95 | 214.27 |
| PTCD1 | 1.07 | 8.34 | 25.36 | 1.11E-87 | 3.16E-85 | 189.34 |
| PTH1R | -1.32 | 6.84 | -23.68 | 3.58E-80 | 6.34E-78 | 172.11 |
| PTK2 | 1.16 | 9.69 | 26.53 | 7.51E-93 | 3.06E-90 | 201.20 |
| PTPRF | 1.01 | 8.29 | 21.33 | 1.49E-69 | 1.39E-67 | 147.74 |
| PTTG1 | 1.78 | 8.08 | 30.27 | 5.54E-109 | 9.04E-106 | 238.20 |
| PTTG3P | 1.45 | 6.91 | 23.20 | 5.34E-78 | 8.51E-76 | 167.12 |
| PUF60 | 1.20 | 10.58 | 23.69 | 3.35E-80 | 5.98E-78 | 172.17 |
| PYGB | 1.13 | 7.42 | 20.50 | 8.91E-66 | 6.69E-64 | 139.07 |
| PZP | -1.04 | 7.22 | -9.63 | 5.22E-20 | 3.78E-19 | 34.23 |
| RAB11FIP1 | 1.03 | 8.38 | 13.14 | 1.91E-33 | 2.63E-32 | 64.94 |
| RAB3IP | 1.18 | 7.24 | 20.86 | 1.96E-67 | 1.61E-65 | 142.87 |
| RACGAP1 | 1.19 | 6.74 | 21.83 | 8.65E-72 | 9.20E-70 | 152.87 |
| RALY | 1.02 | 8.66 | 21.74 | 2.05E-71 | 2.11E-69 | 152.01 |
| RAN | 1.11 | 10.02 | 13.24 | 7.97E-34 | 1.12E-32 | 65.81 |
| RANGAP1 | 1.04 | 9.14 | 15.78 | 1.24E-44 | 2.98E-43 | 90.57 |
| RAP2A | 1.21 | 7.70 | 18.82 | 3.31E-58 | 1.60E-56 | 121.70 |
| RBCK1 | 1.00 | 7.93 | 19.27 | 3.35E-60 | 1.83E-58 | 126.28 |
| RBM34 | 1.00 | 7.50 | 14.81 | 1.88E-40 | 3.67E-39 | 80.99 |
| RBM42 | 1.03 | 8.79 | 22.33 | 4.62E-74 | 5.57E-72 | 158.08 |
| RBP4 | -1.02 | 12.12 | -10.80 | 3.06E-24 | 2.70E-23 | 43.89 |
| RCAN1 | -1.45 | 9.03 | -19.05 | 3.02E-59 | 1.54E-57 | 124.09 |
| RCC2 | 1.06 | 8.27 | 19.82 | 1.08E-62 | 6.75E-61 | 132.00 |
| RCL1 | -1.22 | 7.95 | -17.10 | 1.75E-50 | 5.54E-49 | 103.98 |
| RDH16 | -2.57 | 10.86 | -18.08 | 7.23E-55 | 2.91E-53 | 114.04 |
| RDH5 | -2.06 | 9.16 | -22.49 | 8.85E-75 | 1.12E-72 | 159.73 |
| REG3A | 1.43 | 7.30 | 8.12 | 4.72E-15 | 2.66E-14 | 22.95 |
| REPIN1 | 1.20 | 9.38 | 24.54 | 5.25E-84 | 1.18E-81 | 180.91 |
| RFC4 | 1.18 | 7.33 | 19.98 | 1.88E-63 | 1.24E-61 | 133.74 |
| RFWD2 | 1.03 | 9.35 | 27.95 | 5.13E-99 | 3.79E-96 | 215.34 |
| RFX5 | 1.42 | 7.81 | 24.06 | 7.18E-82 | 1.42E-79 | 176.00 |
| RFXANK | 1.10 | 7.70 | 24.32 | 5.05E-83 | 1.07E-80 | 178.65 |
| RGCC | 1.10 | 8.10 | 13.30 | 4.15E-34 | 5.92E-33 | 66.45 |
| RGN | -1.03 | 9.05 | -11.22 | 7.80E-26 | 7.43E-25 | 47.53 |
| RGS5 | 1.02 | 6.96 | 16.37 | 3.04E-47 | 8.35E-46 | 96.55 |
| RHOC | 1.04 | 10.32 | 21.23 | 4.21E-69 | 3.80E-67 | 146.70 |
| RIDA | -1.35 | 10.95 | -12.07 | 3.90E-29 | 4.35E-28 | 55.07 |
| RNASE4 | -1.63 | 10.51 | -11.21 | 9.02E-26 | 8.58E-25 | 47.38 |
| RND3 | -1.59 | 8.40 | -21.08 | 2.09E-68 | 1.80E-66 | 145.11 |
| RNF115 | 1.03 | 8.98 | 19.95 | 2.84E-63 | 1.85E-61 | 133.33 |
| RNF34 | 1.13 | 8.12 | 25.69 | 4.17E-89 | 1.37E-86 | 192.61 |
| RPL15 | 1.34 | 9.17 | 15.74 | 1.73E-44 | 4.15E-43 | 90.24 |
| RPS21 | 1.01 | 8.92 | 13.77 | 4.95E-36 | 7.73E-35 | 70.86 |
| RRAGD | 1.45 | 8.82 | 18.74 | 8.20E-58 | 3.86E-56 | 120.80 |
| RUSC1 | 1.18 | 8.34 | 20.18 | 2.42E-64 | 1.67E-62 | 135.78 |
| S100A10 | 1.50 | 10.27 | 21.63 | 6.96E-71 | 6.97E-69 | 150.79 |
| S100P | 1.96 | 6.98 | 11.78 | 5.67E-28 | 5.98E-27 | 52.42 |
| SAA1 | -1.01 | 9.32 | -5.33 | 1.57E-07 | 5.80E-07 | 5.99 |
| SAA4 | -1.86 | 12.23 | -13.59 | 2.65E-35 | 4.02E-34 | 69.19 |
| SAC3D1 | 1.24 | 8.63 | 21.45 | 4.41E-70 | 4.24E-68 | 148.95 |
| SAE1 | 1.38 | 10.15 | 34.80 | 1.35E-127 | 3.20E-123 | 280.87 |
| SCAMP3 | 1.54 | 9.34 | 28.05 | 1.80E-99 | 1.42E-96 | 216.39 |
| SCP2 | -1.00 | 8.30 | -9.85 | 8.57E-21 | 6.45E-20 | 36.02 |
| SCRIB | 1.21 | 7.93 | 16.98 | 6.15E-50 | 1.90E-48 | 102.73 |
| SDS | -2.00 | 9.51 | -13.39 | 1.90E-34 | 2.76E-33 | 67.23 |
| SELM | 1.02 | 8.13 | 11.57 | 3.67E-27 | 3.73E-26 | 50.56 |
| SEPN1 | 1.01 | 7.65 | 17.97 | 2.41E-54 | 9.51E-53 | 112.84 |
| 9-Sep | 1.08 | 10.83 | 21.75 | 1.89E-71 | 1.96E-69 | 152.09 |
| SERPINA11 | -1.91 | 10.25 | -17.84 | 9.24E-54 | 3.53E-52 | 111.50 |
| SERPINE2 | 1.16 | 8.88 | 10.54 | 2.88E-23 | 2.44E-22 | 41.66 |
| SEZ6L2 | 1.26 | 6.85 | 9.75 | 1.98E-20 | 1.46E-19 | 35.19 |
| SF3A2 | 1.10 | 8.90 | 17.72 | 3.14E-53 | 1.17E-51 | 110.28 |
| SF3B4 | 1.75 | 9.26 | 31.40 | 1.07E-113 | 3.16E-110 | 249.01 |
| SGCE | 1.01 | 8.73 | 10.24 | 3.44E-22 | 2.77E-21 | 39.21 |
| SHBG | -1.68 | 9.44 | -14.83 | 1.61E-40 | 3.15E-39 | 81.14 |
| SHC1 | 1.39 | 9.91 | 24.97 | 6.10E-86 | 1.56E-83 | 185.35 |
| SHMT1 | -1.19 | 10.51 | -12.12 | 2.52E-29 | 2.84E-28 | 55.51 |
| SIPA1L2 | 1.38 | 7.48 | 14.64 | 1.06E-39 | 2.00E-38 | 79.26 |
| SKAP1 | -1.44 | 8.09 | -17.08 | 2.31E-50 | 7.24E-49 | 103.71 |
| SLC10A1 | -2.25 | 10.64 | -14.15 | 1.26E-37 | 2.12E-36 | 74.51 |
| SLC12A9 | 1.01 | 8.12 | 20.10 | 5.41E-64 | 3.64E-62 | 134.98 |
| SLC13A5 | -1.05 | 10.47 | -7.34 | 1.05E-12 | 5.24E-12 | 17.63 |
| SLC22A1 | -2.27 | 9.08 | -20.11 | 5.15E-64 | 3.48E-62 | 135.03 |
| SLC22A10 | -1.04 | 7.54 | -14.62 | 1.19E-39 | 2.23E-38 | 79.15 |
| SLC25A39 | 1.28 | 10.31 | 27.43 | 9.07E-97 | 5.65E-94 | 210.19 |
| SLC25A47 | -1.75 | 8.22 | -23.85 | 6.30E-81 | 1.17E-78 | 173.84 |
| SLC26A6 | 1.25 | 7.66 | 24.58 | 3.50E-84 | 7.97E-82 | 181.31 |
| SLC27A2 | -1.26 | 10.06 | -11.16 | 1.40E-25 | 1.33E-24 | 46.94 |
| SLC27A5 | -1.88 | 10.21 | -15.43 | 4.11E-43 | 9.21E-42 | 87.08 |
| SLC38A4 | -1.03 | 9.11 | -10.17 | 6.16E-22 | 4.91E-21 | 38.63 |
| SLC39A1 | 1.26 | 8.98 | 26.80 | 5.11E-94 | 2.39E-91 | 203.88 |
| SLC39A5 | -1.02 | 9.52 | -8.64 | 1.07E-16 | 6.56E-16 | 26.69 |
| SLC41A3 | 1.12 | 8.15 | 24.74 | 6.87E-85 | 1.59E-82 | 182.93 |
| SLC50A1 | 1.11 | 8.44 | 20.25 | 1.13E-64 | 7.97E-63 | 136.54 |
| SLC52A2 | 1.22 | 7.50 | 21.16 | 9.10E-69 | 7.96E-67 | 145.93 |
| SLC7A2 | -1.35 | 9.97 | -13.57 | 3.38E-35 | 5.11E-34 | 68.95 |
| SLCO1B3 | -2.27 | 7.78 | -21.35 | 1.24E-69 | 1.16E-67 | 147.92 |
| SMAD5 | 1.13 | 8.89 | 21.22 | 4.79E-69 | 4.29E-67 | 146.57 |
| SMARCC1 | 1.06 | 8.38 | 20.04 | 1.01E-63 | 6.74E-62 | 134.35 |
| SMARCD1 | 1.11 | 7.98 | 25.46 | 4.38E-88 | 1.30E-85 | 190.26 |
| SMC4 | 1.08 | 7.52 | 19.10 | 1.83E-59 | 9.46E-58 | 124.59 |
| SMG5 | 1.31 | 7.61 | 22.63 | 1.92E-75 | 2.58E-73 | 161.25 |
| SMG7 | 1.16 | 8.18 | 25.56 | 1.50E-88 | 4.68E-86 | 191.33 |
| SMIM24 | -1.35 | 8.96 | -12.80 | 4.92E-32 | 6.28E-31 | 61.71 |
| SMYD3 | 1.23 | 7.74 | 17.55 | 1.83E-52 | 6.48E-51 | 108.53 |
| SNAR-A1 | 1.08 | 7.44 | 8.30 | 1.35E-15 | 7.84E-15 | 24.19 |
| SNHG6 | 1.22 | 9.16 | 17.43 | 6.22E-52 | 2.13E-50 | 107.31 |
| SNORD13 | -1.37 | 9.03 | -13.14 | 2.00E-33 | 2.75E-32 | 64.89 |
| SNRNP70 | 1.12 | 8.49 | 21.85 | 6.98E-72 | 7.48E-70 | 153.08 |
| SNRPB | 1.45 | 9.91 | 27.31 | 2.87E-96 | 1.69E-93 | 209.04 |
| SNRPC | 1.14 | 9.17 | 28.14 | 7.39E-100 | 6.25E-97 | 217.27 |
| SNX27 | 1.06 | 8.49 | 25.44 | 5.04E-88 | 1.48E-85 | 190.13 |
| SOCS2 | -1.86 | 9.23 | -18.78 | 5.14E-58 | 2.45E-56 | 121.26 |
| SPC24 | 1.17 | 7.45 | 18.93 | 1.10E-58 | 5.43E-57 | 122.80 |
| SPINK1 | 3.86 | 9.32 | 17.11 | 1.54E-50 | 4.88E-49 | 104.11 |
| SPNS1 | 1.06 | 8.34 | 19.32 | 1.99E-60 | 1.10E-58 | 126.80 |
| SPP1 | 1.60 | 8.87 | 10.02 | 2.18E-21 | 1.69E-20 | 37.37 |
| SPP2 | -1.97 | 10.59 | -14.91 | 6.97E-41 | 1.40E-39 | 81.97 |
| SQLE | 2.02 | 9.01 | 22.28 | 7.94E-74 | 9.44E-72 | 157.54 |
| SQSTM1 | 1.03 | 12.55 | 17.48 | 3.75E-52 | 1.31E-50 | 107.81 |
| SRD5A2 | -1.73 | 7.43 | -25.12 | 1.31E-86 | 3.39E-84 | 186.88 |
| SRXN1 | 1.28 | 8.07 | 18.47 | 1.32E-56 | 5.80E-55 | 118.03 |
| SSR2 | 1.23 | 8.98 | 15.74 | 1.83E-44 | 4.37E-43 | 90.18 |
| ST3GAL6 | -1.19 | 7.87 | -19.04 | 3.55E-59 | 1.80E-57 | 123.93 |
| STEAP3 | -1.13 | 8.54 | -15.83 | 7.36E-45 | 1.80E-43 | 91.09 |
| STIP1 | 1.22 | 8.92 | 25.84 | 8.55E-90 | 2.93E-87 | 194.19 |
| STK24 | 1.21 | 9.60 | 22.16 | 2.60E-73 | 2.99E-71 | 156.36 |
| STT3A | 1.03 | 8.70 | 17.13 | 1.33E-50 | 4.25E-49 | 104.26 |
| STXBP6 | 1.12 | 7.04 | 15.28 | 1.82E-42 | 3.94E-41 | 85.60 |
| SUGP2 | 1.11 | 7.82 | 16.70 | 1.12E-48 | 3.26E-47 | 99.85 |
| SULT2A1 | -1.28 | 11.64 | -9.88 | 6.90E-21 | 5.22E-20 | 36.23 |
| TAGLN2 | 1.37 | 9.90 | 19.83 | 9.14E-63 | 5.76E-61 | 132.16 |
| TAT | -2.84 | 10.57 | -15.85 | 5.76E-45 | 1.42E-43 | 91.33 |
| TBCE | 1.35 | 8.15 | 22.56 | 4.08E-75 | 5.31E-73 | 160.50 |
| TCEAL9 | 1.25 | 8.57 | 14.68 | 7.13E-40 | 1.35E-38 | 79.66 |
| TCEB1 | 1.09 | 10.05 | 20.66 | 1.66E-66 | 1.30E-64 | 140.75 |
| TDO2 | -1.85 | 10.95 | -13.43 | 1.23E-34 | 1.81E-33 | 67.66 |
| TEAD2 | 1.30 | 8.21 | 19.66 | 5.76E-62 | 3.46E-60 | 130.33 |
| TESC | 1.06 | 7.39 | 8.92 | 1.28E-17 | 8.22E-17 | 28.79 |
| TFRC | 1.55 | 9.49 | 16.51 | 7.48E-48 | 2.11E-46 | 97.95 |
| THRSP | -2.18 | 8.84 | -17.54 | 1.96E-52 | 6.91E-51 | 108.46 |
| THY1 | 2.00 | 7.48 | 27.87 | 1.04E-98 | 7.57E-96 | 214.64 |
| TK1 | 1.07 | 7.19 | 19.12 | 1.47E-59 | 7.69E-58 | 124.81 |
| TKT | 1.62 | 9.65 | 20.44 | 1.63E-65 | 1.21E-63 | 138.47 |
| TLCD1 | 1.12 | 7.15 | 21.27 | 2.87E-69 | 2.62E-67 | 147.08 |
| TMCO3 | 1.06 | 7.14 | 15.19 | 4.29E-42 | 9.12E-41 | 84.75 |
| TMED3 | 1.11 | 9.48 | 10.00 | 2.44E-21 | 1.88E-20 | 37.26 |
| TMEM106C | 1.32 | 8.96 | 20.67 | 1.55E-66 | 1.21E-64 | 140.82 |
| TMEM147 | 1.08 | 9.43 | 19.40 | 8.56E-61 | 4.83E-59 | 127.64 |
| TMEM27 | -1.38 | 7.49 | -21.75 | 1.91E-71 | 1.98E-69 | 152.08 |
| TMEM45B | 1.55 | 7.75 | 13.57 | 3.43E-35 | 5.17E-34 | 68.93 |
| TMEM9 | 1.14 | 8.16 | 23.00 | 4.12E-77 | 6.18E-75 | 165.08 |
| TMSB10 | 1.06 | 11.63 | 14.04 | 3.49E-37 | 5.75E-36 | 73.50 |
| TOMM40 | 1.43 | 9.00 | 28.15 | 6.90E-100 | 5.94E-97 | 217.34 |
| TOMM40L | 1.49 | 7.47 | 27.45 | 7.05E-97 | 4.51E-94 | 210.44 |
| TOMM6 | 1.02 | 10.09 | 20.79 | 4.20E-67 | 3.39E-65 | 142.12 |
| TOP2A | 2.54 | 7.48 | 27.72 | 4.68E-98 | 3.25E-95 | 213.14 |
| TP53BP2 | 1.04 | 7.66 | 17.39 | 8.77E-52 | 2.97E-50 | 106.97 |
| TP53I3 | 1.19 | 7.20 | 17.77 | 1.88E-53 | 7.05E-52 | 110.79 |
| TPD52L2 | 1.11 | 9.25 | 19.43 | 6.04E-61 | 3.43E-59 | 127.99 |
| TPM2 | 1.25 | 7.73 | 15.79 | 1.12E-44 | 2.69E-43 | 90.67 |
| TPR | 1.10 | 7.97 | 24.74 | 6.49E-85 | 1.52E-82 | 182.99 |
| TRIM24 | 1.11 | 7.80 | 20.34 | 4.86E-65 | 3.50E-63 | 137.38 |
| TRNP1 | 1.07 | 7.60 | 12.15 | 1.96E-29 | 2.22E-28 | 55.76 |
| TRRAP | 1.07 | 8.29 | 21.55 | 1.47E-70 | 1.45E-68 | 150.05 |
| TTC13 | 1.06 | 7.07 | 22.30 | 5.94E-74 | 7.09E-72 | 157.83 |
| TTC36 | -1.14 | 6.82 | -25.26 | 3.13E-87 | 8.60E-85 | 188.31 |
| TTR | -1.22 | 13.75 | -11.94 | 1.26E-28 | 1.38E-27 | 53.91 |
| TUBA1A | 1.45 | 11.65 | 25.93 | 3.53E-90 | 1.25E-87 | 195.07 |
| TUBA1B | 1.41 | 11.04 | 25.56 | 1.55E-88 | 4.79E-86 | 191.30 |
| TUBA1C | 1.43 | 10.40 | 21.22 | 4.53E-69 | 4.07E-67 | 146.63 |
| TUBB | 1.23 | 9.79 | 20.31 | 6.45E-65 | 4.61E-63 | 137.10 |
| TUBB2A | 1.04 | 8.35 | 14.05 | 3.10E-37 | 5.12E-36 | 73.62 |
| TUBB4B | 1.12 | 9.15 | 20.94 | 8.67E-68 | 7.27E-66 | 143.69 |
| TUBG1 | 1.23 | 8.68 | 25.22 | 5.11E-87 | 1.37E-84 | 187.82 |
| TUFT1 | 1.12 | 7.29 | 20.38 | 3.12E-65 | 2.29E-63 | 137.82 |
| TUG1 | 1.13 | 9.85 | 17.26 | 3.59E-51 | 1.17E-49 | 105.56 |
| TXNRD1 | 1.31 | 8.09 | 16.06 | 6.96E-46 | 1.79E-44 | 93.44 |
| UBD | 2.12 | 9.90 | 17.13 | 1.36E-50 | 4.34E-49 | 104.23 |
| UBE2C | 2.34 | 7.38 | 27.06 | 3.75E-95 | 1.99E-92 | 206.48 |
| UBE2Q2 | 1.03 | 7.99 | 13.23 | 8.54E-34 | 1.19E-32 | 65.74 |
| UBE2Z | 1.02 | 7.66 | 23.43 | 5.15E-79 | 8.63E-77 | 169.45 |
| UBQLN4 | 1.15 | 8.98 | 15.99 | 1.41E-45 | 3.57E-44 | 92.73 |
| UCK2 | 1.05 | 6.81 | 21.50 | 2.53E-70 | 2.47E-68 | 149.50 |
| UGP2 | -1.17 | 9.07 | -16.64 | 2.02E-48 | 5.83E-47 | 99.26 |
| UGT2B10 | -2.11 | 11.11 | -15.64 | 5.09E-44 | 1.19E-42 | 89.16 |
| UGT2B11 | -1.25 | 11.17 | -8.97 | 9.11E-18 | 5.89E-17 | 29.12 |
| UPB1 | -1.03 | 10.46 | -8.51 | 2.93E-16 | 1.75E-15 | 25.70 |
| UQCC2 | 1.16 | 9.16 | 26.59 | 4.18E-93 | 1.75E-90 | 201.78 |
| VARS | 1.22 | 8.64 | 22.97 | 5.67E-77 | 8.44E-75 | 164.76 |
| VAT1 | 1.04 | 9.12 | 17.35 | 1.32E-51 | 4.41E-50 | 106.56 |
| VIPR1 | -1.29 | 7.59 | -28.14 | 7.66E-100 | 6.36E-97 | 217.24 |
| VNN2 | 1.07 | 7.05 | 11.27 | 5.23E-26 | 5.02E-25 | 47.92 |
| VPS28 | 1.10 | 9.66 | 21.59 | 9.84E-71 | 9.78E-69 | 150.45 |
| VPS37C | 1.03 | 8.83 | 21.62 | 7.38E-71 | 7.36E-69 | 150.73 |
| VWF | 1.39 | 9.22 | 15.64 | 4.64E-44 | 1.09E-42 | 89.26 |
| WDR72 | -1.26 | 9.23 | -10.90 | 1.26E-24 | 1.14E-23 | 44.77 |
| WFS1 | 1.05 | 8.15 | 16.84 | 2.61E-49 | 7.86E-48 | 101.29 |
| XPR1 | 1.02 | 7.91 | 25.51 | 2.44E-88 | 7.34E-86 | 190.85 |
| YWHAZ | 1.34 | 10.61 | 20.17 | 2.80E-64 | 1.92E-62 | 135.64 |
| YY1AP1 | 1.05 | 8.97 | 18.55 | 5.55E-57 | 2.48E-55 | 118.89 |
| ZC3H3 | 1.05 | 7.79 | 19.63 | 7.96E-62 | 4.73E-60 | 130.00 |
| ZG16 | -1.45 | 7.95 | -18.70 | 1.23E-57 | 5.72E-56 | 120.39 |
| ZGPAT | -1.71 | 9.99 | -21.63 | 6.39E-71 | 6.44E-69 | 150.88 |
| ZNF207 | 1.13 | 8.67 | 33.71 | 3.20E-123 | 3.03E-119 | 270.84 |
| ZNF544 | 1.02 | 8.25 | 21.90 | 3.82E-72 | 4.15E-70 | 153.69 |
| ZNF581 | 1.04 | 7.97 | 18.78 | 5.48E-58 | 2.60E-56 | 121.20 |
| ZYX | 1.15 | 8.61 | 24.27 | 8.03E-83 | 1.67E-80 | 178.19 |

Table 2: List of DEGs for GSE39791 datasets

| **Symbol** | **logFC** | **AveExpr** | **t** | **P.Value** | **adj.P.Val** | **B** |
| --- | --- | --- | --- | --- | --- | --- |
| A2M | -1.34 | 13.22 | -8.76 | 4.89E-15 | 2.67E-13 | 23.65 |
| AADAT | -1.77 | 8.38 | -15.53 | 1.44E-32 | 3.41E-29 | 63.35 |
| ABAT | -1.80 | 10.03 | -12.91 | 8.20E-26 | 4.84E-23 | 48.06 |
| ABCA8 | -1.11 | 7.57 | -13.90 | 2.23E-28 | 2.29E-25 | 53.87 |
| ACAA1 | -1.03 | 9.90 | -7.36 | 1.30E-11 | 3.39E-10 | 15.92 |
| ACACB | -1.52 | 10.09 | -10.83 | 2.25E-20 | 4.32E-18 | 35.74 |
| ACAD11 | -1.08 | 9.83 | -9.53 | 5.41E-17 | 4.59E-15 | 28.08 |
| ACADS | -1.02 | 8.30 | -10.55 | 1.28E-19 | 2.11E-17 | 34.03 |
| ACSL1 | -1.44 | 11.54 | -8.14 | 1.67E-13 | 6.50E-12 | 20.18 |
| ACSL4 | 2.20 | 8.55 | 10.29 | 5.77E-19 | 7.91E-17 | 32.55 |
| ACSM3 | -1.48 | 8.36 | -12.16 | 7.81E-24 | 3.21E-21 | 43.58 |
| ACSM5 | -1.54 | 8.80 | -10.74 | 3.94E-20 | 7.30E-18 | 35.19 |
| ACTB | 1.47 | 10.57 | 5.94 | 2.00E-08 | 2.62E-07 | 8.76 |
| ADAMTSL2 | -1.54 | 8.87 | -10.29 | 6.03E-19 | 8.23E-17 | 32.50 |
| ADGRG7 | -1.72 | 8.24 | -18.02 | 1.03E-38 | 6.08E-35 | 77.22 |
| ADH1A | -1.38 | 13.69 | -6.57 | 8.42E-10 | 1.48E-08 | 11.84 |
| ADH1B | -2.59 | 10.11 | -13.14 | 2.10E-26 | 1.41E-23 | 49.40 |
| ADH1C | -2.01 | 11.44 | -6.88 | 1.72E-10 | 3.55E-09 | 13.39 |
| ADH4 | -3.45 | 11.72 | -12.51 | 9.04E-25 | 4.36E-22 | 45.70 |
| ADI1 | -1.43 | 10.66 | -10.71 | 4.64E-20 | 8.33E-18 | 35.03 |
| ADIRF | -1.41 | 9.75 | -6.76 | 3.24E-10 | 6.27E-09 | 12.78 |
| AFM | -2.19 | 12.11 | -9.43 | 9.63E-17 | 7.82E-15 | 27.51 |
| AGL | -1.35 | 9.90 | -11.48 | 4.74E-22 | 1.33E-19 | 39.54 |
| AGXT | -1.42 | 12.79 | -6.42 | 1.83E-09 | 2.99E-08 | 11.09 |
| AGXT2 | -1.22 | 8.24 | -10.82 | 2.51E-20 | 4.80E-18 | 35.63 |
| AHSG | -1.13 | 12.99 | -4.52 | 1.26E-05 | 8.96E-05 | 2.54 |
| AKR1B10 | 2.89 | 8.81 | 9.11 | 6.38E-16 | 4.26E-14 | 25.65 |
| AKR1B15 | 2.15 | 8.07 | 8.43 | 3.31E-14 | 1.49E-12 | 21.77 |
| AKR1C3 | 1.61 | 11.76 | 10.32 | 4.93E-19 | 6.93E-17 | 32.70 |
| AKR1C4 | -1.37 | 10.70 | -7.13 | 4.50E-11 | 1.05E-09 | 14.70 |
| AKR1D1 | -1.19 | 8.86 | -5.01 | 1.58E-06 | 1.37E-05 | 4.53 |
| AKR7A3 | -1.90 | 10.86 | -9.76 | 1.41E-17 | 1.40E-15 | 29.40 |
| ALDH1B1 | -1.32 | 9.37 | -11.46 | 5.24E-22 | 1.43E-19 | 39.44 |
| ALDH1L1 | -1.43 | 10.33 | -6.81 | 2.41E-10 | 4.81E-09 | 13.07 |
| ALDH2 | -1.43 | 11.27 | -7.97 | 4.47E-13 | 1.59E-11 | 19.22 |
| ALDH4A1 | -1.18 | 8.76 | -11.59 | 2.35E-22 | 7.02E-20 | 40.23 |
| ALDH5A1 | -1.20 | 10.46 | -9.84 | 8.86E-18 | 9.18E-16 | 29.86 |
| ALDH6A1 | -1.56 | 10.84 | -9.20 | 3.87E-16 | 2.71E-14 | 26.15 |
| ALDH8A1 | -1.68 | 9.33 | -13.64 | 1.04E-27 | 9.66E-25 | 52.36 |
| ALDOB | -1.31 | 13.29 | -5.35 | 3.31E-07 | 3.33E-06 | 6.04 |
| ALG1L | 1.05 | 7.33 | 8.29 | 7.28E-14 | 3.07E-12 | 21.00 |
| ALPL | -1.46 | 8.99 | -11.33 | 1.11E-21 | 2.76E-19 | 38.70 |
| AMBP | -1.00 | 9.75 | -6.13 | 7.86E-09 | 1.12E-07 | 9.67 |
| AMDHD1 | -1.08 | 9.88 | -6.42 | 1.82E-09 | 2.98E-08 | 11.09 |
| ANG | -1.38 | 12.90 | -7.11 | 4.97E-11 | 1.15E-09 | 14.61 |
| ANGPTL4 | -1.15 | 10.20 | -5.39 | 2.84E-07 | 2.89E-06 | 6.19 |
| ANXA10 | -1.49 | 8.74 | -9.31 | 2.04E-16 | 1.55E-14 | 26.77 |
| ANXA2 | 1.56 | 10.17 | 9.94 | 4.75E-18 | 5.33E-16 | 30.47 |
| AOX1 | -1.48 | 10.18 | -5.82 | 3.74E-08 | 4.58E-07 | 8.15 |
| APCS | -1.26 | 12.13 | -5.65 | 8.39E-08 | 9.54E-07 | 7.37 |
| APOA5 | -1.81 | 9.57 | -9.67 | 2.37E-17 | 2.25E-15 | 28.89 |
| APOC3 | -1.19 | 13.92 | -5.30 | 4.33E-07 | 4.23E-06 | 5.78 |
| APOC4 | -1.77 | 10.91 | -8.07 | 2.53E-13 | 9.45E-12 | 19.78 |
| APOF | -3.18 | 10.15 | -17.03 | 2.65E-36 | 9.63E-33 | 71.79 |
| AQP12A | -1.42 | 9.28 | -10.76 | 3.56E-20 | 6.62E-18 | 35.29 |
| AQP9 | -1.64 | 11.44 | -6.84 | 2.05E-10 | 4.17E-09 | 13.22 |
| ARG1 | -1.26 | 11.64 | -6.27 | 3.96E-09 | 6.03E-08 | 10.33 |
| ARPC3 | 1.18 | 8.40 | 8.91 | 2.01E-15 | 1.19E-13 | 24.53 |
| ASGR2 | -1.17 | 10.67 | -7.62 | 3.04E-12 | 9.05E-11 | 17.34 |
| ASPDH | -1.83 | 8.50 | -14.33 | 1.68E-29 | 2.15E-26 | 56.41 |
| ASPG | -1.52 | 8.13 | -13.03 | 4.00E-26 | 2.45E-23 | 48.77 |
| ASPM | 1.42 | 7.88 | 13.51 | 2.27E-27 | 1.88E-24 | 51.59 |
| ASS1 | -2.10 | 12.44 | -13.53 | 1.97E-27 | 1.69E-24 | 51.73 |
| ATF5 | -1.41 | 11.20 | -6.16 | 6.76E-09 | 9.78E-08 | 9.81 |
| AVPI1 | -1.15 | 9.76 | -9.43 | 9.96E-17 | 8.04E-15 | 27.48 |
| AZGP1 | -1.93 | 10.62 | -11.70 | 1.26E-22 | 4.03E-20 | 40.84 |
| BBOX1 | -1.84 | 8.45 | -10.16 | 1.30E-18 | 1.63E-16 | 31.75 |
| BCHE | -1.92 | 10.07 | -9.15 | 5.11E-16 | 3.48E-14 | 25.87 |
| BGN | -1.18 | 12.16 | -5.59 | 1.10E-07 | 1.22E-06 | 7.10 |
| BHMT | -1.99 | 11.11 | -8.61 | 1.20E-14 | 5.99E-13 | 22.77 |
| BLVRB | -1.11 | 9.83 | -9.88 | 6.65E-18 | 7.18E-16 | 30.14 |
| BSG | 1.00 | 9.47 | 8.53 | 1.86E-14 | 8.91E-13 | 22.34 |
| C1QTNF1 | -1.26 | 8.77 | -8.51 | 2.08E-14 | 9.77E-13 | 22.23 |
| C1RL | -1.03 | 8.80 | -10.16 | 1.29E-18 | 1.62E-16 | 31.75 |
| C20orf24 | 1.01 | 9.35 | 6.99 | 9.63E-11 | 2.08E-09 | 13.96 |
| C6 | -1.63 | 11.24 | -7.47 | 7.12E-12 | 1.96E-10 | 16.51 |
| C6orf48 | 1.13 | 9.61 | 11.02 | 7.45E-21 | 1.56E-18 | 36.83 |
| C7 | -1.82 | 9.26 | -7.47 | 7.24E-12 | 1.99E-10 | 16.49 |
| C8A | -2.06 | 11.57 | -9.80 | 1.07E-17 | 1.09E-15 | 29.67 |
| C8B | -1.10 | 10.52 | -6.35 | 2.62E-09 | 4.14E-08 | 10.74 |
| C9 | -3.55 | 10.97 | -13.63 | 1.08E-27 | 9.81E-25 | 52.32 |
| CA2 | -1.15 | 9.28 | -5.65 | 8.24E-08 | 9.37E-07 | 7.39 |
| CA5A | -1.23 | 8.68 | -9.62 | 3.16E-17 | 2.89E-15 | 28.61 |
| CAMK2B | -1.12 | 7.58 | -11.69 | 1.32E-22 | 4.14E-20 | 40.79 |
| CAP2 | 1.76 | 8.43 | 12.18 | 6.68E-24 | 2.77E-21 | 43.73 |
| CAT | -1.39 | 11.83 | -9.02 | 1.09E-15 | 6.91E-14 | 25.13 |
| CBS | -1.28 | 11.02 | -7.60 | 3.45E-12 | 1.02E-10 | 17.22 |
| CCL16 | -1.40 | 9.97 | -7.33 | 1.53E-11 | 3.91E-10 | 15.76 |
| CCL20 | 1.66 | 7.90 | 7.92 | 5.78E-13 | 2.00E-11 | 18.97 |
| CCT3 | 1.18 | 8.66 | 13.17 | 1.74E-26 | 1.23E-23 | 49.59 |
| CD14 | -1.05 | 13.02 | -7.30 | 1.75E-11 | 4.43E-10 | 15.63 |
| CD24 | 1.39 | 9.37 | 5.66 | 8.05E-08 | 9.18E-07 | 7.41 |
| CD34 | 1.11 | 7.92 | 11.38 | 8.42E-22 | 2.16E-19 | 38.97 |
| CDC20 | 1.58 | 7.61 | 11.81 | 6.11E-23 | 2.21E-20 | 41.55 |
| CDHR2 | -1.32 | 7.66 | -15.32 | 5.05E-32 | 1.04E-28 | 62.12 |
| CES1 | -1.35 | 13.05 | -7.04 | 7.27E-11 | 1.62E-09 | 14.23 |
| CETP | -1.66 | 8.33 | -14.23 | 3.02E-29 | 3.65E-26 | 55.84 |
| CFHR3 | -1.15 | 9.07 | -5.31 | 4.04E-07 | 3.98E-06 | 5.85 |
| CFI | -1.07 | 10.84 | -6.77 | 3.08E-10 | 6.00E-09 | 12.83 |
| CHST4 | -1.14 | 7.49 | -11.57 | 2.75E-22 | 8.11E-20 | 40.07 |
| CIDEB | -1.22 | 9.55 | -7.13 | 4.39E-11 | 1.02E-09 | 14.73 |
| CLDN15 | 1.20 | 8.84 | 7.50 | 5.88E-12 | 1.66E-10 | 16.69 |
| CLEC1B | -1.59 | 7.55 | -13.84 | 3.06E-28 | 3.01E-25 | 53.56 |
| CLEC4G | -1.47 | 7.62 | -16.17 | 3.61E-34 | 1.07E-30 | 66.97 |
| CLOCK | -1.15 | 8.21 | -10.34 | 4.31E-19 | 6.17E-17 | 32.83 |
| CLRN3 | -1.81 | 8.72 | -10.23 | 8.50E-19 | 1.12E-16 | 32.16 |
| CLYBL | -1.23 | 9.55 | -10.48 | 1.88E-19 | 2.98E-17 | 33.65 |
| COL1A1 | 1.49 | 8.32 | 7.39 | 1.10E-11 | 2.90E-10 | 16.09 |
| COL1A2 | 1.48 | 8.13 | 7.32 | 1.58E-11 | 4.04E-10 | 15.72 |
| COL4A1 | 1.86 | 9.58 | 11.95 | 2.63E-23 | 9.87E-21 | 42.38 |
| COLEC11 | -1.41 | 9.78 | -8.47 | 2.65E-14 | 1.22E-12 | 21.99 |
| CPEB3 | -1.21 | 8.46 | -12.35 | 2.47E-24 | 1.11E-21 | 44.71 |
| CPED1 | -1.66 | 8.63 | -15.42 | 2.71E-32 | 6.09E-29 | 62.73 |
| CPS1 | -1.79 | 12.50 | -6.63 | 6.30E-10 | 1.14E-08 | 12.13 |
| CSRNP1 | -1.37 | 9.43 | -8.64 | 1.00E-14 | 5.10E-13 | 22.95 |
| CSTB | 1.18 | 9.59 | 7.87 | 7.66E-13 | 2.58E-11 | 18.69 |
| CTH | -1.34 | 8.78 | -7.58 | 3.81E-12 | 1.11E-10 | 17.12 |
| CTHRC1 | 1.03 | 7.46 | 7.05 | 6.83E-11 | 1.53E-09 | 14.30 |
| CTNNA1 | 1.02 | 9.32 | 10.47 | 2.02E-19 | 3.16E-17 | 33.58 |
| CXCL12 | -1.37 | 8.67 | -8.80 | 3.89E-15 | 2.16E-13 | 23.88 |
| CXCL2 | -1.35 | 8.95 | -7.88 | 7.50E-13 | 2.53E-11 | 18.71 |
| CYFIP2 | -1.13 | 9.51 | -7.60 | 3.42E-12 | 1.01E-10 | 17.23 |
| CYP1A2 | -4.63 | 10.44 | -16.24 | 2.41E-34 | 7.59E-31 | 67.37 |
| CYP26A1 | -1.45 | 7.76 | -10.49 | 1.82E-19 | 2.91E-17 | 33.68 |
| CYP2A6 | -2.19 | 9.63 | -7.59 | 3.75E-12 | 1.09E-10 | 17.14 |
| CYP2A7 | -2.01 | 8.74 | -9.40 | 1.17E-16 | 9.32E-15 | 27.32 |
| CYP2C8 | -2.45 | 12.09 | -8.22 | 1.08E-13 | 4.38E-12 | 20.61 |
| CYP2C9 | -1.79 | 9.92 | -7.39 | 1.08E-11 | 2.86E-10 | 16.10 |
| CYP2E1 | -3.07 | 12.87 | -8.66 | 8.74E-15 | 4.50E-13 | 23.08 |
| CYP2J2 | -1.11 | 10.47 | -6.81 | 2.46E-10 | 4.91E-09 | 13.04 |
| CYP39A1 | -1.34 | 7.71 | -15.13 | 1.53E-31 | 2.90E-28 | 61.03 |
| CYP3A4 | -3.96 | 10.65 | -13.71 | 6.98E-28 | 6.59E-25 | 52.75 |
| CYP4A11 | -2.10 | 12.42 | -8.47 | 2.60E-14 | 1.20E-12 | 22.01 |
| CYP4F12 | -1.36 | 9.07 | -10.86 | 1.99E-20 | 3.85E-18 | 35.86 |
| CYP4F2 | -1.54 | 9.58 | -9.24 | 2.91E-16 | 2.12E-14 | 26.42 |
| CYP4V2 | -1.47 | 10.36 | -9.97 | 3.92E-18 | 4.50E-16 | 30.66 |
| CYP8B1 | -2.02 | 10.18 | -8.20 | 1.23E-13 | 4.93E-12 | 20.49 |
| DBH | -1.56 | 7.68 | -17.13 | 1.47E-36 | 5.90E-33 | 72.36 |
| DBT | -1.04 | 9.96 | -10.67 | 5.91E-20 | 1.04E-17 | 34.79 |
| DCN | -1.99 | 10.31 | -7.41 | 9.72E-12 | 2.60E-10 | 16.20 |
| DCXR | -2.05 | 11.83 | -11.13 | 3.70E-21 | 8.25E-19 | 37.51 |
| DHRS1 | -1.14 | 10.34 | -8.88 | 2.49E-15 | 1.45E-13 | 24.31 |
| DLK1 | 1.12 | 7.14 | 4.37 | 2.37E-05 | 0.000158605 | 1.94 |
| DNAJC12 | -1.06 | 8.03 | -8.54 | 1.79E-14 | 8.62E-13 | 22.38 |
| DNASE1L3 | -1.77 | 8.88 | -11.20 | 2.52E-21 | 5.86E-19 | 37.89 |
| DNMT1 | 1.22 | 8.95 | 10.23 | 8.39E-19 | 1.11E-16 | 32.18 |
| DPYS | -1.14 | 10.81 | -5.34 | 3.47E-07 | 3.48E-06 | 5.99 |
| DUSP6 | -1.13 | 10.26 | -8.10 | 2.11E-13 | 7.98E-12 | 19.96 |
| DYNLL1 | 1.00 | 10.63 | 11.71 | 1.15E-22 | 3.81E-20 | 40.93 |
| ECHS1 | -1.30 | 12.25 | -10.27 | 6.84E-19 | 9.18E-17 | 32.38 |
| ECM1 | -1.95 | 8.24 | -19.79 | 6.77E-43 | 6.40E-39 | 86.63 |
| EGR1 | -1.17 | 9.45 | -6.97 | 1.07E-10 | 2.30E-09 | 13.85 |
| ELOVL6 | -1.05 | 9.12 | -7.59 | 3.59E-12 | 1.05E-10 | 17.18 |
| ENO3 | -1.75 | 9.23 | -9.99 | 3.57E-18 | 4.14E-16 | 30.75 |
| EPHX2 | -1.11 | 10.04 | -7.20 | 3.06E-11 | 7.36E-10 | 15.08 |
| EVC2 | -1.76 | 8.43 | -15.56 | 1.25E-32 | 3.10E-29 | 63.49 |
| F11 | -1.15 | 8.29 | -11.46 | 5.12E-22 | 1.42E-19 | 39.46 |
| F12 | -1.13 | 12.06 | -5.32 | 3.97E-07 | 3.91E-06 | 5.87 |
| F7 | -1.22 | 9.58 | -7.78 | 1.25E-12 | 4.04E-11 | 18.21 |
| F9 | -2.30 | 10.79 | -11.38 | 8.31E-22 | 2.14E-19 | 38.99 |
| FADS1 | 1.04 | 8.07 | 6.28 | 3.69E-09 | 5.66E-08 | 10.40 |
| FAH | -1.01 | 9.73 | -8.53 | 1.87E-14 | 8.91E-13 | 22.34 |
| FAHD2A | -1.51 | 8.78 | -14.39 | 1.18E-29 | 1.55E-26 | 56.76 |
| FAM134B | -1.39 | 8.43 | -8.81 | 3.67E-15 | 2.05E-13 | 23.93 |
| FAM13A | -1.18 | 8.02 | -13.25 | 1.04E-26 | 7.70E-24 | 50.09 |
| FAM189B | 1.19 | 8.41 | 12.78 | 1.79E-25 | 9.81E-23 | 47.30 |
| FBP1 | -1.90 | 11.31 | -9.99 | 3.62E-18 | 4.16E-16 | 30.74 |
| FCN2 | -1.30 | 7.41 | -17.72 | 5.46E-38 | 2.87E-34 | 75.59 |
| FCN3 | -3.52 | 10.00 | -15.04 | 2.53E-31 | 4.60E-28 | 60.54 |
| FETUB | -1.36 | 9.35 | -7.45 | 7.80E-12 | 2.13E-10 | 16.42 |
| FGA | -1.57 | 9.63 | -7.94 | 5.11E-13 | 1.80E-11 | 19.09 |
| FGB | -1.47 | 13.34 | -7.88 | 7.49E-13 | 2.53E-11 | 18.71 |
| FGG | -1.54 | 11.15 | -7.58 | 3.88E-12 | 1.13E-10 | 17.10 |
| FMO3 | -1.09 | 11.22 | -4.94 | 2.18E-06 | 1.83E-05 | 4.22 |
| FOS | -1.84 | 9.08 | -8.59 | 1.28E-14 | 6.32E-13 | 22.71 |
| FOSB | -2.07 | 9.05 | -9.33 | 1.77E-16 | 1.36E-14 | 26.91 |
| FTCD | -1.62 | 8.97 | -11.35 | 1.02E-21 | 2.55E-19 | 38.78 |
| FXYD1 | -1.41 | 8.01 | -13.19 | 1.52E-26 | 1.09E-23 | 49.72 |
| G6PC | -1.10 | 10.42 | -4.62 | 8.36E-06 | 6.19E-05 | 2.94 |
| GABARAPL1 | -1.13 | 9.14 | -10.07 | 2.25E-18 | 2.69E-16 | 31.21 |
| GADD45B | -1.26 | 9.03 | -9.95 | 4.41E-18 | 4.97E-16 | 30.54 |
| GADD45G | -1.75 | 9.85 | -9.01 | 1.13E-15 | 7.13E-14 | 25.09 |
| GBA3 | -2.43 | 9.56 | -11.37 | 9.06E-22 | 2.31E-19 | 38.90 |
| GCDH | -1.33 | 8.86 | -13.06 | 3.28E-26 | 2.09E-23 | 48.97 |
| GCGR | -2.25 | 10.18 | -9.57 | 4.18E-17 | 3.69E-15 | 28.33 |
| GHR | -1.86 | 10.16 | -10.41 | 2.88E-19 | 4.28E-17 | 33.23 |
| GLMP | 1.00 | 9.95 | 9.13 | 5.70E-16 | 3.86E-14 | 25.76 |
| GLS2 | -1.50 | 8.44 | -9.57 | 4.26E-17 | 3.75E-15 | 28.31 |
| GLYAT | -1.08 | 7.42 | -12.99 | 5.03E-26 | 3.05E-23 | 48.54 |
| GLYATL1 | -1.03 | 7.76 | -12.30 | 3.24E-24 | 1.42E-21 | 44.45 |
| GMNN | 1.08 | 7.84 | 10.08 | 2.03E-18 | 2.43E-16 | 31.31 |
| GNE | -1.09 | 9.71 | -6.87 | 1.77E-10 | 3.63E-09 | 13.36 |
| GPC3 | 2.86 | 8.41 | 10.67 | 6.13E-20 | 1.08E-17 | 34.75 |
| GPER1 | -1.01 | 9.30 | -6.41 | 1.95E-09 | 3.16E-08 | 11.03 |
| GPT | -1.64 | 9.71 | -10.48 | 1.88E-19 | 2.98E-17 | 33.65 |
| GPT2 | -1.66 | 10.57 | -9.24 | 3.06E-16 | 2.21E-14 | 26.38 |
| GRHPR | -1.19 | 9.84 | -9.26 | 2.65E-16 | 1.96E-14 | 26.51 |
| GSTA1 | -1.44 | 12.09 | -5.15 | 8.24E-07 | 7.57E-06 | 5.16 |
| GSTA2 | -1.47 | 10.56 | -5.73 | 5.55E-08 | 6.55E-07 | 7.77 |
| GSTA5 | -1.77 | 10.44 | -7.50 | 6.18E-12 | 1.73E-10 | 16.65 |
| GSTM1 | -1.17 | 10.08 | -4.09 | 7.12E-05 | 0.000424927 | 0.90 |
| GSTM2 | -1.40 | 9.76 | -4.28 | 3.37E-05 | 0.000217411 | 1.61 |
| GSTZ1 | -1.70 | 8.42 | -14.71 | 1.79E-30 | 2.73E-27 | 58.61 |
| H2AFZ | 1.20 | 9.62 | 9.21 | 3.51E-16 | 2.48E-14 | 26.24 |
| H3F3A | 1.04 | 11.47 | 9.72 | 1.79E-17 | 1.74E-15 | 29.17 |
| H3F3AP4 | 1.01 | 11.43 | 8.92 | 2.00E-15 | 1.19E-13 | 24.53 |
| HAMP | -3.94 | 10.67 | -11.77 | 7.97E-23 | 2.83E-20 | 41.29 |
| HAO1 | -1.55 | 10.88 | -9.62 | 3.25E-17 | 2.96E-15 | 28.58 |
| HAO2 | -1.11 | 7.76 | -11.16 | 3.11E-21 | 7.10E-19 | 37.69 |
| HBA1 | -1.01 | 8.52 | -5.65 | 8.24E-08 | 9.37E-07 | 7.39 |
| HBA2 | -2.04 | 10.18 | -11.60 | 2.26E-22 | 6.80E-20 | 40.27 |
| HBB | -1.21 | 9.65 | -5.33 | 3.68E-07 | 3.66E-06 | 5.94 |
| HEPACAM | -1.62 | 8.26 | -9.12 | 6.12E-16 | 4.11E-14 | 25.69 |
| HGD | -1.13 | 8.40 | -11.75 | 9.02E-23 | 3.13E-20 | 41.17 |
| HGFAC | -2.33 | 9.71 | -9.91 | 5.75E-18 | 6.27E-16 | 30.28 |
| HMGCL | -1.40 | 9.46 | -13.74 | 5.56E-28 | 5.36E-25 | 52.98 |
| HMGN1 | 1.02 | 10.38 | 7.75 | 1.55E-12 | 4.90E-11 | 18.00 |
| HOGA1 | -1.42 | 8.46 | -13.48 | 2.66E-27 | 2.16E-24 | 51.44 |
| HP | -1.48 | 13.78 | -6.19 | 6.01E-09 | 8.76E-08 | 9.93 |
| HPD | -2.06 | 10.94 | -7.19 | 3.22E-11 | 7.71E-10 | 15.03 |
| HPR | -1.08 | 9.00 | -6.43 | 1.76E-09 | 2.89E-08 | 11.12 |
| HPX | -1.61 | 12.60 | -6.87 | 1.82E-10 | 3.71E-09 | 13.34 |
| HRG | -2.27 | 10.61 | -9.41 | 1.09E-16 | 8.72E-15 | 27.39 |
| HSD11B1 | -1.55 | 9.94 | -5.61 | 9.84E-08 | 1.10E-06 | 7.21 |
| HSD17B13 | -2.88 | 9.66 | -11.87 | 4.32E-23 | 1.58E-20 | 41.90 |
| HSD17B2 | -1.02 | 9.83 | -6.07 | 1.07E-08 | 1.48E-07 | 9.37 |
| HSP90AA1 | 1.07 | 9.87 | 8.45 | 2.97E-14 | 1.35E-12 | 21.88 |
| HSP90AB1 | 1.22 | 10.20 | 11.36 | 9.71E-22 | 2.45E-19 | 38.83 |
| HYAL1 | -1.12 | 10.98 | -7.73 | 1.72E-12 | 5.41E-11 | 17.90 |
| IGF1 | -1.69 | 8.27 | -11.90 | 3.72E-23 | 1.37E-20 | 42.04 |
| IGFALS | -2.22 | 8.56 | -17.13 | 1.50E-36 | 5.90E-33 | 72.34 |
| IGFBP3 | -1.89 | 9.92 | -13.30 | 7.82E-27 | 5.86E-24 | 50.37 |
| IGLL1 | -1.61 | 10.77 | -4.55 | 1.13E-05 | 8.10E-05 | 2.65 |
| ILF2 | 1.28 | 8.90 | 12.58 | 6.12E-25 | 3.08E-22 | 46.09 |
| INMT | -1.42 | 8.09 | -10.45 | 2.28E-19 | 3.50E-17 | 33.46 |
| ITIH1 | -1.19 | 11.88 | -6.00 | 1.51E-08 | 2.02E-07 | 9.04 |
| KBTBD11 | -1.46 | 7.98 | -11.93 | 3.09E-23 | 1.15E-20 | 42.23 |
| KLKB1 | -1.83 | 9.79 | -12.42 | 1.55E-24 | 7.19E-22 | 45.17 |
| KMO | -1.46 | 8.33 | -13.09 | 2.75E-26 | 1.78E-23 | 49.14 |
| LAMC1 | 1.31 | 9.14 | 9.88 | 6.66E-18 | 7.18E-16 | 30.14 |
| LAPTM4B | 1.54 | 8.64 | 9.55 | 4.81E-17 | 4.14E-15 | 28.19 |
| LCAT | -2.57 | 8.85 | -19.39 | 5.66E-42 | 4.05E-38 | 84.55 |
| LCN2 | 1.17 | 8.18 | 4.75 | 4.91E-06 | 3.83E-05 | 3.44 |
| LEAP2 | -1.16 | 11.41 | -6.09 | 9.64E-09 | 1.35E-07 | 9.47 |
| LECT2 | -1.98 | 10.13 | -9.97 | 4.12E-18 | 4.69E-16 | 30.61 |
| LEPR | -1.29 | 10.60 | -6.45 | 1.59E-09 | 2.64E-08 | 11.23 |
| LIME1 | -1.15 | 11.02 | -8.46 | 2.76E-14 | 1.26E-12 | 21.95 |
| LIPC | -1.29 | 10.39 | -7.16 | 3.87E-11 | 9.12E-10 | 14.85 |
| LONP2 | -1.09 | 8.53 | -9.02 | 1.08E-15 | 6.88E-14 | 25.14 |
| LY6E | -2.06 | 10.50 | -10.27 | 6.65E-19 | 8.97E-17 | 32.41 |
| LYZ | 1.40 | 8.61 | 4.74 | 4.99E-06 | 3.88E-05 | 3.43 |
| MASP1 | -1.02 | 7.88 | -12.87 | 1.05E-25 | 5.85E-23 | 47.82 |
| MAT1A | -1.64 | 10.12 | -10.95 | 1.10E-20 | 2.21E-18 | 36.44 |
| MBL2 | -1.24 | 9.32 | -6.36 | 2.50E-09 | 3.97E-08 | 10.78 |
| MCM3 | 1.16 | 9.25 | 10.80 | 2.70E-20 | 5.13E-18 | 35.56 |
| MCM6 | 1.11 | 8.48 | 10.38 | 3.56E-19 | 5.15E-17 | 33.02 |
| MDK | 1.81 | 8.36 | 10.48 | 1.91E-19 | 3.01E-17 | 33.63 |
| METTL7A | -1.05 | 11.82 | -8.57 | 1.45E-14 | 7.10E-13 | 22.59 |
| METTL7B | -1.08 | 9.68 | -8.04 | 2.92E-13 | 1.08E-11 | 19.64 |
| MFAP4 | -1.07 | 8.12 | -7.21 | 2.85E-11 | 6.88E-10 | 15.15 |
| MGMT | -1.11 | 10.50 | -11.32 | 1.20E-21 | 2.96E-19 | 38.63 |
| MIR99AHG | -1.02 | 8.08 | -11.41 | 7.22E-22 | 1.89E-19 | 39.12 |
| MPC2 | 1.21 | 10.30 | 8.95 | 1.62E-15 | 9.90E-14 | 24.74 |
| MST1 | -1.11 | 11.90 | -7.74 | 1.64E-12 | 5.18E-11 | 17.94 |
| MT1A | -3.00 | 12.45 | -12.43 | 1.46E-24 | 6.83E-22 | 45.23 |
| MT1E | -3.28 | 11.77 | -13.04 | 3.83E-26 | 2.38E-23 | 48.81 |
| MT1F | -3.68 | 10.40 | -16.31 | 1.59E-34 | 5.36E-31 | 67.77 |
| MT1G | -4.05 | 11.92 | -14.09 | 7.02E-29 | 8.09E-26 | 55.01 |
| MT1H | -4.01 | 10.25 | -14.63 | 2.85E-30 | 3.97E-27 | 58.16 |
| MT1M | -2.29 | 8.32 | -12.11 | 1.04E-23 | 4.11E-21 | 43.30 |
| MT1X | -3.17 | 11.23 | -12.01 | 1.88E-23 | 7.15E-21 | 42.72 |
| MT2A | -2.66 | 11.85 | -11.12 | 4.13E-21 | 9.11E-19 | 37.41 |
| MTTP | -1.32 | 10.65 | -7.50 | 6.07E-12 | 1.70E-10 | 16.66 |
| NAT2 | -2.68 | 8.66 | -20.37 | 3.20E-44 | 5.03E-40 | 89.60 |
| NAT8B | -1.00 | 9.61 | -6.41 | 1.91E-09 | 3.11E-08 | 11.04 |
| NDRG2 | -1.44 | 8.80 | -12.15 | 8.03E-24 | 3.27E-21 | 43.55 |
| NEK2 | 1.27 | 7.46 | 11.55 | 3.02E-22 | 8.87E-20 | 39.98 |
| NNMT | -2.27 | 9.85 | -9.49 | 6.94E-17 | 5.76E-15 | 27.83 |
| NNT | -1.07 | 8.44 | -9.25 | 2.85E-16 | 2.09E-14 | 26.45 |
| NPC1L1 | -1.15 | 8.10 | -7.87 | 7.67E-13 | 2.58E-11 | 18.69 |
| NPW | -2.01 | 9.62 | -8.69 | 7.48E-15 | 3.90E-13 | 23.23 |
| NQO1 | 1.15 | 8.25 | 5.36 | 3.30E-07 | 3.32E-06 | 6.04 |
| NTHL1 | -1.11 | 9.03 | -11.76 | 8.71E-23 | 3.05E-20 | 41.21 |
| NUSAP1 | 1.27 | 7.68 | 13.30 | 7.80E-27 | 5.86E-24 | 50.38 |
| OGDHL | -1.39 | 8.73 | -11.42 | 6.44E-22 | 1.71E-19 | 39.24 |
| ORM2 | -1.05 | 12.90 | -5.38 | 2.98E-07 | 3.02E-06 | 6.14 |
| OTC | -1.14 | 10.14 | -6.31 | 3.24E-09 | 5.01E-08 | 10.53 |
| PABPC1 | 1.25 | 11.25 | 9.20 | 3.68E-16 | 2.59E-14 | 26.19 |
| PAH | -1.41 | 11.43 | -8.89 | 2.34E-15 | 1.37E-13 | 24.38 |
| PAMR1 | -1.08 | 7.56 | -12.89 | 9.15E-26 | 5.25E-23 | 47.96 |
| PANK1 | -1.39 | 9.79 | -11.64 | 1.74E-22 | 5.38E-20 | 40.52 |
| PCK1 | -2.80 | 10.11 | -12.23 | 5.12E-24 | 2.20E-21 | 43.99 |
| PDK4 | -1.21 | 9.54 | -6.63 | 6.44E-10 | 1.17E-08 | 12.10 |
| PEG10 | 1.55 | 7.71 | 7.11 | 4.97E-11 | 1.15E-09 | 14.61 |
| PEMT | -1.43 | 10.13 | -12.10 | 1.10E-23 | 4.34E-21 | 43.24 |
| PEX11G | -1.38 | 8.45 | -15.00 | 3.32E-31 | 5.81E-28 | 60.27 |
| PGLYRP2 | -2.24 | 11.14 | -10.70 | 4.97E-20 | 8.89E-18 | 34.96 |
| PHGDH | -1.55 | 10.54 | -8.12 | 1.93E-13 | 7.36E-12 | 20.04 |
| PHYH | -1.06 | 12.38 | -7.73 | 1.67E-12 | 5.25E-11 | 17.93 |
| PLA2G16 | -1.48 | 8.31 | -14.33 | 1.75E-29 | 2.18E-26 | 56.38 |
| PLG | -1.32 | 11.70 | -5.66 | 7.80E-08 | 8.90E-07 | 7.44 |
| PLGLB1 | -1.09 | 10.32 | -5.54 | 1.40E-07 | 1.52E-06 | 6.87 |
| PMPCA | -1.05 | 9.29 | -9.50 | 6.63E-17 | 5.54E-15 | 27.88 |
| PNPLA7 | -1.59 | 8.52 | -13.92 | 1.98E-28 | 2.13E-25 | 53.99 |
| PON1 | -1.75 | 9.24 | -11.70 | 1.23E-22 | 3.99E-20 | 40.86 |
| PON3 | -1.34 | 11.62 | -8.72 | 6.20E-15 | 3.30E-13 | 23.42 |
| PPARGC1A | -1.32 | 9.22 | -7.73 | 1.66E-12 | 5.24E-11 | 17.93 |
| PPIAL4A | 1.32 | 11.91 | 9.48 | 7.19E-17 | 5.95E-15 | 27.80 |
| PRC1 | 1.13 | 7.34 | 13.41 | 4.01E-27 | 3.21E-24 | 51.03 |
| PROC | -1.16 | 10.43 | -8.27 | 8.16E-14 | 3.40E-12 | 20.89 |
| PRODH2 | -1.46 | 9.61 | -9.55 | 4.94E-17 | 4.22E-15 | 28.17 |
| PROZ | -1.30 | 8.15 | -9.17 | 4.57E-16 | 3.17E-14 | 25.98 |
| PRSS8 | -1.16 | 7.91 | -10.49 | 1.82E-19 | 2.91E-17 | 33.68 |
| PSAT1 | -1.24 | 9.31 | -8.60 | 1.21E-14 | 6.04E-13 | 22.76 |
| PSMD4 | 1.11 | 9.46 | 11.52 | 3.61E-22 | 1.04E-19 | 39.81 |
| PTH1R | -2.03 | 8.14 | -19.88 | 4.11E-43 | 4.85E-39 | 87.11 |
| PTTG1 | 1.24 | 7.87 | 11.57 | 2.70E-22 | 8.01E-20 | 40.09 |
| PZP | -1.14 | 7.48 | -6.83 | 2.22E-10 | 4.47E-09 | 13.14 |
| QDPR | -1.00 | 10.45 | -7.77 | 1.39E-12 | 4.44E-11 | 18.11 |
| RAB17 | -1.09 | 10.30 | -8.81 | 3.68E-15 | 2.05E-13 | 23.93 |
| RAB33B | -1.12 | 8.69 | -11.69 | 1.30E-22 | 4.11E-20 | 40.81 |
| RAD51 | -1.66 | 9.50 | -11.01 | 7.69E-21 | 1.59E-18 | 36.79 |
| RBP4 | -1.19 | 12.33 | -6.09 | 9.69E-09 | 1.35E-07 | 9.46 |
| RBP5 | -1.01 | 8.91 | -7.56 | 4.33E-12 | 1.25E-10 | 16.99 |
| RCAN1 | -1.14 | 8.86 | -9.64 | 2.90E-17 | 2.68E-15 | 28.69 |
| RCL1 | -1.13 | 8.32 | -9.69 | 2.08E-17 | 2.01E-15 | 29.02 |
| RDH16 | -2.58 | 10.62 | -12.29 | 3.38E-24 | 1.47E-21 | 44.40 |
| RDH5 | -2.16 | 8.98 | -17.22 | 9.03E-37 | 4.27E-33 | 72.84 |
| REEP6 | -1.16 | 10.38 | -6.55 | 9.38E-10 | 1.64E-08 | 11.74 |
| RFX5 | 1.09 | 7.95 | 13.22 | 1.30E-26 | 9.46E-24 | 49.87 |
| RHEB | 1.06 | 9.37 | 9.59 | 3.71E-17 | 3.35E-15 | 28.45 |
| RIDA | -1.71 | 10.47 | -10.65 | 6.67E-20 | 1.16E-17 | 34.67 |
| RNASE4 | -1.18 | 10.33 | -4.05 | 8.24E-05 | 0.000484072 | 0.76 |
| RPL13AP5 | 1.11 | 12.11 | 7.78 | 1.32E-12 | 4.25E-11 | 18.16 |
| RPL15 | 1.29 | 8.11 | 8.88 | 2.42E-15 | 1.41E-13 | 24.34 |
| RPL17 | 1.04 | 11.47 | 8.33 | 5.72E-14 | 2.46E-12 | 21.24 |
| RPL30 | 1.13 | 11.57 | 6.39 | 2.19E-09 | 3.51E-08 | 10.91 |
| RPLP0 | 1.55 | 10.77 | 5.48 | 1.87E-07 | 1.97E-06 | 6.60 |
| RPS12 | 1.07 | 12.37 | 6.71 | 4.07E-10 | 7.71E-09 | 12.55 |
| RPS27 | 1.04 | 13.28 | 7.32 | 1.60E-11 | 4.07E-10 | 15.72 |
| RPS5 | 1.08 | 11.06 | 6.95 | 1.18E-10 | 2.50E-09 | 13.77 |
| S100A10 | 1.10 | 10.84 | 6.90 | 1.52E-10 | 3.15E-09 | 13.52 |
| S100P | 1.05 | 7.18 | 4.75 | 4.87E-06 | 3.80E-05 | 3.45 |
| SAA1 | -1.54 | 8.64 | -6.03 | 1.34E-08 | 1.82E-07 | 9.15 |
| SAA2 | -1.65 | 9.05 | -5.45 | 2.18E-07 | 2.27E-06 | 6.45 |
| SAA4 | -2.21 | 11.99 | -9.35 | 1.58E-16 | 1.23E-14 | 27.02 |
| SC5D | -1.15 | 9.83 | -8.07 | 2.54E-13 | 9.49E-12 | 19.77 |
| SDS | -2.30 | 10.01 | -9.20 | 3.69E-16 | 2.60E-14 | 26.19 |
| SEPP1 | -1.64 | 9.20 | -13.85 | 2.95E-28 | 2.97E-25 | 53.60 |
| SERPINA11 | -2.01 | 10.14 | -11.15 | 3.30E-21 | 7.42E-19 | 37.63 |
| SERPINA4 | -1.07 | 8.06 | -10.98 | 9.59E-21 | 1.95E-18 | 36.58 |
| SERPINA5 | -1.08 | 11.48 | -6.45 | 1.56E-09 | 2.59E-08 | 11.24 |
| SERPINA7 | -1.03 | 10.43 | -5.04 | 1.40E-06 | 1.22E-05 | 4.65 |
| SERPINF2 | -1.42 | 11.34 | -7.99 | 4.06E-13 | 1.45E-11 | 19.31 |
| SERPING1 | -1.08 | 11.97 | -5.90 | 2.50E-08 | 3.20E-07 | 8.54 |
| SF3B4 | 1.11 | 9.34 | 11.30 | 1.34E-21 | 3.29E-19 | 38.51 |
| SHBG | -2.08 | 9.07 | -13.12 | 2.28E-26 | 1.52E-23 | 49.32 |
| SHMT1 | -1.26 | 10.40 | -7.08 | 5.78E-11 | 1.31E-09 | 14.46 |
| SKAP1 | -1.92 | 8.83 | -14.96 | 4.03E-31 | 6.57E-28 | 60.08 |
| SLC10A1 | -1.81 | 10.57 | -7.33 | 1.55E-11 | 3.96E-10 | 15.75 |
| SLC13A5 | -1.55 | 11.31 | -6.58 | 8.25E-10 | 1.46E-08 | 11.86 |
| SLC17A2 | -1.15 | 9.53 | -7.42 | 9.31E-12 | 2.51E-10 | 16.24 |
| SLC19A3 | -1.21 | 8.08 | -14.65 | 2.54E-30 | 3.75E-27 | 58.27 |
| SLC22A1 | -2.47 | 9.49 | -12.74 | 2.25E-25 | 1.21E-22 | 47.07 |
| SLC22A10 | -1.77 | 8.19 | -14.52 | 5.36E-30 | 7.23E-27 | 57.54 |
| SLC25A18 | -1.43 | 9.33 | -8.81 | 3.60E-15 | 2.02E-13 | 23.95 |
| SLC25A47 | -1.99 | 8.68 | -13.06 | 3.43E-26 | 2.16E-23 | 48.92 |
| SLC27A5 | -2.05 | 10.26 | -10.66 | 6.39E-20 | 1.12E-17 | 34.71 |
| SLC2A2 | -1.05 | 11.36 | -5.19 | 7.00E-07 | 6.54E-06 | 5.32 |
| SLC38A2 | -1.09 | 10.71 | -7.75 | 1.52E-12 | 4.81E-11 | 18.02 |
| SLC38A4 | -1.60 | 11.20 | -9.59 | 3.74E-17 | 3.36E-15 | 28.44 |
| SLC39A5 | -1.71 | 9.67 | -9.28 | 2.32E-16 | 1.73E-14 | 26.65 |
| SLC6A12 | -1.14 | 8.81 | -8.45 | 2.91E-14 | 1.33E-12 | 21.90 |
| SLC7A2 | -1.36 | 10.32 | -7.64 | 2.86E-12 | 8.56E-11 | 17.40 |
| SLCO1B3 | -2.13 | 8.57 | -11.17 | 3.06E-21 | 7.06E-19 | 37.70 |
| SMIM24 | -1.65 | 8.67 | -12.37 | 2.18E-24 | 9.90E-22 | 44.83 |
| SNHG6 | 1.01 | 8.88 | 8.89 | 2.25E-15 | 1.32E-13 | 24.41 |
| SNORA22 | -1.14 | 7.67 | -9.68 | 2.28E-17 | 2.18E-15 | 28.93 |
| SNORD13 | -1.57 | 9.41 | -9.81 | 1.03E-17 | 1.05E-15 | 29.71 |
| SOCS2 | -1.52 | 9.17 | -9.31 | 2.02E-16 | 1.53E-14 | 26.79 |
| SORD | -1.28 | 10.74 | -7.49 | 6.53E-12 | 1.81E-10 | 16.59 |
| SPINK1 | 2.88 | 8.82 | 7.89 | 7.03E-13 | 2.39E-11 | 18.78 |
| SPP1 | 1.44 | 8.47 | 5.39 | 2.79E-07 | 2.84E-06 | 6.21 |
| SPP2 | -1.48 | 10.30 | -6.10 | 9.24E-09 | 1.30E-07 | 9.51 |
| SQLE | 1.42 | 8.30 | 9.32 | 1.88E-16 | 1.44E-14 | 26.85 |
| SRD5A2 | -1.27 | 7.67 | -12.62 | 4.76E-25 | 2.44E-22 | 46.33 |
| SRP14 | 1.09 | 10.19 | 9.08 | 7.80E-16 | 5.08E-14 | 25.45 |
| SSR2 | 1.19 | 8.45 | 8.85 | 2.89E-15 | 1.65E-13 | 24.17 |
| ST3GAL6 | -1.06 | 8.22 | -11.40 | 7.63E-22 | 1.99E-19 | 39.07 |
| STEAP3 | -1.34 | 9.09 | -10.74 | 4.00E-20 | 7.38E-18 | 35.17 |
| STOM | -1.09 | 9.29 | -6.74 | 3.50E-10 | 6.72E-09 | 12.70 |
| SULT1A1 | -1.18 | 9.06 | -11.15 | 3.46E-21 | 7.75E-19 | 37.58 |
| SULT2A1 | -1.43 | 11.82 | -6.86 | 1.92E-10 | 3.91E-09 | 13.29 |
| SYT7 | -1.04 | 9.65 | -5.14 | 8.87E-07 | 8.08E-06 | 5.09 |
| TAGLN2 | 1.00 | 9.13 | 8.87 | 2.60E-15 | 1.50E-13 | 24.27 |
| TAT | -2.69 | 10.85 | -9.94 | 4.77E-18 | 5.33E-16 | 30.47 |
| TDO2 | -1.46 | 9.28 | -7.43 | 8.70E-12 | 2.36E-10 | 16.31 |
| THRSP | -2.49 | 9.47 | -10.43 | 2.56E-19 | 3.87E-17 | 33.35 |
| THY1 | 1.28 | 7.71 | 9.85 | 7.99E-18 | 8.46E-16 | 29.96 |
| TKFC | -1.11 | 8.39 | -8.40 | 3.94E-14 | 1.75E-12 | 21.60 |
| TM6SF2 | -1.09 | 8.07 | -9.68 | 2.26E-17 | 2.17E-15 | 28.94 |
| TMEM47 | -1.12 | 8.75 | -10.73 | 4.23E-20 | 7.69E-18 | 35.12 |
| TOMM20 | 1.03 | 8.41 | 7.71 | 1.89E-12 | 5.91E-11 | 17.80 |
| TOP2A | 1.48 | 7.41 | 13.10 | 2.59E-26 | 1.70E-23 | 49.20 |
| TPR | -1.76 | 11.25 | -8.23 | 1.00E-13 | 4.10E-12 | 20.69 |
| TRPM8 | -1.53 | 8.86 | -11.26 | 1.72E-21 | 4.15E-19 | 38.27 |
| TSKU | -1.17 | 10.19 | -6.34 | 2.80E-09 | 4.38E-08 | 10.67 |
| TTC36 | -2.22 | 8.05 | -21.39 | 1.53E-46 | 3.62E-42 | 94.80 |
| TTR | -1.21 | 13.91 | -5.24 | 5.64E-07 | 5.37E-06 | 5.53 |
| TUBA1A | 1.36 | 10.99 | 10.66 | 6.62E-20 | 1.15E-17 | 34.68 |
| TUBA1B | 1.23 | 11.31 | 9.85 | 8.14E-18 | 8.56E-16 | 29.94 |
| TUBA1C | 1.73 | 9.22 | 10.20 | 1.02E-18 | 1.31E-16 | 31.98 |
| TXNRD1 | 1.02 | 7.97 | 8.51 | 2.08E-14 | 9.77E-13 | 22.23 |
| UBD | 2.01 | 9.38 | 9.29 | 2.28E-16 | 1.71E-14 | 26.67 |
| UBE2C | 1.46 | 7.45 | 12.56 | 6.61E-25 | 3.29E-22 | 46.01 |
| UGP2 | -2.26 | 10.13 | -14.72 | 1.73E-30 | 2.72E-27 | 58.65 |
| UGT2B10 | -2.01 | 11.56 | -8.77 | 4.52E-15 | 2.49E-13 | 23.73 |
| UGT2B11 | -1.62 | 11.50 | -8.44 | 3.01E-14 | 1.37E-12 | 21.87 |
| UGT2B17 | -1.15 | 12.43 | -5.19 | 7.07E-07 | 6.60E-06 | 5.31 |
| UGT2B28 | -1.21 | 9.09 | -7.15 | 4.02E-11 | 9.44E-10 | 14.81 |
| UPB1 | -1.28 | 10.73 | -6.56 | 9.26E-10 | 1.62E-08 | 11.75 |
| UQCRH | 1.06 | 10.08 | 7.22 | 2.82E-11 | 6.82E-10 | 15.16 |
| VIPR1 | -1.78 | 8.09 | -19.38 | 6.00E-42 | 4.05E-38 | 84.50 |
| WDR72 | -1.70 | 9.05 | -10.43 | 2.56E-19 | 3.87E-17 | 33.35 |
| YWHAZ | 1.16 | 9.89 | 9.56 | 4.67E-17 | 4.06E-15 | 28.22 |
| ZG16 | -1.74 | 8.36 | -13.91 | 2.07E-28 | 2.17E-25 | 53.95 |
| ZGPAT | -1.89 | 10.10 | -14.98 | 3.66E-31 | 6.17E-28 | 60.18 |
| ZNF860 | -1.15 | 8.46 | -8.28 | 7.81E-14 | 3.27E-12 | 20.93 |

Table 3: List of DEGs for GSE36376 datasets

| **Symbol** | **logFC** | **AveExpr** | **t** | **P.Value** | **adj.P.Val** | **B** |
| --- | --- | --- | --- | --- | --- | --- |
| A2M | -1.11 | 12.51 | -5.37 | 7.84E-07 | 1.44E-05 | 5.51 |
| AADAT | -1.86 | 10.06 | -12.53 | 1.98E-20 | 1.17E-16 | 35.84 |
| ABCA8 | -1.39 | 10.03 | -7.12 | 4.43E-10 | 3.06E-08 | 12.74 |
| ACAA2 | -1.08 | 12.00 | -6.43 | 9.13E-09 | 3.54E-07 | 9.81 |
| ACACB | -1.33 | 11.22 | -7.30 | 2.04E-10 | 1.63E-08 | 13.50 |
| ACADS | -1.30 | 9.86 | -8.39 | 1.56E-12 | 3.57E-10 | 18.23 |
| ACSL1 | -1.07 | 11.70 | -6.17 | 2.76E-08 | 8.84E-07 | 8.74 |
| ACSL4 | 1.63 | 9.18 | 5.49 | 4.85E-07 | 9.76E-06 | 5.97 |
| ACSM3 | -1.63 | 10.52 | -8.65 | 4.91E-13 | 1.47E-10 | 19.35 |
| ACSM5 | -1.23 | 10.58 | -5.82 | 1.21E-07 | 3.05E-06 | 7.31 |
| ACTG2 | 1.24 | 8.15 | 5.73 | 1.74E-07 | 4.18E-06 | 6.96 |
| ADAMTSL2 | -1.68 | 9.50 | -8.78 | 2.69E-13 | 8.97E-11 | 19.94 |
| ADGRG7 | -1.28 | 8.79 | -10.21 | 4.58E-16 | 4.52E-13 | 26.13 |
| ADH1B | -2.18 | 11.51 | -8.19 | 3.79E-12 | 7.11E-10 | 17.37 |
| ADH1C | -1.45 | 13.16 | -5.00 | 3.40E-06 | 4.96E-05 | 4.10 |
| ADH4 | -2.25 | 12.90 | -5.78 | 1.46E-07 | 3.55E-06 | 7.13 |
| ADH6 | -1.16 | 10.34 | -6.23 | 2.15E-08 | 7.19E-07 | 8.98 |
| ADIRF | -1.29 | 10.61 | -4.92 | 4.75E-06 | 6.62E-05 | 3.78 |
| AFM | -1.88 | 13.19 | -7.17 | 3.68E-10 | 2.62E-08 | 12.92 |
| AGL | -1.02 | 10.58 | -6.50 | 6.70E-09 | 2.76E-07 | 10.11 |
| AGXT | -1.13 | 13.85 | -5.24 | 1.30E-06 | 2.19E-05 | 5.03 |
| AGXT2 | -1.05 | 9.89 | -4.80 | 7.34E-06 | 9.51E-05 | 3.37 |
| AKR1B10 | 2.81 | 10.34 | 6.02 | 5.25E-08 | 1.52E-06 | 8.12 |
| AKR1B15 | 2.18 | 8.47 | 6.42 | 9.37E-09 | 3.61E-07 | 9.79 |
| AKR1C3 | 1.38 | 13.09 | 10.61 | 7.67E-17 | 1.04E-13 | 27.86 |
| AKR1D1 | -1.57 | 11.33 | -5.02 | 3.10E-06 | 4.59E-05 | 4.19 |
| AKR7A3 | -1.19 | 12.56 | -5.00 | 3.42E-06 | 4.99E-05 | 4.10 |
| ALDH2 | -1.17 | 12.98 | -6.59 | 4.58E-09 | 2.00E-07 | 10.48 |
| ALDH6A1 | -1.26 | 12.44 | -6.99 | 7.96E-10 | 4.83E-08 | 12.17 |
| ALDH8A1 | -1.23 | 10.82 | -6.33 | 1.39E-08 | 5.00E-07 | 9.40 |
| ALG1L | 1.45 | 7.74 | 8.29 | 2.41E-12 | 5.05E-10 | 17.81 |
| ALPL | -1.33 | 10.59 | -5.76 | 1.58E-07 | 3.81E-06 | 7.06 |
| ANGPTL4 | -1.28 | 11.76 | -5.09 | 2.37E-06 | 3.63E-05 | 4.45 |
| ANGPTL6 | -1.18 | 8.76 | -11.20 | 5.99E-18 | 1.42E-14 | 30.33 |
| ANKRD37 | -1.00 | 8.99 | -4.93 | 4.41E-06 | 6.21E-05 | 3.85 |
| ANO1 | -1.01 | 10.01 | -4.44 | 2.91E-05 | 0.000306 | 2.05 |
| ANXA10 | -1.81 | 10.50 | -7.75 | 2.72E-11 | 3.43E-09 | 15.45 |
| ANXA2 | 1.12 | 8.62 | 7.79 | 2.25E-11 | 2.96E-09 | 15.64 |
| APOA5 | -1.76 | 11.18 | -6.88 | 1.27E-09 | 6.96E-08 | 11.72 |
| APOC4 | -1.26 | 12.60 | -5.06 | 2.64E-06 | 4.00E-05 | 4.35 |
| APOF | -2.39 | 11.50 | -7.73 | 3.04E-11 | 3.71E-09 | 15.35 |
| APOPT1 | -1.01 | 11.10 | -6.92 | 1.07E-09 | 6.08E-08 | 11.89 |
| ARHGAP10 | -1.16 | 8.47 | -10.02 | 1.05E-15 | 9.59E-13 | 25.32 |
| ASGR2 | -1.00 | 12.35 | -6.89 | 1.23E-09 | 6.79E-08 | 11.75 |
| ASPDH | -1.26 | 9.28 | -8.34 | 1.91E-12 | 4.27E-10 | 18.03 |
| ASPG | -2.18 | 9.46 | -10.40 | 2.01E-16 | 2.31E-13 | 26.93 |
| ASPM | 1.35 | 8.72 | 8.09 | 5.92E-12 | 1.03E-09 | 16.94 |
| ASS1 | -1.38 | 13.51 | -8.54 | 8.00E-13 | 2.15E-10 | 18.88 |
| ATF3 | -1.17 | 10.33 | -5.30 | 1.02E-06 | 1.79E-05 | 5.26 |
| ATF5 | -1.54 | 12.48 | -6.07 | 4.19E-08 | 1.26E-06 | 8.34 |
| ATOH8 | -1.29 | 8.96 | -9.25 | 3.34E-14 | 1.76E-11 | 21.96 |
| AURKA | 1.42 | 8.30 | 8.60 | 6.20E-13 | 1.73E-10 | 19.13 |
| AVPI1 | -1.14 | 11.49 | -9.03 | 8.82E-14 | 3.97E-11 | 21.02 |
| AZGP1 | -1.32 | 11.79 | -7.81 | 2.05E-11 | 2.78E-09 | 15.73 |
| BAIAP2L2 | 1.35 | 8.20 | 6.49 | 6.96E-09 | 2.85E-07 | 10.07 |
| BBOX1 | -1.26 | 10.57 | -3.55 | 0.000655 | 0.004344 | -0.88 |
| BCHE | -1.82 | 11.08 | -6.64 | 3.60E-09 | 1.64E-07 | 10.71 |
| BGN | -1.16 | 12.64 | -5.13 | 2.06E-06 | 3.23E-05 | 4.59 |
| BHMT | -1.38 | 13.12 | -4.88 | 5.49E-06 | 7.48E-05 | 3.64 |
| BOLA2 | 1.24 | 10.08 | 8.90 | 1.60E-13 | 6.25E-11 | 20.44 |
| BOP1 | 1.09 | 9.73 | 7.31 | 1.98E-10 | 1.60E-08 | 13.53 |
| BRSK1 | 1.08 | 8.56 | 6.47 | 7.71E-09 | 3.09E-07 | 9.97 |
| C17orf58 | 1.01 | 9.06 | 7.71 | 3.25E-11 | 3.91E-09 | 15.28 |
| C1QTNF1 | -1.28 | 9.65 | -6.85 | 1.46E-09 | 7.77E-08 | 11.58 |
| C1R | -1.16 | 11.79 | -5.59 | 3.15E-07 | 6.85E-06 | 6.39 |
| C1RL | -1.03 | 10.53 | -7.80 | 2.21E-11 | 2.93E-09 | 15.65 |
| C6 | -1.40 | 13.05 | -5.77 | 1.47E-07 | 3.58E-06 | 7.12 |
| C7 | -1.80 | 8.95 | -7.06 | 5.76E-10 | 3.79E-08 | 12.49 |
| C8A | -1.42 | 13.22 | -6.57 | 5.01E-09 | 2.15E-07 | 10.39 |
| C8B | -1.09 | 12.78 | -6.18 | 2.71E-08 | 8.71E-07 | 8.76 |
| C8orf4 | -1.62 | 10.25 | -7.59 | 5.56E-11 | 5.97E-09 | 14.76 |
| C9 | -2.06 | 10.33 | -6.36 | 1.24E-08 | 4.55E-07 | 9.51 |
| CABYR | 1.06 | 7.30 | 5.27 | 1.16E-06 | 1.98E-05 | 5.14 |
| CAMK2B | -1.34 | 8.67 | -12.61 | 1.42E-20 | 1.12E-16 | 36.16 |
| CAP2 | 1.90 | 9.60 | 10.94 | 1.87E-17 | 3.69E-14 | 29.23 |
| CBS | -1.06 | 12.03 | -4.73 | 9.67E-06 | 0.000121 | 3.10 |
| CCL14 | -1.24 | 10.41 | -7.24 | 2.63E-10 | 2.00E-08 | 13.25 |
| CCL20 | 2.25 | 9.17 | 6.07 | 4.26E-08 | 1.28E-06 | 8.32 |
| CCNB2 | 1.38 | 7.93 | 8.63 | 5.42E-13 | 1.57E-10 | 19.26 |
| CCT3 | 1.07 | 10.68 | 8.53 | 8.42E-13 | 2.23E-10 | 18.83 |
| CD14 | -1.18 | 12.10 | -5.77 | 1.47E-07 | 3.58E-06 | 7.12 |
| CD163L1 | 1.05 | 7.88 | 6.04 | 4.83E-08 | 1.42E-06 | 8.20 |
| CD34 | 1.80 | 8.52 | 13.37 | 6.00E-22 | 9.46E-18 | 39.21 |
| CDC20 | 2.05 | 8.28 | 8.89 | 1.68E-13 | 6.43E-11 | 20.40 |
| CDC45 | 1.16 | 7.60 | 8.84 | 2.11E-13 | 7.61E-11 | 20.17 |
| CDCA5 | 1.16 | 8.36 | 9.18 | 4.46E-14 | 2.25E-11 | 21.68 |
| CDHR2 | -1.61 | 9.10 | -9.53 | 9.46E-15 | 5.81E-12 | 23.19 |
| CDKN3 | 1.66 | 7.79 | 8.94 | 1.35E-13 | 5.61E-11 | 20.61 |
| CENPM | 1.05 | 7.74 | 7.47 | 9.49E-11 | 9.11E-09 | 14.24 |
| CETP | -1.66 | 9.45 | -10.13 | 6.54E-16 | 6.19E-13 | 25.78 |
| CFHR3 | -1.24 | 10.52 | -4.55 | 1.95E-05 | 0.000218 | 2.43 |
| CFHR4 | -1.17 | 9.64 | -4.83 | 6.57E-06 | 8.67E-05 | 3.47 |
| CHST4 | -1.21 | 8.57 | -8.43 | 1.32E-12 | 3.14E-10 | 18.39 |
| CIDEB | -1.13 | 10.62 | -5.53 | 4.04E-07 | 8.41E-06 | 6.15 |
| CKAP2L | 1.04 | 7.40 | 7.46 | 9.92E-11 | 9.49E-09 | 14.20 |
| CKS2 | 1.19 | 9.49 | 7.08 | 5.27E-10 | 3.52E-08 | 12.57 |
| CLDN15 | 1.18 | 10.03 | 6.34 | 1.34E-08 | 4.84E-07 | 9.44 |
| CLEC1B | -2.82 | 8.83 | -12.31 | 5.14E-20 | 2.70E-16 | 34.93 |
| CLEC4G | -2.37 | 9.22 | -14.12 | 2.84E-23 | 1.34E-18 | 42.13 |
| CLRN3 | -1.56 | 10.01 | -5.68 | 2.15E-07 | 5.00E-06 | 6.76 |
| CLYBL | -1.02 | 10.99 | -5.22 | 1.45E-06 | 2.40E-05 | 4.92 |
| CNDP1 | -1.52 | 8.35 | -8.62 | 5.48E-13 | 1.57E-10 | 19.25 |
| COL15A1 | 1.25 | 7.45 | 8.79 | 2.59E-13 | 8.71E-11 | 19.97 |
| COL1A1 | 1.12 | 9.39 | 4.26 | 5.69E-05 | 0.000539 | 1.42 |
| COL1A2 | 1.31 | 9.24 | 5.30 | 1.03E-06 | 1.80E-05 | 5.25 |
| COL4A1 | 1.79 | 11.01 | 9.48 | 1.17E-14 | 6.99E-12 | 22.99 |
| COLEC11 | -1.40 | 10.85 | -6.23 | 2.11E-08 | 7.09E-07 | 9.00 |
| COX7B2 | 1.11 | 7.59 | 4.25 | 5.82E-05 | 0.000549 | 1.39 |
| CPEB3 | -1.56 | 10.33 | -10.83 | 3.02E-17 | 5.11E-14 | 28.76 |
| CPED1 | -1.26 | 9.64 | -8.09 | 6.10E-12 | 1.04E-09 | 16.91 |
| CPS1 | -1.11 | 13.92 | -4.31 | 4.74E-05 | 0.000461 | 1.59 |
| CRHBP | -1.89 | 9.08 | -11.09 | 9.57E-18 | 2.16E-14 | 29.88 |
| CRP | -1.82 | 12.51 | -3.95 | 0.000168 | 0.001362 | 0.39 |
| CSRNP1 | -1.65 | 10.98 | -8.64 | 5.02E-13 | 1.48E-10 | 19.33 |
| CSTB | 1.04 | 11.66 | 6.94 | 9.89E-10 | 5.72E-08 | 11.96 |
| CTH | -1.47 | 10.40 | -6.10 | 3.73E-08 | 1.14E-06 | 8.45 |
| CTNNA1 | 1.05 | 10.39 | 8.28 | 2.59E-12 | 5.31E-10 | 17.74 |
| CXCL12 | -1.37 | 9.30 | -8.49 | 9.86E-13 | 2.55E-10 | 18.68 |
| CXCL14 | -1.16 | 8.46 | -10.22 | 4.37E-16 | 4.50E-13 | 26.17 |
| CXCL2 | -1.66 | 9.94 | -8.01 | 8.63E-12 | 1.36E-09 | 16.57 |
| CYP1A1 | -1.18 | 8.30 | -4.89 | 5.25E-06 | 7.20E-05 | 3.69 |
| CYP1A2 | -4.30 | 11.41 | -10.18 | 5.31E-16 | 5.13E-13 | 25.99 |
| CYP26A1 | -1.74 | 9.45 | -6.87 | 1.33E-09 | 7.22E-08 | 11.68 |
| CYP2A6 | -1.89 | 11.14 | -4.39 | 3.47E-05 | 0.000354 | 1.89 |
| CYP2A7 | -1.74 | 10.01 | -4.97 | 3.86E-06 | 5.53E-05 | 3.98 |
| CYP2C18 | -1.11 | 11.24 | -4.54 | 1.98E-05 | 0.000221 | 2.42 |
| CYP2C8 | -1.70 | 13.93 | -6.35 | 1.29E-08 | 4.70E-07 | 9.48 |
| CYP2C9 | -1.78 | 11.78 | -7.36 | 1.53E-10 | 1.31E-08 | 13.77 |
| CYP2E1 | -2.30 | 14.23 | -5.83 | 1.16E-07 | 2.93E-06 | 7.35 |
| CYP39A1 | -1.55 | 9.25 | -8.90 | 1.55E-13 | 6.11E-11 | 20.47 |
| CYP3A4 | -3.41 | 11.62 | -7.63 | 4.65E-11 | 5.19E-09 | 14.93 |
| CYP3A43 | -1.63 | 9.10 | -7.69 | 3.54E-11 | 4.16E-09 | 15.20 |
| CYP3A7 | -1.43 | 10.87 | -4.55 | 1.90E-05 | 0.000213 | 2.46 |
| CYP4A11 | -1.29 | 14.01 | -6.46 | 8.13E-09 | 3.23E-07 | 9.92 |
| CYP4F12 | -1.19 | 10.36 | -7.02 | 7.09E-10 | 4.44E-08 | 12.29 |
| CYP4F2 | -1.18 | 10.22 | -6.94 | 9.74E-10 | 5.66E-08 | 11.98 |
| CYP4V2 | -1.17 | 11.89 | -6.11 | 3.52E-08 | 1.09E-06 | 8.51 |
| CYP8B1 | -1.16 | 12.50 | -3.49 | 0.000797 | 0.005118 | -1.06 |
| CYR61 | -1.40 | 10.43 | -5.82 | 1.23E-07 | 3.10E-06 | 7.29 |
| DBH | -2.05 | 8.57 | -11.24 | 5.01E-18 | 1.25E-14 | 30.50 |
| DCN | -2.47 | 11.40 | -7.44 | 1.08E-10 | 1.00E-08 | 14.11 |
| DCXR | -1.14 | 13.31 | -5.68 | 2.14E-07 | 4.97E-06 | 6.76 |
| DLC1 | -1.18 | 8.81 | -7.20 | 3.16E-10 | 2.30E-08 | 13.07 |
| DNAJC12 | -1.24 | 9.82 | -6.86 | 1.38E-09 | 7.46E-08 | 11.64 |
| DNASE1L3 | -1.76 | 10.13 | -8.14 | 4.73E-12 | 8.52E-10 | 17.15 |
| DPT | -1.27 | 8.57 | -6.99 | 8.03E-10 | 4.86E-08 | 12.17 |
| ECHS1 | -1.19 | 13.09 | -9.08 | 7.17E-14 | 3.36E-11 | 21.22 |
| ECM1 | -1.85 | 9.01 | -13.09 | 1.95E-21 | 2.31E-17 | 38.08 |
| EEF1A2 | 1.14 | 8.45 | 3.25 | 0.001713 | 0.009596 | -1.77 |
| EGR1 | -1.68 | 11.81 | -7.02 | 6.96E-10 | 4.39E-08 | 12.30 |
| ENO3 | -2.08 | 10.93 | -8.18 | 4.03E-12 | 7.44E-10 | 17.31 |
| EPB41L4B | -1.05 | 8.81 | -7.74 | 2.90E-11 | 3.59E-09 | 15.39 |
| ERRFI1 | -1.11 | 12.53 | -6.83 | 1.61E-09 | 8.39E-08 | 11.49 |
| ESM1 | 1.00 | 8.30 | 8.13 | 5.11E-12 | 8.99E-10 | 17.08 |
| ESR1 | -1.23 | 8.95 | -10.92 | 1.97E-17 | 3.73E-14 | 29.18 |
| ETFDH | -1.09 | 10.76 | -7.55 | 6.72E-11 | 6.84E-09 | 14.57 |
| EVC2 | -1.27 | 9.12 | -6.88 | 1.27E-09 | 6.97E-08 | 11.72 |
| EXOC3L4 | -1.31 | 9.74 | -8.21 | 3.49E-12 | 6.68E-10 | 17.45 |
| F9 | -1.73 | 11.93 | -6.67 | 3.26E-09 | 1.51E-07 | 10.81 |
| FABP4 | 1.04 | 8.16 | 4.89 | 5.30E-06 | 7.26E-05 | 3.68 |
| FAM134B | -1.66 | 9.45 | -5.58 | 3.36E-07 | 7.23E-06 | 6.33 |
| FAM13A | -1.57 | 9.51 | -12.14 | 1.05E-19 | 4.14E-16 | 34.24 |
| FAM189B | 1.11 | 9.35 | 8.47 | 1.09E-12 | 2.77E-10 | 18.58 |
| FAM83D | 1.25 | 7.75 | 9.46 | 1.27E-14 | 7.50E-12 | 22.91 |
| FBP1 | -1.46 | 13.19 | -7.35 | 1.59E-10 | 1.34E-08 | 13.74 |
| FCN2 | -1.90 | 8.69 | -13.60 | 2.32E-22 | 5.49E-18 | 40.12 |
| FCN3 | -2.71 | 9.48 | -11.06 | 1.10E-17 | 2.37E-14 | 29.74 |
| FETUB | -1.08 | 11.34 | -4.13 | 9.02E-05 | 0.000805 | 0.98 |
| FGA | -1.00 | 12.20 | -4.84 | 6.35E-06 | 8.43E-05 | 3.50 |
| FGB | -1.10 | 13.12 | -5.42 | 6.34E-07 | 1.22E-05 | 5.71 |
| FGG | -1.12 | 13.12 | -4.72 | 1.01E-05 | 0.000125 | 3.06 |
| FNDC5 | -1.21 | 9.97 | -4.14 | 8.78E-05 | 0.000786 | 1.01 |
| FOS | -2.54 | 11.33 | -8.97 | 1.17E-13 | 5.13E-11 | 20.75 |
| FOSB | -2.82 | 10.39 | -8.71 | 3.79E-13 | 1.18E-10 | 19.60 |
| FOXO1 | -1.12 | 9.84 | -6.95 | 9.36E-10 | 5.50E-08 | 12.02 |
| FST | -1.07 | 9.20 | -5.91 | 8.30E-08 | 2.22E-06 | 7.68 |
| FTCD | -1.55 | 10.29 | -7.21 | 2.99E-10 | 2.22E-08 | 13.13 |
| FXYD1 | -1.41 | 9.55 | -6.96 | 8.95E-10 | 5.31E-08 | 12.06 |
| G6PC | -1.32 | 11.84 | -5.46 | 5.41E-07 | 1.07E-05 | 5.87 |
| GABARAPL1 | -1.19 | 10.95 | -7.21 | 3.08E-10 | 2.25E-08 | 13.10 |
| GADD45B | -1.58 | 10.63 | -8.07 | 6.63E-12 | 1.10E-09 | 16.82 |
| GADD45G | -1.68 | 10.57 | -8.32 | 2.17E-12 | 4.73E-10 | 17.91 |
| GBA3 | -1.69 | 10.62 | -7.04 | 6.28E-10 | 4.06E-08 | 12.40 |
| GBP2 | 1.09 | 10.36 | 6.77 | 2.06E-09 | 1.03E-07 | 11.25 |
| GCDH | -1.20 | 10.43 | -7.76 | 2.59E-11 | 3.32E-09 | 15.50 |
| GCGR | -1.92 | 11.13 | -5.16 | 1.82E-06 | 2.91E-05 | 4.70 |
| GGH | 1.03 | 11.71 | 6.79 | 1.95E-09 | 9.86E-08 | 11.31 |
| GHR | -1.84 | 11.29 | -7.57 | 6.02E-11 | 6.29E-09 | 14.68 |
| GLS2 | -1.70 | 10.07 | -6.05 | 4.57E-08 | 1.36E-06 | 8.25 |
| GLYAT | -1.34 | 8.75 | -6.93 | 1.03E-09 | 5.91E-08 | 11.92 |
| GLYATL1 | -1.10 | 9.39 | -8.03 | 7.85E-12 | 1.26E-09 | 16.66 |
| GNAO1 | -1.05 | 8.30 | -9.09 | 6.67E-14 | 3.19E-11 | 21.29 |
| GNE | -1.04 | 10.93 | -7.75 | 2.69E-11 | 3.40E-09 | 15.47 |
| GNMT | -1.88 | 10.34 | -6.44 | 8.83E-09 | 3.46E-07 | 9.84 |
| GOT2 | -1.00 | 12.77 | -6.95 | 9.31E-10 | 5.48E-08 | 12.02 |
| GPC3 | 3.19 | 8.88 | 8.28 | 2.54E-12 | 5.24E-10 | 17.76 |
| GPT | -1.35 | 10.79 | -7.00 | 7.67E-10 | 4.71E-08 | 12.21 |
| GPT2 | -1.58 | 11.89 | -7.20 | 3.09E-10 | 2.26E-08 | 13.09 |
| GSTA2 | -1.67 | 10.71 | -5.18 | 1.68E-06 | 2.73E-05 | 4.78 |
| GSTZ1 | -1.33 | 9.57 | -7.86 | 1.65E-11 | 2.30E-09 | 15.94 |
| H19 | -1.42 | 11.52 | -3.74 | 0.000344 | 0.002501 | -0.28 |
| H2AFZ | 1.03 | 11.31 | 6.47 | 7.66E-09 | 3.08E-07 | 9.98 |
| HABP2 | -1.19 | 11.65 | -5.96 | 6.65E-08 | 1.85E-06 | 7.89 |
| HAL | -1.22 | 10.26 | -5.50 | 4.66E-07 | 9.43E-06 | 6.01 |
| HAMP | -4.58 | 12.54 | -11.31 | 3.65E-18 | 1.02E-14 | 30.81 |
| HAO2 | -1.31 | 9.43 | -7.66 | 4.17E-11 | 4.77E-09 | 15.04 |
| HBA1 | -1.51 | 10.73 | -5.40 | 6.76E-07 | 1.28E-05 | 5.65 |
| HBA2 | -1.68 | 11.69 | -6.41 | 9.87E-09 | 3.78E-07 | 9.73 |
| HBB | -1.47 | 12.06 | -5.47 | 5.09E-07 | 1.01E-05 | 5.93 |
| HEPACAM | -1.56 | 9.96 | -5.58 | 3.30E-07 | 7.11E-06 | 6.34 |
| HGFAC | -2.99 | 10.67 | -10.83 | 2.92E-17 | 5.11E-14 | 28.80 |
| HKDC1 | 1.10 | 8.99 | 4.91 | 4.91E-06 | 6.80E-05 | 3.75 |
| HMMR | 1.02 | 7.42 | 9.55 | 8.59E-15 | 5.42E-12 | 23.28 |
| HOGA1 | -1.29 | 9.47 | -8.83 | 2.15E-13 | 7.61E-11 | 20.15 |
| HPD | -1.12 | 12.83 | -3.82 | 0.000263 | 0.001991 | -0.03 |
| HPX | -1.10 | 14.50 | -4.22 | 6.59E-05 | 0.000611 | 1.28 |
| HSD11B1 | -1.20 | 12.42 | -3.41 | 0.001038 | 0.006378 | -1.31 |
| HSD17B13 | -1.90 | 9.70 | -6.94 | 9.70E-10 | 5.66E-08 | 11.98 |
| HSD17B2 | -1.26 | 11.28 | -6.12 | 3.39E-08 | 1.06E-06 | 8.54 |
| ID1 | -1.42 | 10.12 | -6.24 | 2.06E-08 | 6.93E-07 | 9.02 |
| IDO2 | -1.42 | 9.10 | -8.56 | 7.44E-13 | 2.02E-10 | 18.95 |
| IGFALS | -2.26 | 9.66 | -12.14 | 1.02E-19 | 4.14E-16 | 34.26 |
| IGFBP1 | -1.37 | 10.89 | -5.14 | 1.96E-06 | 3.09E-05 | 4.63 |
| IGFBP3 | -1.84 | 11.78 | -10.72 | 4.73E-17 | 7.46E-14 | 28.33 |
| IGLL1 | -1.82 | 11.13 | -4.82 | 6.75E-06 | 8.86E-05 | 3.45 |
| IL1RAP | -1.34 | 9.01 | -7.63 | 4.64E-11 | 5.19E-09 | 14.93 |
| ILF2 | 1.04 | 10.73 | 7.14 | 4.14E-10 | 2.92E-08 | 12.81 |
| INMT | -1.39 | 8.63 | -6.38 | 1.15E-08 | 4.29E-07 | 9.59 |
| IRX3 | 1.44 | 8.56 | 5.52 | 4.16E-07 | 8.61E-06 | 6.12 |
| KBTBD11 | -1.88 | 8.96 | -9.24 | 3.46E-14 | 1.80E-11 | 21.93 |
| KCNN2 | -1.71 | 8.79 | -11.00 | 1.41E-17 | 2.90E-14 | 29.50 |
| KDM8 | -1.37 | 9.11 | -8.75 | 3.16E-13 | 1.02E-10 | 19.78 |
| KIAA0101 | 1.43 | 8.08 | 8.58 | 6.58E-13 | 1.82E-10 | 19.07 |
| KLKB1 | -1.74 | 11.36 | -8.70 | 3.87E-13 | 1.20E-10 | 19.58 |
| KMO | -1.54 | 9.58 | -9.58 | 7.59E-15 | 4.85E-12 | 23.40 |
| LAPTM4B | 1.20 | 10.33 | 4.93 | 4.41E-06 | 6.21E-05 | 3.85 |
| LCAT | -1.91 | 9.39 | -10.21 | 4.47E-16 | 4.50E-13 | 26.15 |
| LCN2 | 1.29 | 9.62 | 3.71 | 0.000383 | 0.002741 | -0.38 |
| LDHD | -1.00 | 9.84 | -6.19 | 2.52E-08 | 8.21E-07 | 8.83 |
| LECT2 | -1.68 | 11.36 | -5.63 | 2.68E-07 | 6.00E-06 | 6.54 |
| LIPC | -1.13 | 11.63 | -5.73 | 1.76E-07 | 4.21E-06 | 6.95 |
| LPA | -1.33 | 9.11 | -6.20 | 2.49E-08 | 8.12E-07 | 8.84 |
| LRRC1 | 1.00 | 8.62 | 6.38 | 1.15E-08 | 4.29E-07 | 9.59 |
| LY6E | -1.74 | 12.05 | -6.42 | 9.70E-09 | 3.73E-07 | 9.75 |
| LYVE1 | -1.05 | 8.48 | -8.54 | 7.87E-13 | 2.13E-10 | 18.90 |
| LYZ | 1.63 | 9.95 | 4.99 | 3.48E-06 | 5.06E-05 | 4.08 |
| MARCKS | 1.03 | 10.72 | 5.95 | 6.91E-08 | 1.91E-06 | 7.85 |
| MARCO | -1.59 | 8.66 | -11.46 | 1.94E-18 | 5.73E-15 | 31.42 |
| MASP1 | -1.24 | 8.79 | -10.42 | 1.76E-16 | 2.09E-13 | 27.05 |
| MAT1A | -1.41 | 11.86 | -7.80 | 2.21E-11 | 2.93E-09 | 15.65 |
| MBL2 | -1.27 | 11.14 | -5.06 | 2.65E-06 | 4.01E-05 | 4.34 |
| MCM2 | 1.05 | 7.95 | 6.58 | 4.79E-09 | 2.07E-07 | 10.43 |
| MCM4 | 1.21 | 8.88 | 6.26 | 1.87E-08 | 6.41E-07 | 9.12 |
| MDK | 1.79 | 9.44 | 7.39 | 1.37E-10 | 1.22E-08 | 13.88 |
| MELK | 1.03 | 8.11 | 8.62 | 5.47E-13 | 1.57E-10 | 19.25 |
| MFAP4 | -1.46 | 9.22 | -7.14 | 4.17E-10 | 2.93E-08 | 12.80 |
| MMP9 | 1.08 | 7.90 | 3.72 | 0.000376 | 0.002691 | -0.36 |
| MNS1 | 1.08 | 8.11 | 5.65 | 2.45E-07 | 5.56E-06 | 6.63 |
| MSRA | -1.04 | 10.31 | -5.78 | 1.45E-07 | 3.54E-06 | 7.14 |
| MT1A | -2.01 | 13.80 | -7.49 | 8.78E-11 | 8.50E-09 | 14.31 |
| MT1E | -2.39 | 13.21 | -7.66 | 4.03E-11 | 4.66E-09 | 15.07 |
| MT1F | -3.00 | 11.71 | -8.16 | 4.41E-12 | 8.02E-10 | 17.22 |
| MT1G | -3.04 | 13.21 | -7.86 | 1.66E-11 | 2.30E-09 | 15.93 |
| MT1H | -3.22 | 11.23 | -7.63 | 4.67E-11 | 5.20E-09 | 14.93 |
| MT1M | -2.75 | 10.18 | -7.12 | 4.54E-10 | 3.12E-08 | 12.72 |
| MT1X | -2.30 | 13.39 | -7.53 | 7.37E-11 | 7.35E-09 | 14.49 |
| MT2A | -1.97 | 13.76 | -7.19 | 3.33E-10 | 2.40E-08 | 13.02 |
| MTMR11 | 1.00 | 8.61 | 6.37 | 1.18E-08 | 4.37E-07 | 9.57 |
| MUC13 | 1.16 | 8.43 | 4.66 | 1.28E-05 | 0.000153 | 2.84 |
| MYOM2 | -1.10 | 8.71 | -7.35 | 1.62E-10 | 1.36E-08 | 13.72 |
| NAT2 | -1.77 | 9.44 | -9.10 | 6.50E-14 | 3.14E-11 | 21.32 |
| NCAPG | 1.39 | 7.74 | 9.01 | 9.80E-14 | 4.38E-11 | 20.92 |
| NDRG2 | -1.32 | 10.46 | -9.28 | 2.93E-14 | 1.61E-11 | 22.09 |
| NDUFA4L2 | 1.34 | 8.00 | 6.74 | 2.41E-09 | 1.17E-07 | 11.10 |
| NEU4 | -1.22 | 9.50 | -4.37 | 3.82E-05 | 0.000385 | 1.79 |
| NME1 | 1.03 | 12.06 | 8.83 | 2.14E-13 | 7.61E-11 | 20.16 |
| NNMT | -2.17 | 12.02 | -6.37 | 1.16E-08 | 4.33E-07 | 9.58 |
| NOCT | -1.06 | 8.82 | -6.38 | 1.12E-08 | 4.20E-07 | 9.61 |
| NPC1L1 | -1.07 | 9.67 | -4.30 | 4.88E-05 | 0.000472 | 1.56 |
| NPW | -2.00 | 10.29 | -6.59 | 4.55E-09 | 1.99E-07 | 10.49 |
| NQO1 | 1.57 | 9.92 | 5.83 | 1.14E-07 | 2.90E-06 | 7.37 |
| NR1I2 | -1.08 | 9.91 | -6.38 | 1.14E-08 | 4.26E-07 | 9.60 |
| NR4A2 | -1.05 | 9.18 | -4.85 | 6.17E-06 | 8.22E-05 | 3.53 |
| NSMCE2 | 1.08 | 8.72 | 9.08 | 7.06E-14 | 3.34E-11 | 21.24 |
| NTHL1 | -1.02 | 10.41 | -7.37 | 1.48E-10 | 1.28E-08 | 13.81 |
| NUSAP1 | 1.56 | 8.65 | 10.54 | 1.06E-16 | 1.35E-13 | 27.55 |
| OAT | -1.21 | 10.13 | -5.20 | 1.56E-06 | 2.56E-05 | 4.85 |
| OGDHL | -1.43 | 10.49 | -7.49 | 8.59E-11 | 8.34E-09 | 14.34 |
| OIT3 | -1.20 | 7.69 | -8.46 | 1.15E-12 | 2.88E-10 | 18.53 |
| OXT | -1.14 | 8.67 | -8.09 | 5.98E-12 | 1.03E-09 | 16.93 |
| PAGE4 | 1.45 | 7.51 | 4.15 | 8.26E-05 | 0.000744 | 1.06 |
| PAMR1 | -1.33 | 8.26 | -9.05 | 7.91E-14 | 3.67E-11 | 21.13 |
| PANK1 | -1.33 | 11.08 | -9.74 | 3.68E-15 | 2.76E-12 | 24.11 |
| PBLD | -1.03 | 10.15 | -6.69 | 2.94E-09 | 1.39E-07 | 10.91 |
| PCK1 | -1.53 | 9.28 | -8.01 | 8.40E-12 | 1.33E-09 | 16.59 |
| PDK4 | -1.29 | 11.26 | -5.76 | 1.56E-07 | 3.77E-06 | 7.07 |
| PEA15 | 1.06 | 10.37 | 7.03 | 6.57E-10 | 4.21E-08 | 12.36 |
| PEMT | -1.17 | 11.58 | -6.97 | 8.69E-10 | 5.16E-08 | 12.09 |
| PEX11G | -1.10 | 9.57 | -9.60 | 6.91E-15 | 4.54E-12 | 23.49 |
| PGLYRP2 | -1.94 | 12.31 | -7.75 | 2.80E-11 | 3.50E-09 | 15.43 |
| PHGDH | -1.46 | 12.29 | -7.83 | 1.93E-11 | 2.63E-09 | 15.79 |
| PHLDA1 | -1.09 | 10.46 | -4.27 | 5.45E-05 | 0.000519 | 1.46 |
| PLA2G16 | -1.35 | 9.22 | -10.86 | 2.65E-17 | 4.83E-14 | 28.89 |
| PLG | -1.02 | 13.69 | -4.80 | 7.47E-06 | 9.66E-05 | 3.35 |
| PLGLB1 | -1.12 | 12.35 | -5.19 | 1.60E-06 | 2.62E-05 | 4.82 |
| PLVAP | 1.14 | 8.45 | 11.28 | 4.13E-18 | 1.08E-14 | 30.69 |
| PNPLA7 | -1.51 | 9.96 | -10.63 | 7.27E-17 | 1.01E-13 | 27.91 |
| PODXL | 1.35 | 8.62 | 9.98 | 1.27E-15 | 1.09E-12 | 25.14 |
| PON1 | -1.23 | 10.61 | -5.20 | 1.55E-06 | 2.55E-05 | 4.85 |
| PPARGC1A | -1.44 | 10.29 | -6.56 | 5.18E-09 | 2.21E-07 | 10.36 |
| PPP1R1A | -1.17 | 10.40 | -5.49 | 4.78E-07 | 9.64E-06 | 5.99 |
| PRC1 | 1.61 | 8.54 | 9.54 | 8.89E-15 | 5.54E-12 | 23.25 |
| PROZ | -1.45 | 9.61 | -5.80 | 1.31E-07 | 3.24E-06 | 7.24 |
| PSAT1 | -1.41 | 11.03 | -6.29 | 1.64E-08 | 5.72E-07 | 9.24 |
| PTGS2 | -1.40 | 8.65 | -6.81 | 1.76E-09 | 9.05E-08 | 11.41 |
| PTH1R | -1.96 | 8.51 | -12.14 | 1.03E-19 | 4.14E-16 | 34.25 |
| PTTG1 | 1.42 | 9.14 | 9.73 | 3.93E-15 | 2.90E-12 | 24.04 |
| PTTG3P | 1.31 | 8.03 | 10.10 | 7.50E-16 | 6.96E-13 | 25.65 |
| PYROXD2 | -1.10 | 8.83 | -4.84 | 6.29E-06 | 8.36E-05 | 3.51 |
| PZP | -1.85 | 9.18 | -5.81 | 1.28E-07 | 3.17E-06 | 7.26 |
| RAB17 | -1.04 | 11.74 | -5.25 | 1.24E-06 | 2.11E-05 | 5.07 |
| RACGAP1 | 1.20 | 7.73 | 10.01 | 1.11E-15 | 9.93E-13 | 25.27 |
| RAD51 | -1.02 | 8.88 | -7.71 | 3.27E-11 | 3.93E-09 | 15.27 |
| RAP2A | 1.15 | 9.01 | 8.24 | 3.08E-12 | 6.15E-10 | 17.57 |
| RBP1 | -1.30 | 10.13 | -5.02 | 3.20E-06 | 4.71E-05 | 4.16 |
| RCAN1 | -1.28 | 11.10 | -6.89 | 1.22E-09 | 6.77E-08 | 11.76 |
| RCL1 | -1.36 | 10.07 | -6.82 | 1.65E-09 | 8.59E-08 | 11.47 |
| RDH16 | -1.47 | 12.80 | -5.31 | 1.01E-06 | 1.77E-05 | 5.27 |
| RDH5 | -1.85 | 10.41 | -10.46 | 1.49E-16 | 1.80E-13 | 27.22 |
| RELN | -1.12 | 9.51 | -3.61 | 0.000545 | 0.003716 | -0.71 |
| RFC4 | 1.08 | 8.55 | 8.13 | 4.91E-12 | 8.73E-10 | 17.12 |
| RFX5 | 1.38 | 9.42 | 9.40 | 1.71E-14 | 9.87E-12 | 22.61 |
| RGS5 | 1.07 | 8.06 | 8.95 | 1.27E-13 | 5.47E-11 | 20.67 |
| RNASE4 | -1.17 | 12.91 | -4.28 | 5.18E-05 | 0.000497 | 1.50 |
| RND3 | -1.64 | 10.14 | -8.85 | 1.94E-13 | 7.21E-11 | 20.26 |
| RNF125 | -1.06 | 9.72 | -7.59 | 5.56E-11 | 5.97E-09 | 14.76 |
| RRS1 | 1.05 | 8.51 | 6.97 | 8.50E-10 | 5.08E-08 | 12.11 |
| S100A10 | 1.16 | 12.48 | 8.16 | 4.30E-12 | 7.86E-10 | 17.25 |
| S100A8 | -1.06 | 9.12 | -4.65 | 1.31E-05 | 0.000157 | 2.81 |
| S100P | 1.25 | 7.75 | 3.72 | 0.000373 | 0.002676 | -0.36 |
| SAA4 | -1.28 | 13.78 | -5.14 | 1.97E-06 | 3.11E-05 | 4.63 |
| SCGN | 1.07 | 7.64 | 4.11 | 9.62E-05 | 0.000851 | 0.92 |
| SDS | -1.95 | 10.99 | -5.36 | 8.10E-07 | 1.48E-05 | 5.48 |
| SERPINA11 | -1.65 | 11.29 | -7.46 | 1.02E-10 | 9.63E-09 | 14.17 |
| SERPINA4 | -1.09 | 9.20 | -6.68 | 3.06E-09 | 1.43E-07 | 10.87 |
| SERPINA5 | -1.12 | 12.23 | -5.94 | 7.29E-08 | 2.00E-06 | 7.80 |
| SERPINF2 | -1.00 | 13.01 | -6.36 | 1.22E-08 | 4.49E-07 | 9.53 |
| SEZ6L2 | 1.32 | 7.61 | 5.01 | 3.27E-06 | 4.79E-05 | 4.14 |
| SHBG | -1.95 | 10.34 | -7.99 | 9.20E-12 | 1.42E-09 | 16.51 |
| SIK1 | -1.07 | 9.53 | -5.09 | 2.42E-06 | 3.70E-05 | 4.43 |
| SKAP1 | -1.38 | 9.60 | -6.47 | 7.80E-09 | 3.12E-07 | 9.96 |
| SLC10A1 | -1.60 | 12.42 | -4.86 | 5.84E-06 | 7.86E-05 | 3.58 |
| SLC13A5 | -1.15 | 12.49 | -5.46 | 5.33E-07 | 1.05E-05 | 5.88 |
| SLC22A1 | -1.99 | 11.16 | -6.42 | 9.65E-09 | 3.71E-07 | 9.76 |
| SLC22A10 | -1.14 | 9.16 | -7.51 | 7.90E-11 | 7.80E-09 | 14.42 |
| SLC25A15 | -1.10 | 11.46 | -6.60 | 4.44E-09 | 1.95E-07 | 10.51 |
| SLC25A25 | -1.15 | 11.70 | -6.09 | 3.83E-08 | 1.17E-06 | 8.42 |
| SLC25A47 | -1.82 | 10.06 | -8.02 | 8.09E-12 | 1.29E-09 | 16.63 |
| SLC27A5 | -1.46 | 11.95 | -5.73 | 1.80E-07 | 4.29E-06 | 6.93 |
| SLC38A4 | -1.10 | 10.12 | -7.37 | 1.47E-10 | 1.28E-08 | 13.81 |
| SLC39A5 | -1.12 | 11.05 | -5.84 | 1.10E-07 | 2.83E-06 | 7.40 |
| SLC7A2 | -1.48 | 11.95 | -6.85 | 1.49E-09 | 7.87E-08 | 11.57 |
| SLCO1B3 | -2.19 | 9.35 | -7.98 | 9.62E-12 | 1.47E-09 | 16.46 |
| SLCO2A1 | 1.08 | 8.02 | 6.81 | 1.75E-09 | 9.01E-08 | 11.41 |
| SMIM24 | -1.45 | 9.99 | -4.97 | 3.79E-06 | 5.45E-05 | 4.00 |
| SNORD13 | -1.48 | 10.34 | -7.94 | 1.16E-11 | 1.70E-09 | 16.28 |
| SOCS2 | -1.86 | 10.46 | -8.30 | 2.29E-12 | 4.89E-10 | 17.86 |
| SORL1 | -1.23 | 10.52 | -8.69 | 4.06E-13 | 1.22E-10 | 19.54 |
| SPINK1 | 3.38 | 10.03 | 6.39 | 1.10E-08 | 4.13E-07 | 9.63 |
| SPP1 | 1.54 | 9.41 | 3.76 | 0.000326 | 0.002391 | -0.23 |
| SPP2 | -1.79 | 12.23 | -5.60 | 3.04E-07 | 6.66E-06 | 6.42 |
| SQLE | 1.93 | 10.12 | 7.23 | 2.75E-10 | 2.08E-08 | 13.21 |
| SRD5A2 | -1.55 | 9.09 | -8.15 | 4.52E-12 | 8.20E-10 | 17.20 |
| SRPX | -1.17 | 8.24 | -7.25 | 2.54E-10 | 1.95E-08 | 13.28 |
| SRXN1 | 1.26 | 9.69 | 7.30 | 2.01E-10 | 1.61E-08 | 13.51 |
| SSR2 | 1.05 | 10.24 | 6.99 | 8.11E-10 | 4.89E-08 | 12.16 |
| SSUH2 | 1.08 | 7.58 | 6.01 | 5.59E-08 | 1.60E-06 | 8.06 |
| ST3GAL6 | -1.06 | 9.55 | -7.18 | 3.48E-10 | 2.49E-08 | 12.98 |
| STAB2 | -1.56 | 8.55 | -12.55 | 1.83E-20 | 1.17E-16 | 35.92 |
| STARD5 | -1.11 | 10.00 | -8.18 | 3.94E-12 | 7.32E-10 | 17.33 |
| STEAP3 | -1.35 | 10.69 | -7.61 | 5.13E-11 | 5.58E-09 | 14.84 |
| SULT1A1 | -1.05 | 11.05 | -6.12 | 3.49E-08 | 1.08E-06 | 8.51 |
| TACSTD2 | -1.51 | 8.33 | -5.99 | 5.95E-08 | 1.69E-06 | 8.00 |
| TAT | -2.10 | 12.05 | -5.98 | 6.28E-08 | 1.77E-06 | 7.95 |
| TDO2 | -1.20 | 12.87 | -4.31 | 4.75E-05 | 0.000462 | 1.59 |
| TFRC | 1.12 | 10.80 | 6.71 | 2.66E-09 | 1.27E-07 | 11.00 |
| THRSP | -1.78 | 11.09 | -4.90 | 4.97E-06 | 6.88E-05 | 3.74 |
| THY1 | 1.55 | 8.41 | 9.03 | 8.81E-14 | 3.97E-11 | 21.02 |
| TK1 | 1.10 | 8.06 | 8.30 | 2.30E-12 | 4.89E-10 | 17.85 |
| TKT | 1.23 | 11.10 | 7.02 | 6.97E-10 | 4.39E-08 | 12.30 |
| TMCO3 | 1.10 | 8.65 | 7.81 | 2.09E-11 | 2.80E-09 | 15.71 |
| TMEM27 | -1.31 | 9.06 | -8.23 | 3.19E-12 | 6.35E-10 | 17.53 |
| TMEM45B | 1.32 | 8.70 | 5.78 | 1.44E-07 | 3.51E-06 | 7.15 |
| TOMM40L | 1.16 | 8.36 | 8.95 | 1.26E-13 | 5.45E-11 | 20.68 |
| TOP2A | 2.22 | 8.28 | 10.69 | 5.49E-17 | 8.12E-14 | 28.18 |
| TP53I3 | 1.22 | 8.48 | 7.66 | 4.15E-11 | 4.76E-09 | 15.04 |
| TPR | -1.38 | 12.31 | -4.43 | 3.02E-05 | 0.000315 | 2.02 |
| TRIB1 | -1.14 | 12.44 | -8.18 | 3.91E-12 | 7.29E-10 | 17.34 |
| TRPM8 | -1.08 | 8.94 | -7.37 | 1.48E-10 | 1.28E-08 | 13.81 |
| TTC36 | -1.08 | 8.09 | -10.81 | 3.27E-17 | 5.34E-14 | 28.69 |
| TUBA1C | 1.43 | 11.46 | 7.92 | 1.28E-11 | 1.83E-09 | 16.19 |
| TXNRD1 | 1.33 | 9.69 | 7.29 | 2.13E-10 | 1.69E-08 | 13.45 |
| UBD | 2.21 | 11.21 | 7.26 | 2.39E-10 | 1.85E-08 | 13.34 |
| UBE2C | 1.94 | 8.20 | 8.74 | 3.24E-13 | 1.04E-10 | 19.76 |
| UGP2 | -1.21 | 11.15 | -7.21 | 3.05E-10 | 2.25E-08 | 13.11 |
| UGT2B10 | -1.53 | 12.83 | -5.86 | 1.01E-07 | 2.62E-06 | 7.49 |
| VIPR1 | -1.96 | 9.36 | -12.92 | 3.85E-21 | 3.65E-17 | 37.42 |
| VNN1 | -1.10 | 10.02 | -4.63 | 1.45E-05 | 0.000169 | 2.72 |
| VWF | 1.32 | 10.57 | 5.65 | 2.47E-07 | 5.60E-06 | 6.62 |
| WDR72 | -1.69 | 10.43 | -6.35 | 1.30E-08 | 4.74E-07 | 9.47 |
| ZFP36 | -1.14 | 12.07 | -6.46 | 8.15E-09 | 3.23E-07 | 9.92 |
| ZG16 | -1.20 | 9.26 | -8.02 | 8.27E-12 | 1.31E-09 | 16.61 |
| ZGPAT | -1.70 | 11.49 | -9.85 | 2.23E-15 | 1.76E-12 | 24.59 |
